# Membrane-targeted schiff base derivatives overcome MRSA resistance through phosphatidylglycerol binding and ROS-mediated killing

**DOI:** 10.3389/fchem.2026.1753350

**Published:** 2026-03-04

**Authors:** Yaguang Liu, Lianzhi Hu, Binbin Liu, Zheng Qu

**Affiliations:** The Second Hospital of QinHuangDao, Pharmacy Department, QinHuangDao, China,

**Keywords:** anti-biofilm, antimicrobial, druglikeness, membrane-targeting, schiff bases

## Abstract

**Introduction:**

The urgent need for novel antibacterial agents against drug-resistant Gram-positive pathogens, particularly Methicillin-resistant Staphylococcus aureus (MRSA), drives this research. This study aimed to synthesize and evaluate a series of N’-substituted methylene-4-chlorobenzohydrazide derivatives as potential anti-MRSA agents.

**Methods:**

Sixteen target compounds **(C1–C16)** were synthesized from commercial ethyl 4-chlorobenzoate via ester aminolysis and condensation. Their structures were confirmed by ^1^H NMR, ^13^C NMR, and HRMS. Biological evaluations included *in vitro* antibacterial assays against a panel of bacteria, cytotoxicity (VERO cells), hemolytic activity, mechanistic studies (membrane targeting, depolarization, permeability, content leakage, ROS generation), biofilm inhibition, and resistance development assessment. Drug-likeness properties were also analyzed.

**Results:**

Two novel **(C1, C16)** and fourteen known analogues were obtained. The series showed weak activity against Gram-negative bacteria but potent inhibition against various Gram-positive bacteria, including MRSA. Compound **C12** emerged as the optimal derivative, exhibiting the strongest broad-spectrum anti-Gram-positive activity (MIC = 26 μM) and high selectivity. **C12** showed no significant cytotoxicity or hemolysis at effective concentrations. It specifically targeted phosphatidylglycerol (PG) in the bacterial membrane, causing rapid membrane depolarization, increased permeability, leakage of intracellular proteins/DNA, ROS burst, and bactericidal effects. Furthermore, **C12** inhibited S. aureus biofilm formation and displayed a very low propensity for spontaneous resistance development. It demonstrated moderate metabolic stability and suitable lipophilicity.

**Discussion:**

Compound **C12** represents a promising anti-MRSA lead compound. It combines potent antibacterial activity with a unique multi-mechanistic action targeting the cell membrane, a favorable biosafety profile, and a low resistance risk. These merits warrant further in-depth investigation and development.

## 1 Introduction

In the protracted struggle between humans and pathogenic bacteria, the discovery and application of antibiotics once represented a milestone breakthrough, significantly reducing mortality from infectious diseases (Hall and Mah, 2017; Huemer et al., 2020). However, with the widespread misuse of these “miracle drugs,” we are now confronting a global public health crisis—antimicrobial resistance (AMR)—that threatens to drag humanity back to the “pre-antibiotic era” (Diallo et al., 2017; Luo et al., 2019). Data indicates that millions of global deaths annually are linked to bacterial resistance, prompting the World Health Organization to classify it as a threat on par with climate change (Pulingam et al., 2022). The root of resistance lies in the powerful evolutionary capacity of bacteria. Through mechanisms such as producing drug-inactivating enzymes, altering drug targets, upregulating efflux pumps, and reducing membrane permeability, bacteria have given rise to “superbugs” like Methicillin-resistant *Staphylococcus aureus* (MRSA) and Carbapenem-resistant Enterobacteriaceae (CRE) (Schillaci et al., 2017; O'Neill et al., 2019; Bush et al., 2020). An even more daunting challenge is that in natural and infection environments, bacteria predominantly exist in the form of biofilms—structured communities encased in a matrix of polysaccharides, proteins, and extracellular DNA (Solano et al., 2014). Biofilms form robust physical and physiological barriers. Within them, nutrient and oxygen gradients create metabolic heterogeneity, particularly rendering dormant “persister cells” highly tolerant to most antibiotics. This leads to refractory clinical conditions such as chronic wound infections, lung infections, and medical device-associated infections (Del, 2018; Rabin et al., 2015). With the current antibiotic development pipeline increasingly drying up and bacterial evolution continuing unabated, the situation urgently demands that we break free from traditional frameworks. There is a pressing need to develop novel antimicrobial agents with new scaffolds or mechanisms of action that can effectively combat biofilms, in order to address this existential challenge to human health.

In this challenging field of research, Schiff bases are experiencing renewed vitality in antimicrobial drug development due to their unique physicochemical properties and diverse biological activities (Dutta and Halder, 2022; Kasare et al., 2022). Formed by the condensation of aldehydes or ketones with primary amines, these C=N-containing compounds offer straightforward synthesis and high structural tunability. The flexible combination of different carbonyl and amine modules allows for the rapid construction of diverse molecular libraries, greatly facilitating lead compound screening (Jia et al., 2022). The core C=N bond is not only a stable pharmacophore that can act as a hydrogen bond acceptor to interact with biological targets but also possesses “metabolic switch” characteristics, being hydrolyzable under specific conditions to release the original components, enabling targeted delivery. Furthermore, this group can effectively chelate metal ions, disrupting the function of bacterial key enzymes that rely on metallic cofactors (Kaur et al., 2019; Liu et al., 2023). Studies have shown that Schiff base compounds exhibit broad-spectrum antibacterial effects through multi-target mechanisms. These include disrupting cell membrane integrity, inhibiting key enzymes such as dihydrofolate reductase, inducing metal starvation via chelation, and interfering with quorum-sensing systems. The latter represents an “anti-virulence” strategy capable of suppressing virulence factor production and biofilm formation. Since this approach does not directly kill bacteria, it helps delay the development of resistance (Frei et al., 2021; Gao et al., 2020; Tian et al., 2023). However, despite numerous reports, most studies remain limited to preliminary *in vitro* antibacterial screening. There is a notable lack of systematic evaluation of critical parameters such as selective toxicity (hemolytic activity and cytotoxicity), risk of resistance induction, anti-biofilm efficacy, and drug-likeness. This comprehensive research gap severely hinders objective assessment of the clinical translation potential of Schiff base compounds and constitutes the core scientific problem addressed in this study.

**FIGURE 1:**
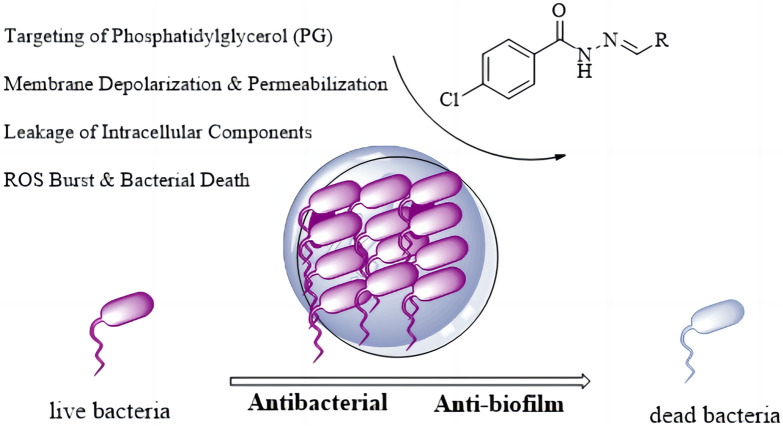
Antibacterial mechanism diagram of Isoniazid Schiff Base derivatives.

Based on the aforementioned background, and in response to the dual threats of antibiotic resistance and biofilm infections, this study is grounded in the enduringly valuable Schiff base pharmacophore. We designed and executed a comprehensive research strategy encompassing rational molecular design, efficient chemical synthesis, and multi-level systematic biological evaluation. Selecting 4-chlorobenzohydrazide as a key amine building block, we constructed a structurally diverse and distinctive library of Schiff base derivatives via efficient condensation reactions with eighteen structurally diverse aldehydes (including aromatic, heteroaromatic, and aliphatic aldehydes). This library provides a solid material foundation for in-depth structure-activity relationship (SAR) studies. N′-substituted methylene-4-chlorobenzohydrazide derivatives (**C1**–**C16**) were rationally designed by integrating key pharmacophores anticipated to be crucial for interaction with the penicillin-binding protein 2a (PBP2a) (Ambade et al., 2023; Dai et al., 2023). The design centers on two core structural motifs: (1) The 4-chlorobenzoyl scaffold was selected as a privileged fragment. The para-chloro substituent serves a dual purpose: (i) its strong electron-withdrawing nature fine-tunes the electron density of the aromatic ring, potentially enhancing the stability of the adjacent hydrazone bond and optimizing the molecule’s lipophilicity for membrane permeation; and crucially, (ii) the chlorine atom itself acts as a versatile pharmacophore, capable of forming specific halogen bonds with carbonyl oxygens or π-systems in the target pocket, and participating in hydrophobic interactions to improve binding affinity and selectivity. (2) The hydrazone linker (-NHN = CH-) is a well-established bioactive pharmacophore. It not only provides conformational rigidity but also offers multiple sites for hydrogen bonding. The imine nitrogen can act as a hydrogen bond acceptor, while the adjacent -NH- proton serves as a key hydrogen bond donor, enabling critical interactions with amino acid residues (e.g., backbone carbonyls or side-chain donors/acceptors) in the receptor’s active site. The variable aldehyde-derived substituents (R) were introduced to probe steric and electronic effects, and to explore additional interactions with peripheral regions of the binding pocket, thereby optimizing potency and selectivity. The ultimate aim of this work is to provide valuable chemical entities, robust experimental data, and innovative R&D strategies for developing novel therapeutic agents against superbugs and recalcitrant biofilm-associated infections (Figure 1).

## 2 Results and discussion

### 2.1 Chemical synthesis

**SCHEME 1:**
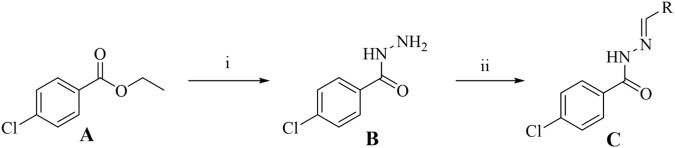
Synthesis of Isoniazid derivatives. Conditions and reagents: (i) Ethanol, NH_2_NH_2_⋅H_2_O, reflux, yield 86%; (ii) Ethanol, R-CHO, reflux, yield 81-91%. **(A)** (ethyl 4-chlorobenzoate), **(B)** (4-chlorobenzhydrazide), **(C)** **(C1–C12)**.

Using commercially available ethyl 4-chlorobenzoate (**A**) as the starting material and referring to literature methods (Liu et al., 2025; Tan et al., 2022), a series of *N*′-substituted methylene-4-chlorobenzohydrazide derivatives (**C1**–**C16**) were successfully designed and synthesized. The general synthetic route is illustrated in Scheme 1. Among the synthesized compounds, **C1** and **C16** are novel structures that have not been previously reported in the literature. The remaining compounds (**C2**–**C15**) are known and have been reported previously (Abdel-Aziz et al., 2021; Al-Wahaibi et al., 2024; Khan et al., 2018). The synthesis of the key intermediate, 4-chlorobenzohydrazide (**B**), was achieved via an efficient ester aminolysis reaction. Specifically, compound **A** was heated under reflux with an excess of hydrazine monohydrate in ethanol. The reaction progress was monitored dynamically by thin-layer chromatography (TLC). Upon completion, the mixture was cooled to room temperature, resulting in the quantitative precipitation of intermediate **B** as a high-purity white solid. Simple filtration, washing with cold ethanol, and vacuum drying afforded pure product suitable for the subsequent step without the need for tedious column chromatography. This method is straightforward, provides high yield (86%), and demonstrates practicality and cost-effectiveness for large-scale preparation. After obtaining key intermediate **B**, the characteristic bioactive pharmacophore–the hydrazone bond (-NHN = CH-) – of the target molecules was successfully constructed via condensation reactions with 16 structurally diverse aldehydes. This step was performed under reflux in anhydrous ethanol. Leveraging the inherent strong nucleophilicity of the hydrazide, the reactions proceeded efficiently and reached completion within 8 h, as confirmed by TLC. After cooling the reaction mixtures, most target compounds (**C1**–**C16**) precipitated directly and were obtained as high-purity samples meeting analytical requirements through recrystallization (ethanol/water system). The chemical structures of all final compounds were unambiguously confirmed using modern spectroscopic techniques, including ^1^H NMR, ^13^C NMR, and High-Resolution Mass Spectrometry (HRMS). In the ^1^H NMR spectra of all target compounds, the characteristic imine proton signal adjacent to the hydrazone bond was observed as a sharp singlet or broad peak in the range of δ 7.60–8.40 ppm, providing definitive evidence for the formation of the hydrazone structure. Concurrently, the -NH- proton signal adjacent to the carbonyl group in the hydrazide fragment was also clearly identifiable, typically appearing in the downfield region of δ 10.20–11.20 ppm. Furthermore, HRMS analysis provided precise molecular weight evidence for all target molecules. The detected molecular ion peaks [M + H]^+^ matched the theoretically calculated values, offering indisputable confirmation of the correct molecular formulas at the mass spectrometry level.

### 2.2 Determination of minimum inhibitory concentration

According to literature reports, certain Schiff base derivatives exhibit promising *in vitro* antibacterial activity (Chung., 2022; Sindelo et al., 2023). Consequently, this study initially determined the *in vitro* antibacterial activity (Minimum Inhibitory Concentration, MIC) of all synthesized compounds against the following strains via the broth microdilution method: Gram-positive bacteria: *Staphylococcus aureus* ATCC 29213, *S. aureus* ATCC 43300, Methicillin-resistant *S. aureus* MRSA2; Gram-negative bacteria: *Escherichia coli* ATCC 25922 and *Salmonella enterica* subsp*. enterica* SM012, with the results summarized in Table 1. Among the tested compounds, all were ineffective against the Gram-negative strains; notably, compound C12 (MIC = 8 μg/mL) exhibited inhibitory activity against all tested *S. aureus* strains, while other salicylaldehyde derivatives (**C13-C15**) also demonstrated anti-Gram-positive activity capable of inhibiting all tested *S. aureus* strains, whereas the remaining compounds showed weaker antibacterial activity, potentially attributable to their overall poor solubility. The distinct activity profile observed among the synthesized derivatives reveals a clear structure-activity relationship. The emergence of antibacterial activity specifically in the salicylaldehyde-derived compounds (**C12–C15**) strongly indicates that the ortho-hydroxy substituent on the aromatic aldehyde moiety is a critical pharmacophore for anti-staphylococcal activity. This functional group is known to enhance biological activity through mechanisms such as facilitating intramolecular hydrogen bonding (improving stability) or enabling metal chelation. The superior potency of **C12**, compared to **C13-C15**, suggests that its unique substituent (R group) optimally balances molecular properties like lipophilicity and steric bulk, allowing for more effective interaction with the bacterial target. Conversely, the inactivity of compounds **C1**-**C11** and **C16** against all tested strains implies that their respective aldehyde components lack the essential structural features required for effective antibacterial action, which may be linked to inadequate target binding or poor cellular penetration. Furthermore, the consistent inactivity against Gram-negative bacteria across the entire series underscores a common limitation, likely attributable to the impermeable outer membrane of these bacteria, which prevents the compounds from reaching their intracellular target site. We subsequently evaluated **C12** against additional Gram-positive strains, including clinical isolates LN38 and LN51, as well as *Bacillus subtilis* ATCC 6633, *Bacillus cereus* CMCC 63303, *Listeria monocytogenes* CICC 21662, and *Enterococcus faecalis* ATCC 29212; as shown in Table 2, C12 maintained good antibacterial activity against these diverse strains.

**TABLE 1:** MIC^a^ (μM) of Schiff base derivatives.
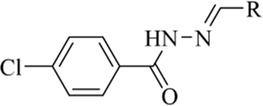

Compounds	R	*E. coli* ATCC 25922	*S. enteritidis* SM012	*S. aureus* ATCC 29213	*S. aureus* ATCC 43300	*S. aureus* MRSA2
Van^b^	-	-	-	0.69	0.69	0.69
Enr^c^	-	0.17	0.17	-	-	-
**C1**	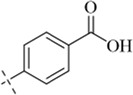	>848	>848	>848	>848	>848
**C2**	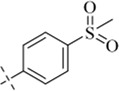	>762	>762	>762	>762	>762
**C3**	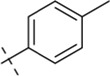	>941	>941	>941	>941	>941
**C4**	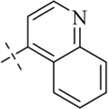	>828	>828	>828	>828	>828
**C5**	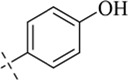	>934	>934	>934	>934	>934
**C6**	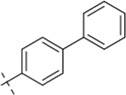	>766	>766	>766	>766	>766
**C7**	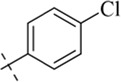	>877	>877	>877	>877	>877
**C8**	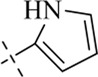	>1,036	>1,036	>1,036	>1,036	>1,036
**C9**	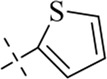	>970	>970	>970	>970	>970
**C10**	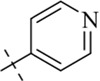	>256	>256	128	128	256
**C11**	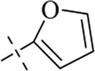	>988	>988	494	494	988
**C12**	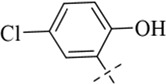	>831	>831	13	13	26
**C13**	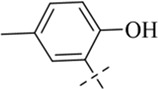	>889	>889	222	222	222
**C14**	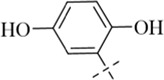	>883	>883	110	110	220
**C15**	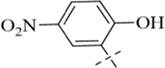	803	803	201	201	201
**C16**	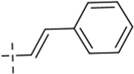	>901	>901	>901	>901	>901

^a^ The minimum inhibitory concentration (MIC) was defined as the lowest concentration preventing visible growth after 24 h (all experiments were performed in triplicate).

^b^ Van = vancomycin is a clinical drug against Gram-positive bacteria.

^c^ Enr = Enrofloxacin is a broad-spectrum quinolone-based antibiotic.

**TABLE 2:** The antibacterial activity of **C12**.

Compounds	*B. cereus* CMCC63303	*L. monocytogenes* CICC21662	*E*. *faecalis* ATCC29212	*S. aureus* LN38	*S. aureus* LN51
Van	1.38	2.76	1.38	1.38	1.38
**C12**	26	13	13	26	13

### 2.3 The toxicity and hemolytic Activity of C12

**FIGURE 2:**
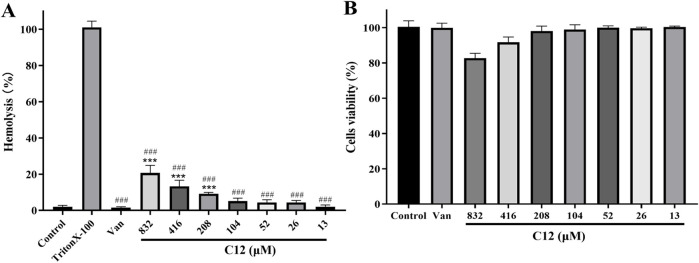
**(A)** Percentage of hemolysis of rabbit blood cells at various C12 concentrations. **(B)** Cytotoxicity of compound C12 against Vero cells after 24 h. Difference is considered significant at ^*^p < 0.05, ^**^p < 0.01, ^***^p < 0.001. Compared with the control group; ^#^p < 0.05, ^##^p < 0.01, ^###^p < 0.001 vs. Triton X-100 group. Data are presented as means ± SEM from three independent experiments. Vancomycin (Van, 176 μM) was used as a reference drug.

To systematically evaluate the biosafety of the lead compound, this study first conducted a comprehensive assessment of the hemolytic activity of compound C12, which exhibited significant *in vitro* antibacterial potency (Greco., 2020; Kindrachuk et al., 2011). In the standard hemolysis assay, 1% Triton X-100 solution and sterile PBS buffer were used as positive and negative controls, respectively. As shown in Figure 2A, compound **C12** did not induce any visible hemolysis across a broad concentration range (13–832 μM), indicating no significant disruptive effect on the membrane integrity of rabbit red blood cells. Notably, its effective antibacterial concentration (≤104 μM) was substantially lower than any concentration observed to initiate hemolysis, demonstrating excellent antibacterial specificity and favorable hemocompatibility. Building upon these findings, and to further investigate the potential toxicity of **C12** towards mammalian cells, this study employed African green monkey kidney epithelial cells (VERO) as a normal cell model. The impact of the compound on cellular metabolic activity was determined using the CCK-8 assay. Results presented in Figure 2B showed that even after 24-h treatment at a high concentration of 832 μM, cells in the **C12**-treated group maintained normal metabolic levels, with no statistically significant metabolic inhibition compared to the negative control group. In conclusion, while demonstrating specific antibacterial activity, compound **C12** exhibited no significant toxicity towards mammalian cells or blood components. These results preliminarily confirm its favorable biosafety profile and development potential as an antibacterial candidate, providing crucial toxicological evidence for subsequent *in vivo* pharmacodynamic evaluation and structural optimization.

### 2.4 Time-killing curve determinations and drug resistance study

**FIGURE 3:**
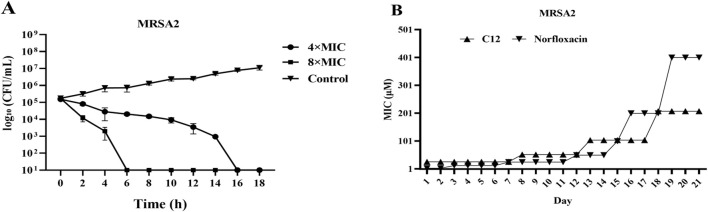
**(A)** Time-kill kinetics of **C12** against MRSA2. **(B)** Resistance development of **C12**. Data are presented as means ± SEM from three independent experiments.

To systematically evaluate the antibacterial properties of compound C12, this study focused on its bactericidal kinetics against Methicillin-resistant *S*. *aureus* MRSA2 and the potential risk of inducing resistance (Bhattacharya et al., 2022). As a common pathogen prone to developing resistance, evaluating the bactericidal efficacy and resistance control of *S. aureus* is crucial for developing novel antimicrobial agents. In the time-kill kinetics assay, bacterial colony counts were determined at various time points to assess the immediate bactericidal effect of **C12**, using dimethyl sulfoxide (DMSO) as a negative control. As shown in Figure 3A, **C12** at 4 × MIC (4 × the minimum inhibitory concentration) completely inhibited the growth of MRSA2 after 16 h of incubation, while at a higher concentration of 8 × MIC, it achieved complete growth inhibition within 6 h. Furthermore, given that Schiff base compounds typically exhibit multi-target mechanisms of action and membrane-disrupting effects, such structures are generally considered less likely to induce bacterial resistance. To further evaluate its capacity to induce resistance, we conducted a serial passage experiment for 21 generations. The results (Figure 3B) demonstrated that the MIC of C12 against MRSA2 increased by no more than 8-fold, accompanied by a low spontaneous resistance frequency, suggesting a potentially high resistance barrier for this compound *in vivo*. In summary, C12 exhibits rapid and potent antibacterial activity at effective concentrations while significantly reducing the probability of resistant mutant development. These findings indicate that this Schiff base derivative, leveraging its multi-mechanistic action, holds considerable promise for development against drug-resistant Gram-positive bacterial infections.

### 2.5 Antimicrobial mechanism investigation

#### 2.5.1 Membrane depolarization and permeabilization assay

**FIGURE 4:**
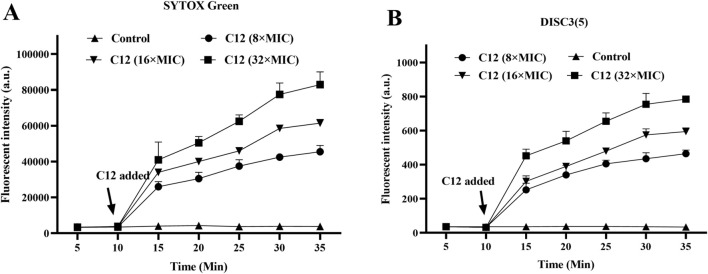
**(A)** Cytoplasmic membrane permeabilization by **C12** assessed using SYTOX Green uptake. **(B)** Cytoplasmic membrane depolarization by **C12** measured with the DiSC35 probe. ^*^p < 0.05, ^**^p < 0.01, ^***^p < 0.001. Data are presented as means ± SEM from three independent experiments. The blank control was bacteria without compound treatment.

Studies have indicated that the antibacterial activity of Schiff base compounds is closely related to their hydrophobic interactions (Liu et al., 2025). Based on the inherent Schiff base group and the significant hydrophobic character in the structure of compound **C12**, we hypothesized that it might exert its antibacterial effect by targeting the bacterial cell membrane, potentially through key processes such as inducing alterations in membrane potential and increasing membrane permeability. To systematically investigate the direct effect of **C12** on the bacterial cell membrane, this study employed the following two fluorescent probes for real-time dynamic monitoring: the membrane potential-sensitive cationic dye 3,3′-Dipropylthiadicarbocyanine iodide (DiSC3(5)) was used to detect changes in bacterial membrane potential, assessing the degree of membrane depolarization; concurrently, the membrane-impermeant nucleic acid stain SYTOX Green, which exhibits enhanced fluorescence upon binding to intracellular nucleic acids, was used to sensitively reflect changes in membrane permeability and integrity, thereby providing a comprehensive evaluation of the membrane-disrupting effects of **C12**. The experimental results showed that within 10 min of adding compound **C12**, a continuous increase in fluorescence intensity was observed in suspensions of *S. aureus* MRSA2 pre-loaded with either the DiSC3(5) or SYTOX Green probe (Figures 4A,B). When the concentration of C12 reached 8 × MIC, the fluorescence intensity of the bacterial suspension system increased significantly by 35 min compared to the initial value, indicating rapid and substantial membrane depolarization and integrity disruption. In contrast, the fluorescence intensity in the blank control group (without **C12**) remained stable throughout the monitoring period, confirming that the experimental conditions themselves did not cause non-specific membrane damage. These results collectively demonstrate that **C12** effectively disrupts the polarized state of the bacterial cell membrane (i.e., alters the distribution of positive and negative charges across the membrane) and significantly increases membrane permeability.

#### 2.5.2 Bacterial cell wall and membrane components modulating C12 Anti-MRSA activity

**FIGURE 5:**
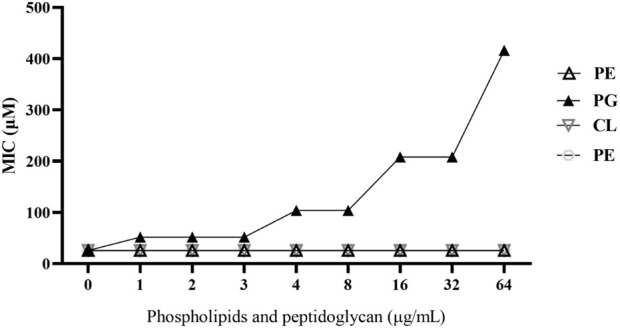
Effects of exogenous addition of peptidoglycan (PGN), phosphatidylglycerol (PG), phosphatidylethanolamine (PE), and cardiolipin (CL) (0–64 μg/mL) on the anti-MRSA2 activity of **C12**, respectively.

To further investigate the interaction of compound **C12** with specific phospholipid components in the bacterial cell membrane—namely phosphatidylethanolamine (PE), phosphatidylglycerol (PG), and cardiolipin (CL)—as well as with the cell wall component peptidoglycan (PGN), we assessed the impact of adding varying concentrations of these molecules to the growth medium on the minimum inhibitory concentration (MIC) of **C12**. As shown in Figure 5, the anti-MRSA2 activity of **C12** was progressively attenuated with increasing concentrations of exogenous PG. When the supplemental PG concentration reached 64 μg/mL, the MIC of **C12** against MRSA2 increased from 26 μM to 416 μM. In contrast, the addition of PE, CL, or PGN had no discernible effect on the MIC of **C12** against MRSA2. This specific interaction was directly corroborated by our SEM/TEM observations, which revealed severe membrane disruption and cell lysis in **C12**-treated bacteria. These results indicate that compound **C12** specifically interacts with phosphatidylglycerol (PG) in the bacterial cell membrane, thereby disrupting its structural integrity.

#### 2.5.3 Determination of reactive oxygen species (ROS) and leakage of proteins and DNA

**FIGURE 6:**
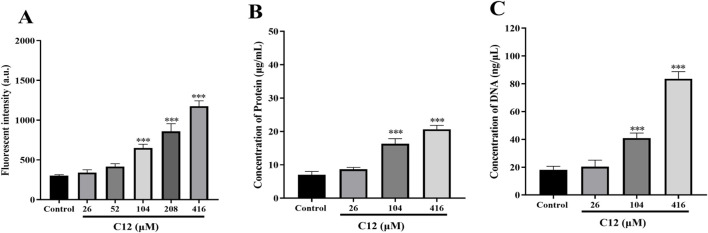
**(A)** Intracellular ROS changes after the treatment of C12 on MRSA2. **(B)** Protein leakage caused by the treatment of C12 on MRSA2. **(C)** DNA leakage resulting from the treatment of C12 on MRSA2. ^*^p < 0.05, ^**^p < 0.01, ^***^p < 0.001. Data are presented as means ± SEM from three independent experiments.

During antibiotic treatment, the disruption of membrane homeostasis often further induces massive accumulation of reactive oxygen species (ROS), which has been recognized as a common bactericidal mechanism for multiple antibiotics. Based on this, we further investigated the effect of **C12** treatment on intracellular ROS levels in bacteria (Xu et al., 2025). We employed the fluorescent probe 2′,7′-dichlorodihydrofluorescein diacetate (DCFH-DA) to detect ROS generation in MRSA2 cells treated with compound **C12**. DCFH-DA itself is virtually non-fluorescent but can be hydrolyzed to DCFH within cells; subsequent intracellular ROS production oxidizes DCFH to the highly fluorescent DCF, whose fluorescence intensity directly reflects the level of intracellular ROS. As shown in Figure 6A, **C12** treatment resulted in a dose-dependent increase in ROS levels within MRSA2 cells. When the **C12** concentration reached 208 μM, ROS production was approximately 3-fold higher than that in the blank control group, indicating that compound **C12** promotes ROS accumulation in MRSA2 cells while disrupting the bacterial cell membrane. Furthermore, we measured the changes in extracellular protein and DNA concentrations after treating MRSA2 with different concentrations of **C12**. The results (Figures 6B,C) revealed that, compared to the blank control, **C12** treatment led to a significant and dose-dependent increase in the leakage of proteins and DNA from the bacterial cells. This demonstrates that **C12** compromises the structural integrity of the MRSA2 cell membrane, resulting in the efflux of intracellular proteins and DNA.

### 2.6 Inhibitory effects towards *S. aureus* biofilm formation

Over 80% of human chronic bacterial infections are associated with biofilm formation. Biofilms are structured bacterial communities encased in a protective extracellular polymeric matrix, which significantly enhances their tolerance to antimicrobials and host immune defenses. These resilient infections, often associated with medical devices, chronic wounds, and cystic fibrosis lungs, are persistent and notoriously difficult to eradicate. This study evaluated the effect of compound **C12** against biofilm formation in *S. aureus* ATCC 43300 and MRSA2 (Table 3). Quantitative analysis using a crystal violet assay demonstrated that **C12** inhibited biofilm formation at 4 × MIC for each strain: 52 µM for *S. aureus* ATCC 43300 and 104 µM for MRSA2. These results confirm the potential of **C12** in effectively preventing staphylococcal biofilm formation.

**TABLE 3:** The inhibitory activity of **C12** against biofilm formation.

Compound	MBIC_90_ (μM)^a^	
	*S. aureus* ATCC 43300	*S. aureus* MRSA2
**C12**	52	104

^*a*^ MBIC_90_ The minimum biofilm inhibitory concentration (MBIC) required for 90% suppression of biofilm formation *in vitro*.

### 2.7 Evaluation of the drug-likeness of C12

To systematically evaluate the drug development potential of the synthesized Schiff base derivative **C12**, key pharmaceutical properties were assessed following the demonstration of its significant anti-Gram-positive activity, low systemic toxicity, unique membrane-targeting mechanism, and anti-biofilm capability. As summarized in Table 4, **C12** exhibits a high human plasma protein binding (PPB) rate of 88.5%, a logD_7.4_ value of 3.47 ± 0.15 indicating moderate lipophilicity, and intermediate metabolic stability in liver microsomes (T_1_/_2_ = 50.72 min; CL = 4.87 μL/min/mg). These properties suggest favorable membrane penetration and acceptable *in vivo* residence time, counterbalanced by potentially limited free drug concentration and aqueous solubility, thus providing clear guidance for subsequent structural optimization and formulation strategies.

**TABLE 4:** Partial drug likeness data for **C12**.

Compound	PPB	logD_7.4_	T_1/2_ (min)	CL (μL/min/mg)
**C12**	88.5%	3.47 ± 0.15	50.72	4.87

### 2.8 Molecular docking

**FIGURE 7:**
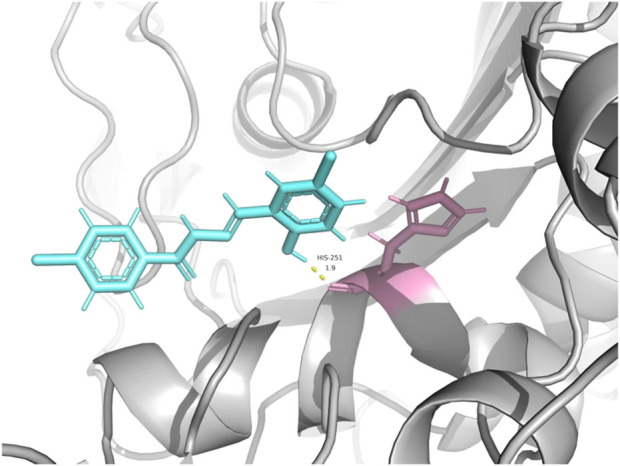
Molecular docking analysis of **C12** to the putative binding site of the target protein (PDB ID: 1VQQ).

To explore the potential molecular target of compound **C12**, we performed molecular docking studies. The PBP2a (PDB ID: 1VQQ), which is closely associated with bacterial membrane integrity, was selected as the receptor (Lang et al., 2025). The results showed that **C12** could stably bind within the active pocket of this protein, with a calculated binding free energy (ΔG) of −5.8 kcal/mol. Specifically, the hydroxyl group of **C12** forms a hydrogen bond with residue His291, while its hydrophobic backbone is accommodated within a hydrophobic pocket (Figure 7). This suggests that **C12** may interfere with the normal function of the protein through competitive inhibition or allosteric effects, thereby contributing to the observed membrane damage, which is consistent with our phenotypic results.

### 2.9 Molecular dynamics simulation analysis

**FIGURE 8:**
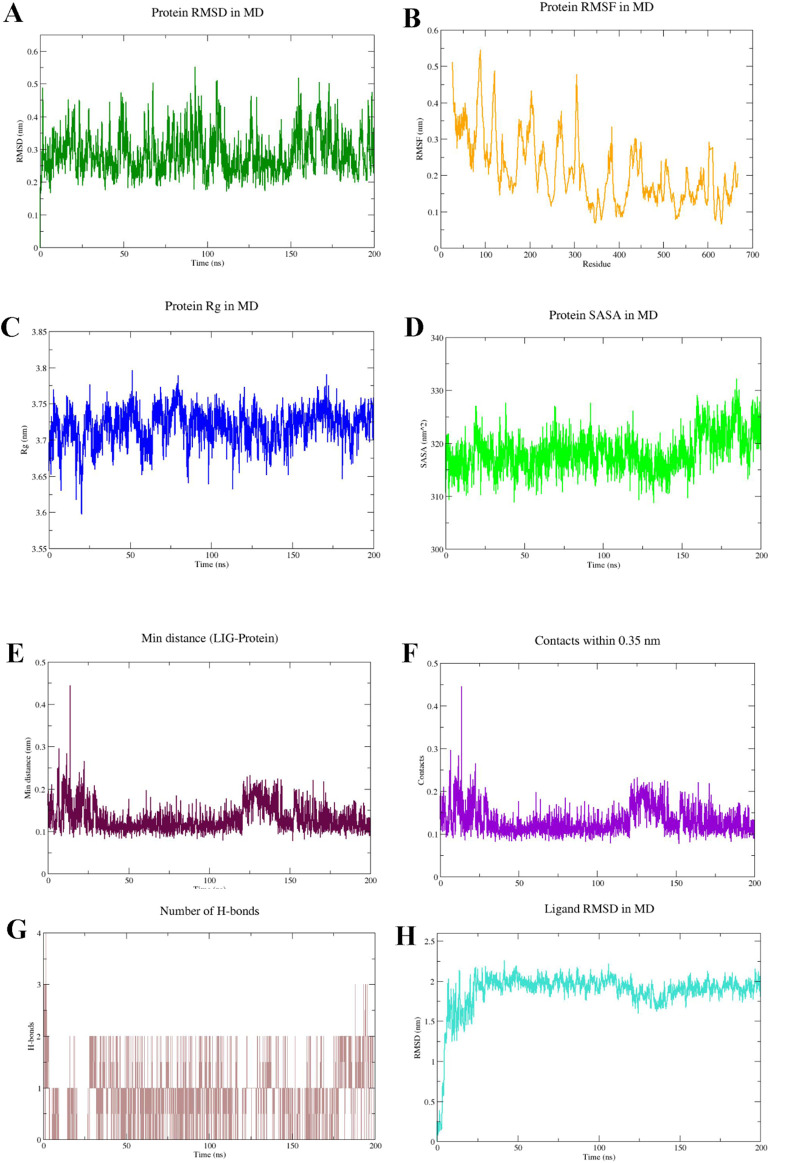
Analysis of protein stability and ligand binding behavior based on 200 ns molecular dynamics simulation. **(A)** Protein backbone RMSD; **(B)** Protein residue RMSF; **(C)** Protein radius of gyration (Rg); **(D)** Protein SASA; **(E)** Minimum distance between ligand and protein; **(F)** Number of ligand–protein contacts within a 0.35 nm threshold; **(G)** Number of ligand–protein hydrogen bonds; **(H)** Ligand RMSD.

During the 200 ns production phase, the protein backbone RMSD entered a stable plateau after an initial relaxation period, exhibiting only minor overall fluctuations (Figure 8A). By the end of the simulation (200 ns), the backbone RMSD was maintained within a range of 0.25–0.37 nm (approximately 0.25 nm at the terminus), indicating that the main chain conformation remained stable throughout the production run. Residue-wise RMSF analysis revealed low fluctuations for most residues, with higher fluctuations concentrated in a few flexible regions (Figure 8B), consistent with the expected local flexibility profile of the protein. Concurrently, the radius of gyration (Rg) of the protein showed no sustained drift during the entire simulation, fluctuating only slightly around its mean value (Figure 8C). The solvent accessible surface area (SASA) also displayed stable oscillations (Figure 8D). The consistent results from RMSD, RMSF, Rg, and SASA collectively support that the overall protein fold and compactness remained stable over the 200 ns timescale, with no signs of significant abnormal collapse or unfolding.

To more directly assess whether ligand “dissociation/diffusion” occurred, we further analyzed geometric and contact-based evidence. The minimum distance between the ligand and protein remained within a low range throughout the simulation, stabilizing at 0.09–0.13 nm (around 0.11 nm) by the end (Figure 8E), indicating the ligand persistently resided in the vicinity of the binding pocket. Consistent with this, the number of ligand-protein contacts within a 0.35 nm threshold remained non-zero for the duration of the simulation (Figure 8F), suggesting the ligand maintained close contact with pocket residues rather than diffusing away.

Hydrogen bond analysis showed that 1–2 hydrogen bond events were observable during the early phase of the simulation. However, this number gradually decreased and approached zero in the later stages (Figure 8G). Notably, while the hydrogen bond count decreased, the corresponding minimum distance and contact number did not show a synchronous decline (Figures 8E,F). This suggests that the system likely underwent a dynamic rearrangement of the binding mode: the initial recognition, primarily driven by hydrogen bonding, gradually evolved into a stable binding state dominated by hydrophobic/van der Waals contacts (and possibly other non-bonded interactions such as aromatic stacking). The ligand RMSD showed considerable variation over time (reaching 1.8–2.1 nm at the terminus; Figure 8H). This metric is sensitive to the choice of reference conformation and fitting procedures and can increase significantly during “in-pocket reorientation/conformational rearrangement.” Therefore, it must be interpreted in conjunction with the minimum distance and contact data. Synthesizing the direct evidence from Figures 8E,F, the ligand maintained stable binding over the 200 ns timescale, accompanied by an evolution of its interaction network from hydrogen-bond-dominated to non-polar-contact-dominated.

## 3 Conclusion

This study identified the lead compound **C12** from a library of 4-chlorobenzohydrazide-based Schiff base derivatives. **C12** exhibited potent and broad-spectrum activity against clinically relevant Gram-positive bacteria, including MRSA, while demonstrating a favorable biosafety profile with no significant cytotoxicity at effective concentrations. Its mechanism of action involves targeting PG in the bacterial membrane, leading to rapid depolarization, increased permeability, and leakage of intracellular contents. This membrane disruption synergistically induces lethal oxidative stress through a sharp increase in intracellular ROS levels. Notably, **C12** also inhibits biofilm formation and shows a low propensity to induce resistance, attributed to this multi-target mechanism. Supported by preliminary drug-likeness data, **C12** emerges as a promising anti-MRSA candidate with a novel action strategy, warranting further investigation for its *in vivo* efficacy and pharmacokinetic optimization.

## 4 Experimental section

### 4.1 Chemically synthetical experiments

All commercially available chemicals and reagents were purchased from Adamas Biochemical Co., Ltd. (Shanghai, China) and were used as received without further purification. Solvents were of analytical grade and were employed directly or dried over activated 4Å molecular sieves when anhydrous conditions were required. Thin-layer chromatography (TLC) analyses were performed on silica gel GF254 pre-coated plates (Yantai Jiangyou Silicone Development Co., Ltd.) to monitor reaction progress. Visualization was achieved under ultraviolet light (254 nm). Nuclear Magnetic Resonance (NMR) spectra, including ^1^H (400 MHz) and ^13^C (100 MHz), were acquired on a Bruker Avance 400 spectrometer at ambient temperature. Chemical shifts (δ) are reported in parts per million (ppm) and are referenced to the residual solvent signals of DMSO-d6 (δH 2.50 ppm, δC 39.5 ppm). High-Resolution Mass Spectrometry (HRMS) data were obtained using an AB Sciex TripleTOF 5,600+ mass spectrometer equipped with an electrospray ionization (ESI) source.

#### 4.1.1 4-{[2-(4-chlorobenzoyl)hydrazineylidene]methyl}benzoic acid (C1)

Ethyl 4-chlorobenzoate (1 mmol) was dissolved in anhydrous ethanol. Hydrazine monohydrate (5 mmol) was then added to the solution, and the mixture was heated under reflux at 80 °C for 8 h. After the reaction was complete, the crude product was isolated and recrystallized from ethanol to afford compound **B**, 4-chlorobenzohydrazide. The product was used directly in the next step without further purification. A mixture of 4-chlorobenzohydrazide (1 mmol) and the appropriate aldehyde derivative (1.05 mmol) was dissolved in anhydrous ethanol. The reaction mixture was heated under reflux at 80 °C for 8 h. Upon completion, the final product C was obtained by recrystallization from ethanol.

250 mg, Yield, 83%. White solid powder. M.P. 285 °C–287 °C. ^1^H NMR (400 MHz, DMSO-d6) δ 12.06 (s, 1H), 8.50 (s, 1H), 7.99 (dd, *J* = 23.8, 8.0 Hz, 4H), 7.85 (d, *J* = 7.8 Hz, 2H), 7.61 (d, *J* = 8.1 Hz, 2H). ^13^C NMR (101 MHz, DMSO-*d*_6_) δ 167.31, 162.65, 147.34, 138.68, 137.17, 132.37, 132.23, 130.20, 130.03, 129.02, 127.56. TOF-MS, m/z: [M + H]^+^, calcd. for C_15_H_12_ClN_2_O_3_^+^, 303.0536, found: 303.0539.

#### 4.1.2 4-chloro-*N'*-[4-(methylsulfonyl)benzylidene]benzohydrazide (C2)

296 mg, Yield, 88%. White solid powder. M.P. 160 °C–162 °C. ^1^H NMR (400 MHz, DMSO-d6) δ 12.14 (s, 1H), 8.53 (s, 1H), 8.10–7.86 (m, 5H), 7.60 (d, *J* = 7.9 Hz, 2H), 3.26 (s, 3H). ^13^C NMR (101 MHz, DMSO-d6) δ 162.56, 146.37, 141.70, 139.25, 137.06, 132.04, 129.86, 128.83, 127.96, 127.73, 43.67. TOF-MS, m/z: [M + H]^+^, calcd. for C_15_H_14_ClN_2_O_3_S^+^, 337.0413, found: 337.0417.

#### 4.1.3 4-chloro-*N*'-(4-methylbenzylidene)benzohydrazide (C3)

248 mg, Yield, 91%. White solid powder. M.P. 173 °C–175 °C. ^1^H NMR (400 MHz, DMSO-d6) δ 11.85 (s, 1H), 8.42 (s, 1H), 7.94 (d, *J* = 8.2 Hz, 2H), 7.61 (t, *J* = 9.1 Hz, 4H), 7.27 (d, *J* = 7.7 Hz, 2H), 2.34 (s, 3H). ^13^C NMR (101 MHz, DMSO-d6) δ 162.40, 148.60, 140.42, 132.61, 131.94, 129.93, 129.86, 128.96, 127.53, 21.44. TOF-MS, m/z: [M + H]^+^, calcd. for C_15_H_14_N_2_O^+^, 273.0794, found: 273.0798.

#### 4.1.4 4-chloro-*N'*-(quinolin-4-ylmethylene)benzohydrazide (C4)

263 mg, Yield, 85%. White solid powder. M.P. 167 °C–169 °C. ^1^H NMR (400 MHz, DMSO-d6) δ 11.80 (s, 1H), 10.83 (s, 1H), 9.06–8.88 (m, 2H), 8.26 (d, *J* = 8.2 Hz, 1H), 7.99 (d, *J* = 8.3 Hz, 2H), 7.76 (d, *J* = 8.0 Hz, 1H), 7.70–7.48 (m, 4H), 6.99 (d, *J* = 8.0 Hz, 1H). ^13^C NMR (101 MHz, DMSO-d6) δ 161.58, 155.66, 148.96, 136.26, 132.18, 131.51, 130.27, 129.28, 128.38, 127.50, 124.88, 124.54, 124.26, 122.50, 120.14, 107.84. TOF-MS, m/z: [M + H]^+^, calcd. for C_17_H_13_ClN_3_O^+^, 310.0747, found: 310.0751.

#### 4.1.5 4-chloro-*N'*-(4-hydroxybenzylidene)benzohydrazid*e* (C5)

246 mg, Yield, 90%. White solid powder. M.P. 205 °C–207 °C. ^1^H NMR (400 MHz, DMSO-d6) δ 11.71 (s, 1H), 9.95 (s, 1H), 8.35 (s, 1H), 7.93 (d, *J* = 8.4 Hz, 2H), 7.58 (t, *J* = 8.0 Hz, 3H), 6.85 (d, *J* = 8.4 Hz, 2H). ^13^C NMR (101 MHz, DMSO-d6) δ 162.04, 159.74, 148.72, 136.62, 132.57, 129.68, 129.15, 128.74, 125.43, 115.96. TOF-MS, m/z: [M + H]^+^, calcd. for C_14_H_12_ClN_2_O_2_^+^, 275.0587, found: 275.0589.

#### 4.1.6 *N'*-[(1,1′-biphenyl)-4-ylmethylene]-4-chlorobenzohydrazide (C6)

296 mg, Yield, 89%. White solid powder. M.P. 185 °C–187 °C. ^1^H NMR (400 MHz, DMSO-d6) δ 11.96 (s, 1H), 8.50 (s, 1H), 8.09–7.29 (m, 12H). ^13^C NMR (101 MHz, DMSO-d6) δ 162.54, 148.18, 142.14, 139.79, 137.08, 133.83, 132.62, 130.05, 129.50, 129.06, 128.37, 128.23, 127.54, 127.15. TOF-MS, m/z: [M + H]^+^, calcd. for C_20_H_16_ClN_2_O^+^, 335.0943, found: 335.0947.

#### 4.1.7 4-chloro-*N'*-(4-chlorobenzylidene)benzohydrazide (C7)

292 mg, Yield, 84%. White solid powder. M.P. 172 °C–174 °C. ^1^H NMR (400 MHz, DMSO-d6) δ 11.98 (s, 1H), 8.44 (s, 1H), 7.94 (d, *J* = 8.1 Hz, 2H), 7.76 (d, *J* = 8.1 Hz, 2H), 7.56 (dd, *J* = 34.8, 8.1 Hz, 4H). ^13^C NMR (101 MHz, DMSO-d6) δ 162.56, 147.23, 137.10, 135.05, 133.60, 132.43, 129.99, 129.37, 129.18, 129.01. TOF-MS, m/z: [M + H]^+^, calcd. for C_14_H_11_Cl_2_N_2_O^+^, 293.0248, found: 293.0251.

#### 4.1.8 *N'*-((1H-pyrrol-2-yl)methylene)-4-chlorobenzohydrazide (C8)

202 mg, Yield, 82%. White solid powder. M.P. 190 °C–192 °C. ^1^H NMR (400 MHz, DMSO-d6) δ 11.59 (d, *J* = 26.8 Hz, 2H), 8.29 (s, 1H), 7.93 (d, *J* = 8.4 Hz, 2H), 7.57 (d, *J* = 8.4 Hz, 2H), 6.93 (s, 1H), 6.50 (s, 1H), 6.14 (s, 1H). ^13^C NMR (101 MHz, DMSO-d6) δ 161.92, 141.44, 136.57, 132.70, 129.69, 129.13, 128.75, 127.26, 122.91, 113.80, 109.59. TOF-MS, m/z: [M + H]^+^, calcd. for C_12_H_11_ClN_3_O^+^, 248.0590, found: 248.0593.

#### 4.1.9 4-Chloro-*N'*-(thiophen-2-ylmethylene)benzohydrazide (C9)

224 mg, Yield, 85%. White solid powder. M.P. 195 °C–197 °C. ^1^H NMR (400 MHz, DMSO-d6) δ 11.88 (s, 1H), 8.67 (s, 1H), 7.93 (d, *J* = 8.3 Hz, 2H), 7.77–7.34 (m, 4H), 7.23–7.02 (m, 1H). ^13^C NMR (101 MHz, DMSO-d6) δ 161.74, 143.05, 138.80, 136.39, 131.87, 130.86, 129.28, 128.86, 128.35, 127.64. TOF-MS, m/z: [M + H]^+^, calcd. for C_12_H_10_ClN_2_OS^+^, 265.0202, found: 265.0206.

#### 4.1.10 4-Chloro-*N'*-(pyridin-4-ylmethylene)benzohydrazide (C10)

222 mg, Yield, 86%. White solid powder. M.P. 199 °C–201 °C. ^1^H NMR (400 MHz, DMSO-d6) δ 12.18 (s, 1H), 8.64 (s, 2H), 8.43 (s, 1H), 7.95 (d, *J* = 7.9 Hz, 2H), 7.63 (dd, *J* = 26.1, 5.9 Hz, 4H). ^13^C NMR (101 MHz, DMSO-d6) δ 162.89, 151.53, 150.70, 146.16, 141.85, 137.39, 132.19, 130.13, 129.09, 122.58, 121.49. TOF-MS, m/z: [M + H]^+^, calcd. for C_13_H_11_ClN_3_OS^+^, 260.0590, found: 260.0592.

#### 4.1.11 4-chloro-*N'*-(furan-2-ylmethylene)benzohydrazide (C11)

205 mg, Yield, 83%. White solid powder. M.P. 238 °C–240 °C. ^1^H NMR (400 MHz, DMSO-d6) δ 11.86 (s, 1H), 8.35 (s, 1H), 8.05–7.73 (m, 3H), 7.59 (d, *J* = 8.3 Hz, 2H), 6.94 (d, *J* = 3.1 Hz, 1H), 6.63 (s, 1H). ^13^C NMR (101 MHz, DMSO-d6) δ 161.81, 149.16, 145.05, 137.68, 136.41, 131.83, 129.30, 128.36, 113.49, 112.00. TOF-MS, m/z: [M + H]^+^, calcd. for C_12_H_10_ClN_2_O_2_^+^, 249.0431, found: 249.0435.

#### 4.1.12 4-chloro-*N*'-(5-chloro-2-hydroxybenzylidene)benzohydrazide (C12)

262 mg, Yield, 85%. White solid powder. M.P. 188 °C–190 °C. ^1^H NMR (400 MHz, DMSO-d6) δ 12.23 (s, 1H), 11.24 (s, 1H), 8.62 (s, 1H), 7.96 (d, *J* = 8.3 Hz, 2H), 7.75–7.53 (m, 3H), 7.30 (d, *J* = 8.7 Hz, 1H), 6.95 (d, *J* = 8.7 Hz, 1H). ^13^C NMR (101 MHz, DMSO-d6) δ 161.71, 155.85, 145.90, 136.70, 131.24, 130.62, 129.39, 128.42, 127.38, 122.81, 120.44, 118.02. TOF-MS, m/z: [M + H]^+^, calcd. for C_14_H_11_Cl_2_N_2_O_2_^+^, 309.0197, found: 309.0201.

#### 4.1.13 4-chloro-*N*'-(2-hydroxy-5-methylbenzylidene)benzohydrazide (C13)

253 mg, Yield, 88%. White solid powder. M.P. 185 °C–187 °C. ^1^H NMR (400 MHz, DMSO-d6) δ 12.14 (s, 1H), 10.98 (s, 1H), 8.60 (s, 1H), 7.97 (d, *J* = 8.4 Hz, 2H), 7.61 (d, *J* = 8.4 Hz, 2H), 7.35 (s, 1H), 7.10 (d, *J* = 7.7 Hz, 1H), 6.83 (d, *J* = 8.3 Hz, 1H), 2.24 (s, 3H). ^13^C NMR (101 MHz, DMSO-d6) δ 161.97, 155.56, 148.68, 137.01, 132.38, 131.79, 129.77, 129.50, 128.84, 128.13, 118.53, 116.49, 20.13. TOF-MS, m/z: [M + H]^+^, calcd. for C_15_H_14_ClN_2_O_2_^+^, 289.0744, found: 289.0747.

#### 4.1.14 4-chloro-*N*'-(2,5-dihydroxybenzylidene)benzohydrazide (C14)

247 mg, Yield, 85%. White solid powder. M.P. 214 °C–216 °C. ^1^H NMR (400 MHz, DMSO-d6) δ 12.05 (s, 1H), 10.36 (s, 1H), 9.00 (s, 1H), 8.59 (s, 1H), 7.96 (d, *J* = 8.3 Hz, 2H), 7.60 (d, *J* = 8.3 Hz, 2H), 7.00 (s, 1H), 6.76 (s, 2H). ^13^C NMR (101 MHz, DMSO-d6) δ 162.01, 150.53, 150.15, 148.20, 137.01, 131.94, 129.79, 128.86, 119.33, 117.35, 114.06. TOF-MS, m/z: [M + H]^+^, calcd. for C_14_H_12_ClN_2_O_3_^+^, 291.0536, found: 291.0539.

#### 4.1.15 4-chloro-*N*'-(2-hydroxy-5-nitrobenzylidene)benzohydrazide (C15)

274 mg, Yield, 86%. White solid powder. M.P. 212 °C–214 °C. ^1^H NMR (400 MHz, DMSO-d6) δ 12.34 (s, 1H), 8.67 (d, *J* = 67.7 Hz, 1H), 8.08 (d, *J* = 91.2 Hz, 2H), 7.61 (s, 1H), 7.13 (s, 1H). ^13^C NMR (101 MHz, DMSO-d6) δ 161.94, 161.42, 143.91, 139.33, 136.31, 130.79, 129.01, 128.04, 126.02, 123.06, 119.37, 116.50. TOF-MS, m/z: [M + H]^+^, calcd. for C_14_H_11_ClN_3_O_4_^+^, 320.0438, found: 320.0441.

#### 4.1.16 4-chloro-*N*'-(3-phenylallylidene)benzohydrazide (C16)

244 mg, Yield, 86%. White solid powder. M.P. 174 °C–176 °C. ^1^H NMR (400 MHz, DMSO-d6) δ 11.80 (s, 1H), 8.25 (s, 1H), 7.93 (d, *J* = 8.2 Hz, 2H), 7.61 (t, *J* = 9.3 Hz, 4H), 7.36 (dd, *J* = 19.0, 7.2 Hz, 3H), 7.07 (d, *J* = 3.6 Hz, 2H). ^13^C NMR (101 MHz, DMSO-d6) δ 161.50, 149.68, 138.86, 136.13, 135.45, 131.69, 129.12, 128.39, 128.12, 126.69, 125.15. TOF-MS, m/z: [M + H]^+^, calcd. for C_16_H_14_ClN_2_O^+^, 285.0794, found: 285.0796.

### 4.2 Determination of minimum inhibitory concentration

For detailed procedures, refer to the Supplementary Material (Chung et al., 2021; Sindelo et al., 2023).

### 4.3 Time-killing kinetics

For detailed procedures, refer to the Supplementary Material (Kong et al., 2023).

### 4.4 Drug resistance study

For detailed procedures, refer to the Supplementary Material (Kong et al., 2023).

### 4.5 Hemolysis assay

For detailed procedures, refer to the Supplementary Material (Tian et al., 2025).

### 4.6 Cytotoxicity assay

For detailed procedures, refer to the Supplementary Material (Tian et al., 2025).

### 4.7 Biofilm inhibition assay

For detailed procedures, refer to the Supplementary Material (Liu et al., 2025).

### 4.8 Membrane depolarization study

For detailed procedures, refer to the Supplementary Material (Kong et al., 2023; Xu et al., 2025).

### 4.9 Interaction of C12 with PEG and cell membrane phospholipids

For detailed procedures, refer to the Supplementary Material (Xu et al., 2025; Tian et al., 2025).

### 4.10 DNA and protein leakage

For detailed procedures, refer to the Supplementary Material (Xu et al., 2025; Tian et al., 2025).

### 4.11 ROS detection assay

For detailed procedures, refer to the Supplementary Material (Xu et al., 2025; Tian et al., 2025).

### 4.12 Plasma protein binding rate of C12

For detailed procedures, refer to the Supplementary Material (Ueda et al., 2025; Wu et al., 2012).

### 4.13 Determination of logD_7.4_ for C12

For detailed procedures, refer to the Supplementary Material (Andrés et al., 2015; Slavik et al., 2015).

### 4.14 Liver microsomal stability assay for C12

For detailed procedures, refer to the Supplementary Material (Knights et al., 2016; Liu et al., 2020).

### 4.15 Molecular docking

For detailed procedures, refer to the Supplementary Material (Lang et al., 2025).

### 4.16 Molecular dynamics

For detailed procedures, refer to the Supplementary Material (Fang et al., 2020).

### 4.17 Statistical analysis

Data are presented as the mean ± SEM from at least three independent experiments. Statistical significance was assessed by one-way analysis of variance (ANOVA) using SPSS software (version 21.0).

## Data Availability

The datasets generated and/or analyzed during the current study are available in the Figshare repository, DOI: 10.6084/m9.figshare.31369891.
