# Supplementary material for: Membrane-targeted schiff base derivatives overcome MRSA resistance through phosphatidylglycerol binding and ROS-mediated killing
Source: Front Chem. 2026 Mar 4;14:1753350. doi: 10.3389/fchem.2026.1753350 (PMC12996145; doi:10.3389/fchem.2026.1753350)
Supplement: Supplementary file 1 [file DataSheet1.doc]

**Membrane-Targeted Schiff Base Derivatives Overcome MRSA Resistance through Phosphatidylglycerol Binding and ROS-Mediated Killing**

Yaguang Liu^1*^, Lianzhi Hu[[1]](#footnote-2), Binbin Liu^1^, Zheng Qu^1^

^1^*The Second Hospital of QinHuangDao, Pharmacy Department,* *QinHuangDao, China, 066000*

**Table of Contents**

**Spectral Data**······························································································1-25

**Determination of Minimum Inhibitory Concentration**···································26

**Time-Killing Kinetics**····················································································26

**Drug Resistance Study**··················································································26

**Hemolysis Assay** ···························································································27

**Cytotoxicity Assay** ························································································27

**Biofilm Inhibition Assay**················································································27

**Membrane Depolarization Study** ··································································28

**Interaction of C12 with PEG and Cell Membrane Phospholipids**···················28

**DNA and Protein Leakage** ············································································28

**ROS Detection Assay**··················································································29

**Plasma Protein Binding Rate of C12**·······························································29

**Determination of logD_7.4_ for C12**·····································································29

**Liver Microsomal Stability Assay for C12**·······················································29

**Molecular Docking**························································································30

**Molecular Dynamics Simulations**··································································30


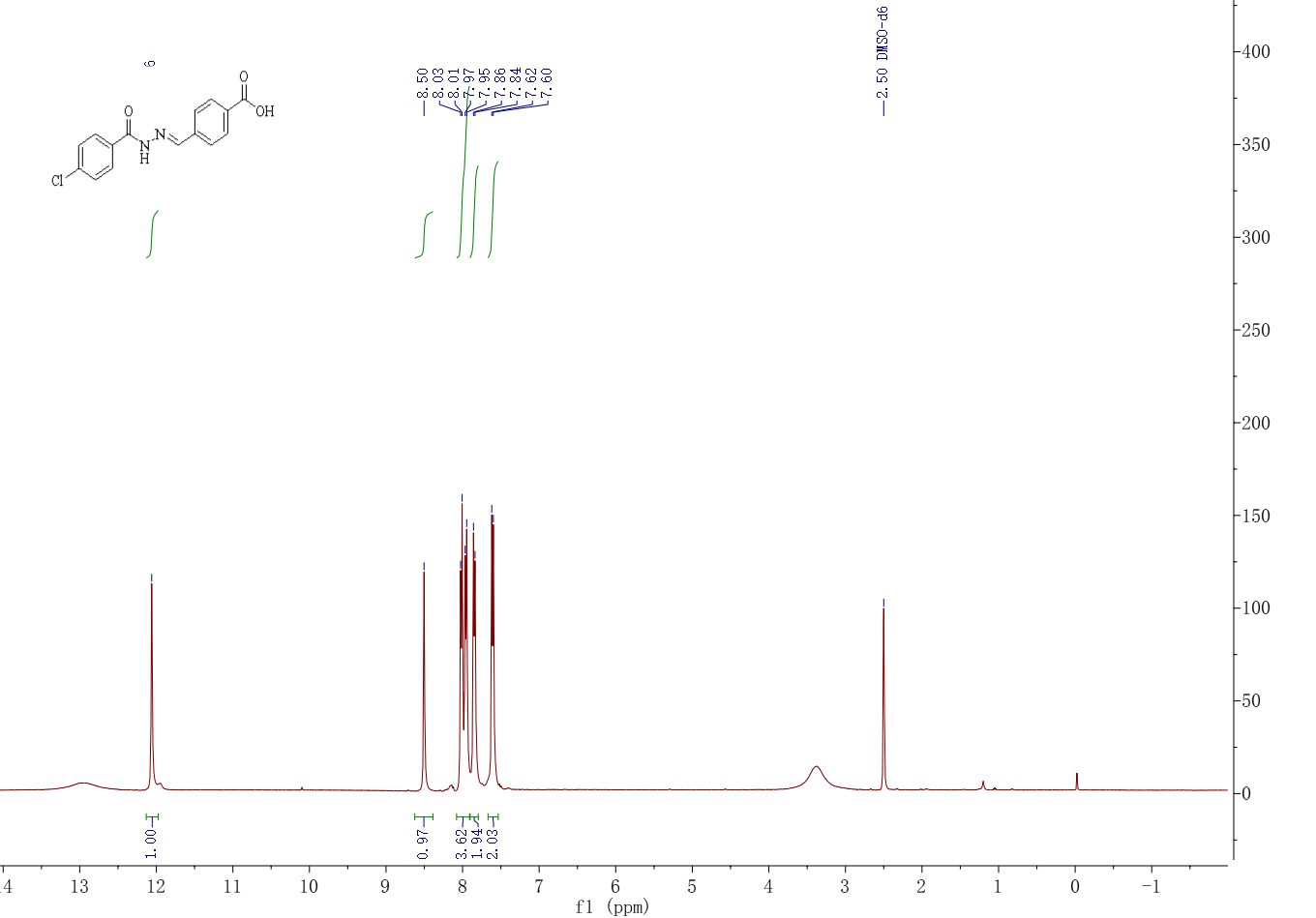


Fig 1. *^1^H NMR of* **C1** (400 MHz, DMSO)


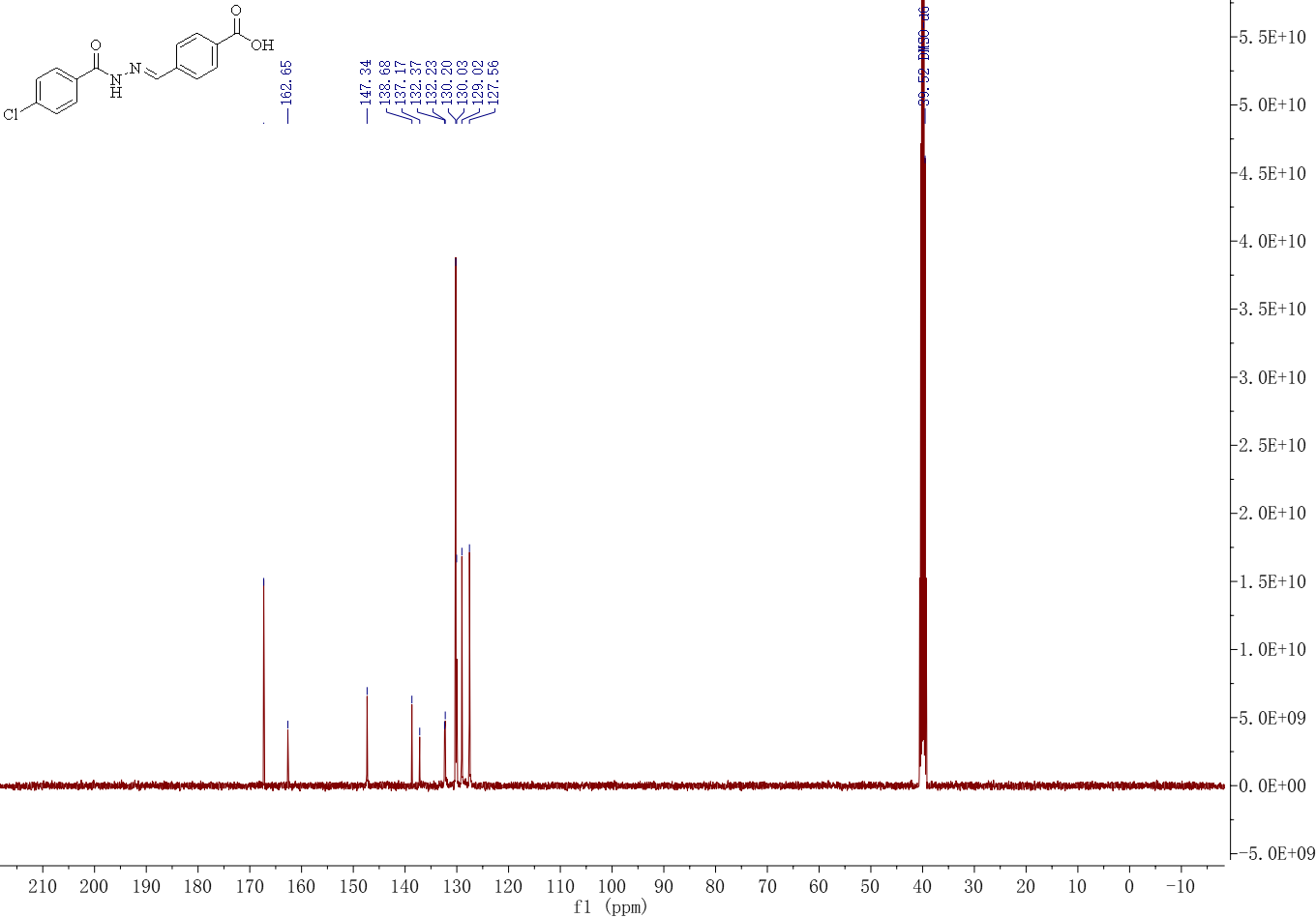


Fig 2. *^13^C NMR of* **C1** (100 MHz, DMSO)


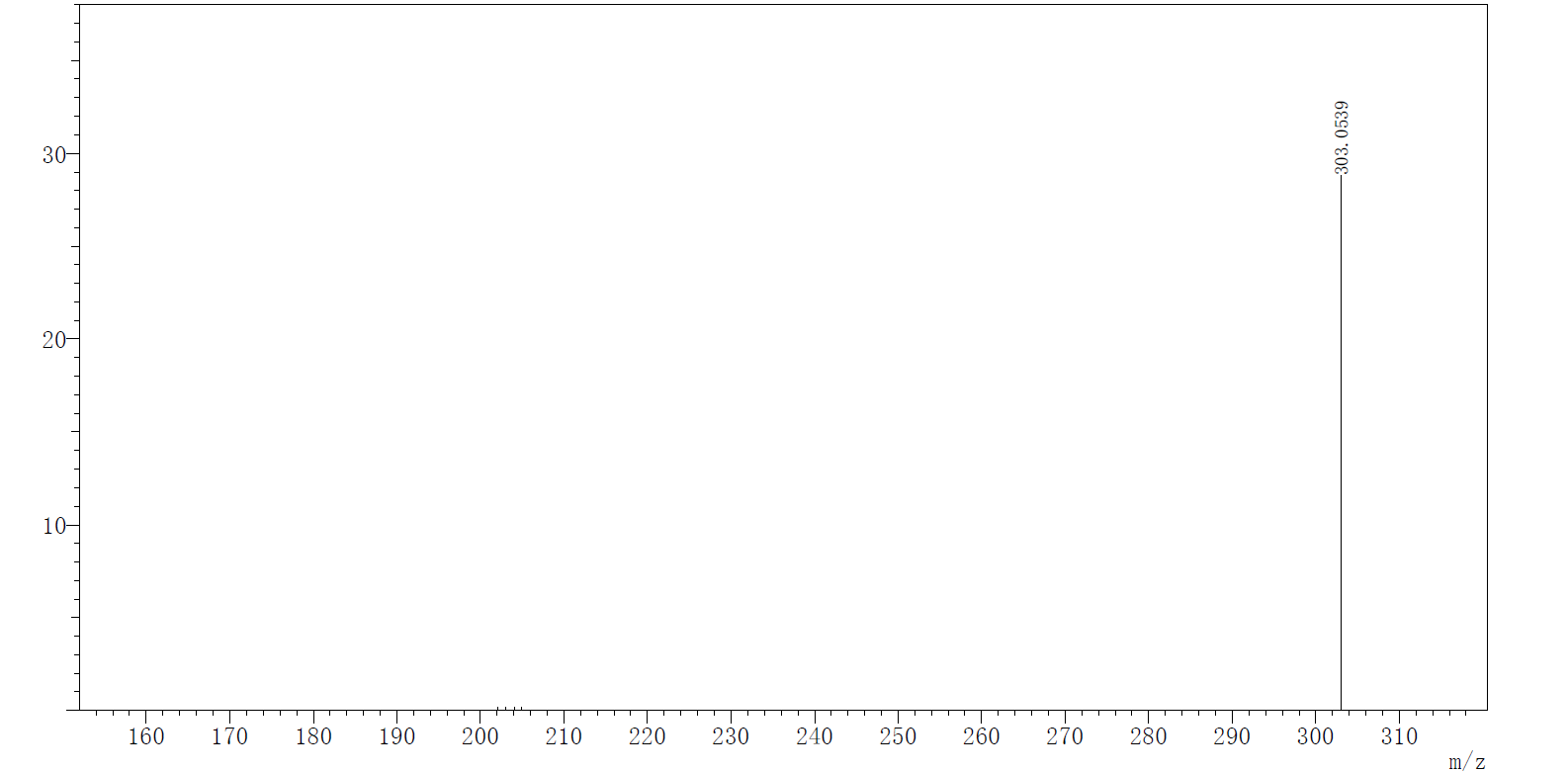


Fig 3. Mass spectrum of compound **C1**


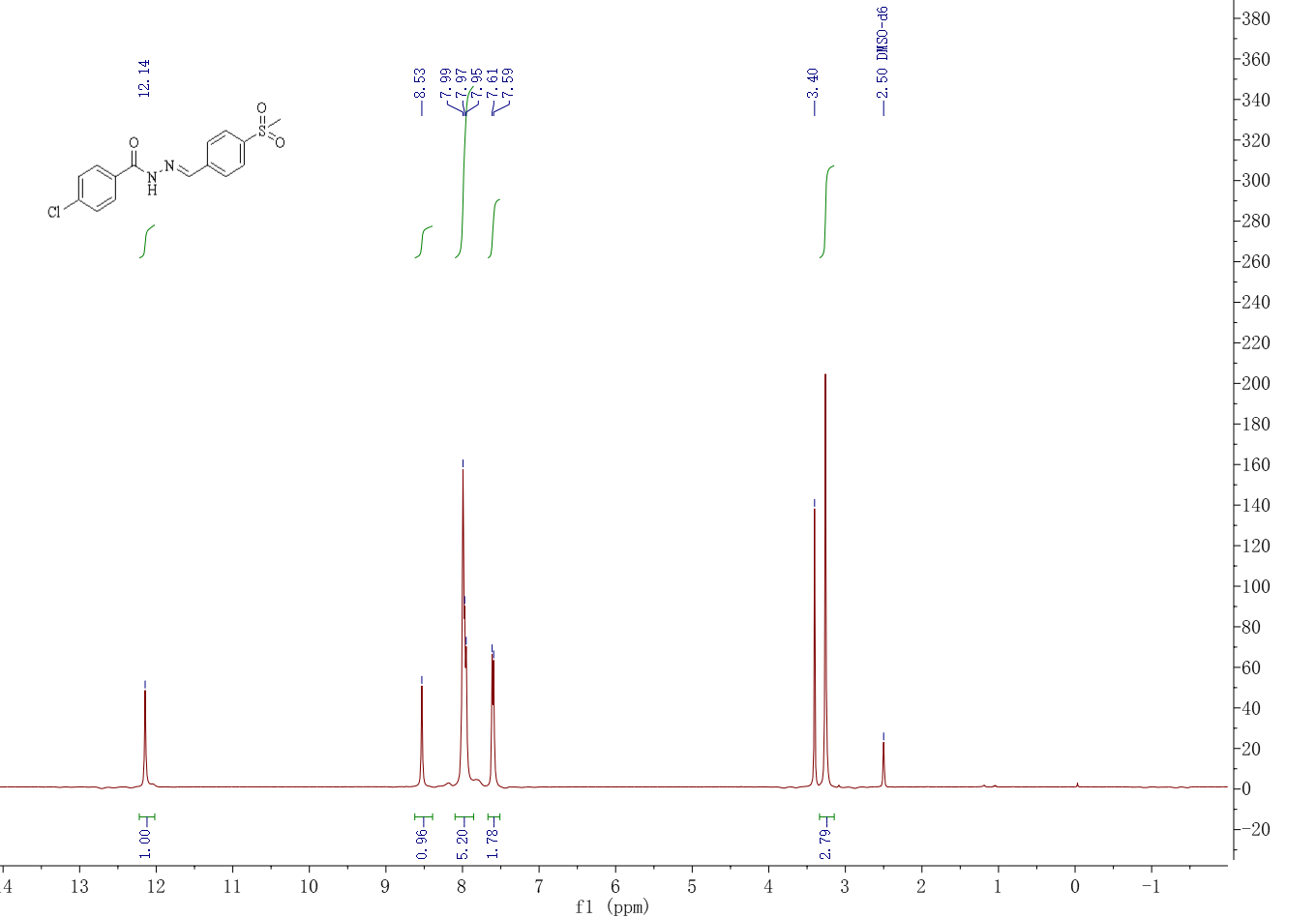


Fig 4. *^1^H NMR of* **C2** (400 MHz, DMSO)


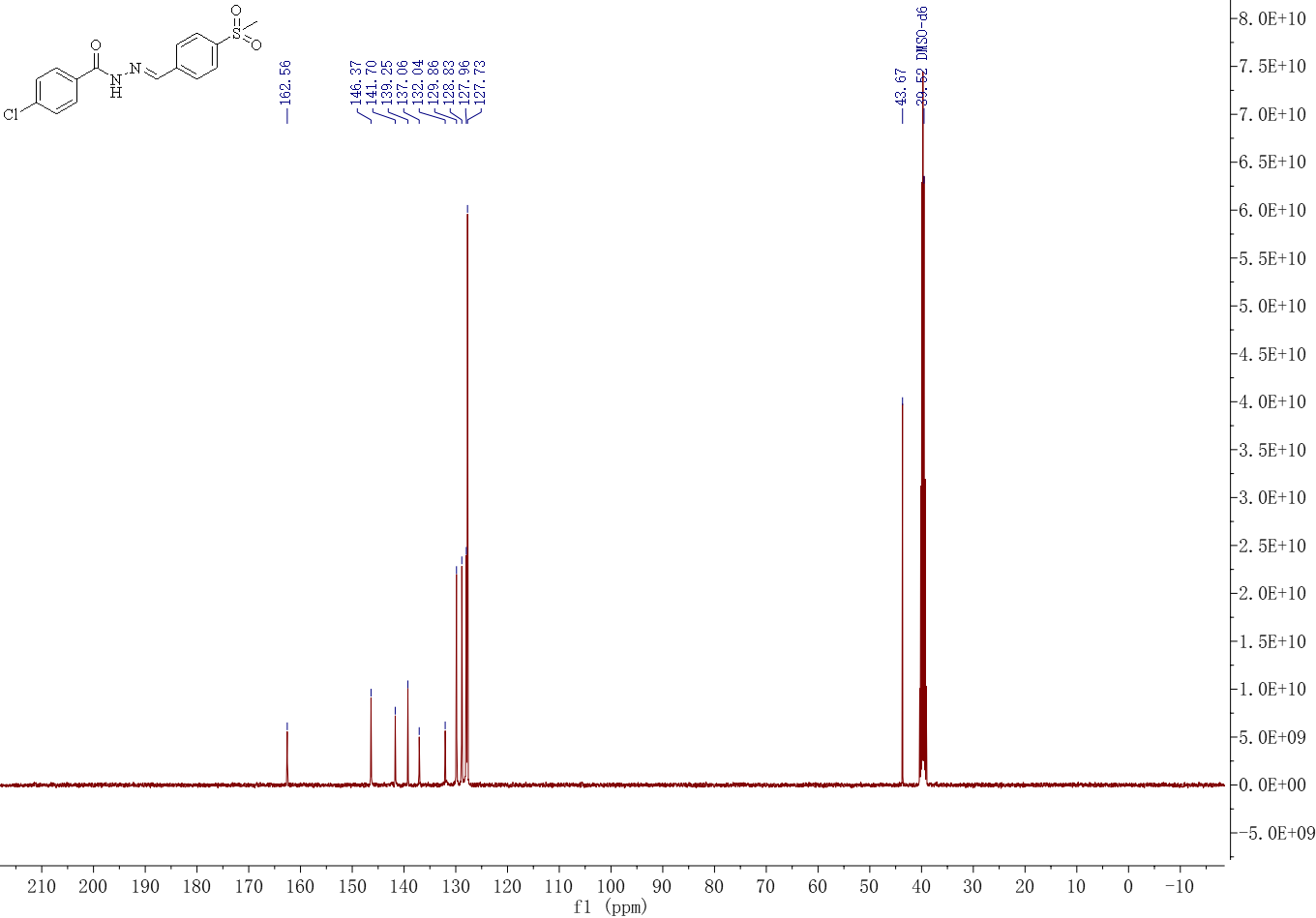


Fig 5. *^13^C NMR of* **C2** (100 MHz, DMSO)


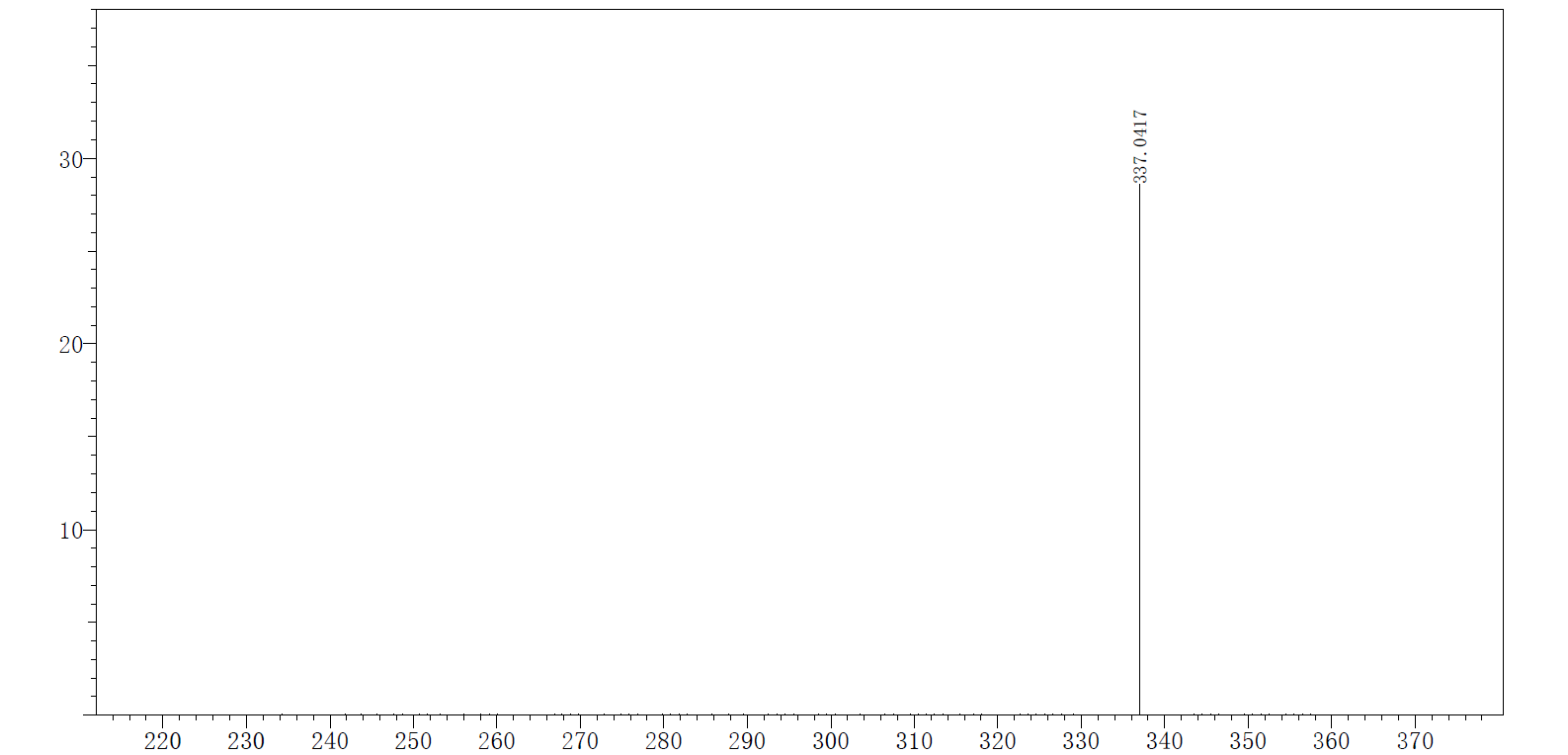


Fig 6. Mass spectrum of compound **C2**


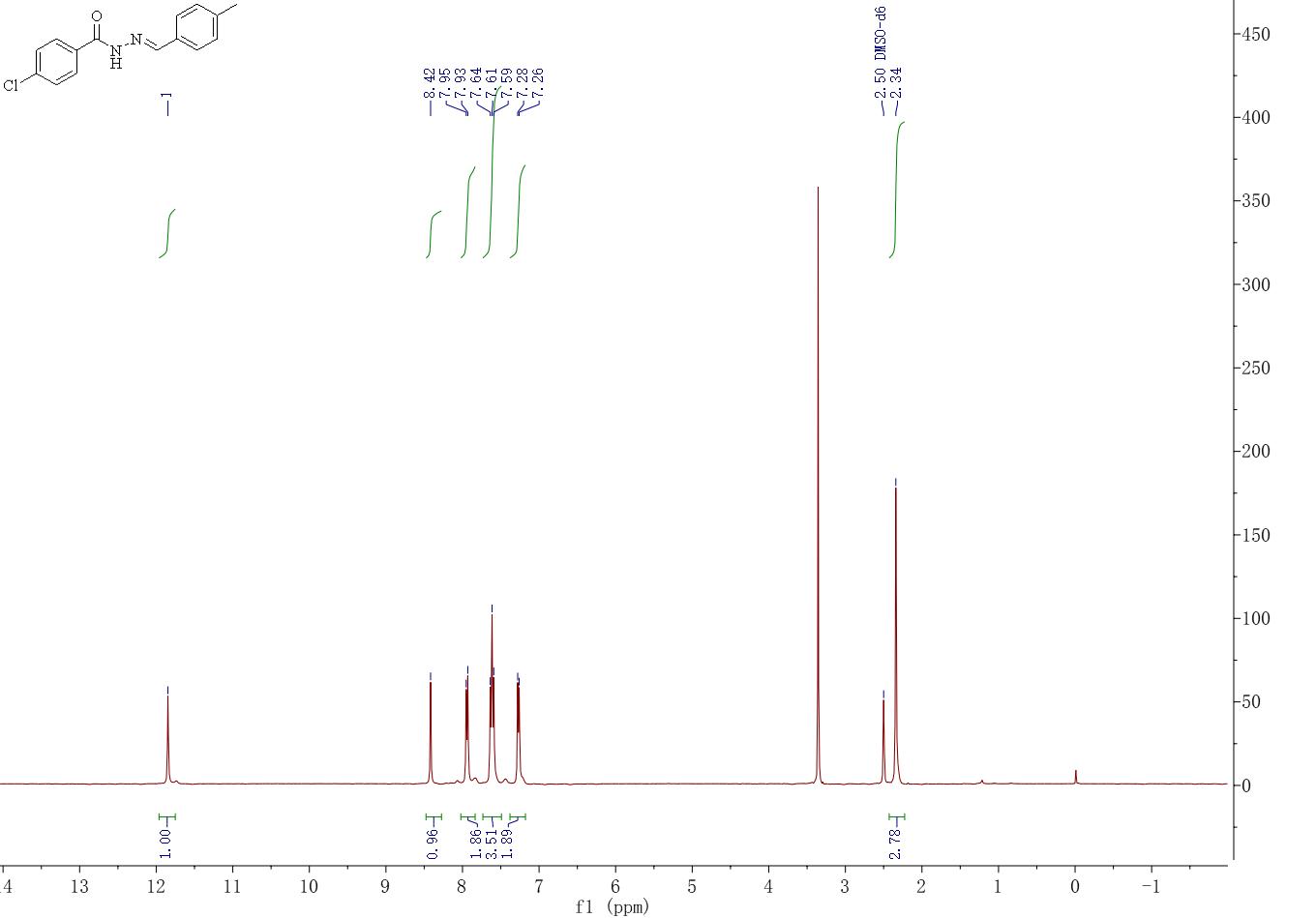


Fig 7. *^1^H NMR of* **C3** (400 MHz, DMSO)


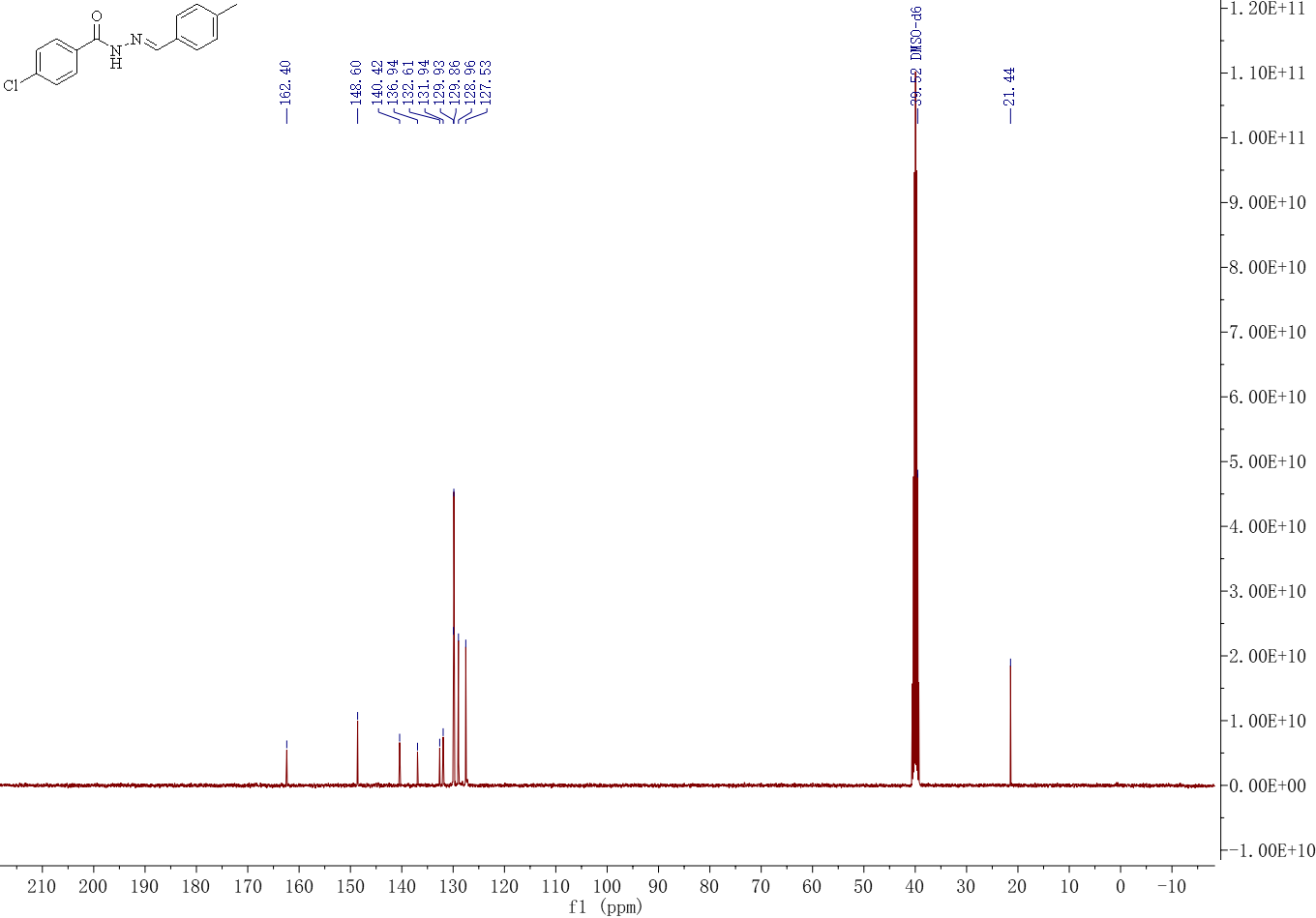


Fig 8. *^13^C NMR of* **C3** (100 MHz, DMSO)


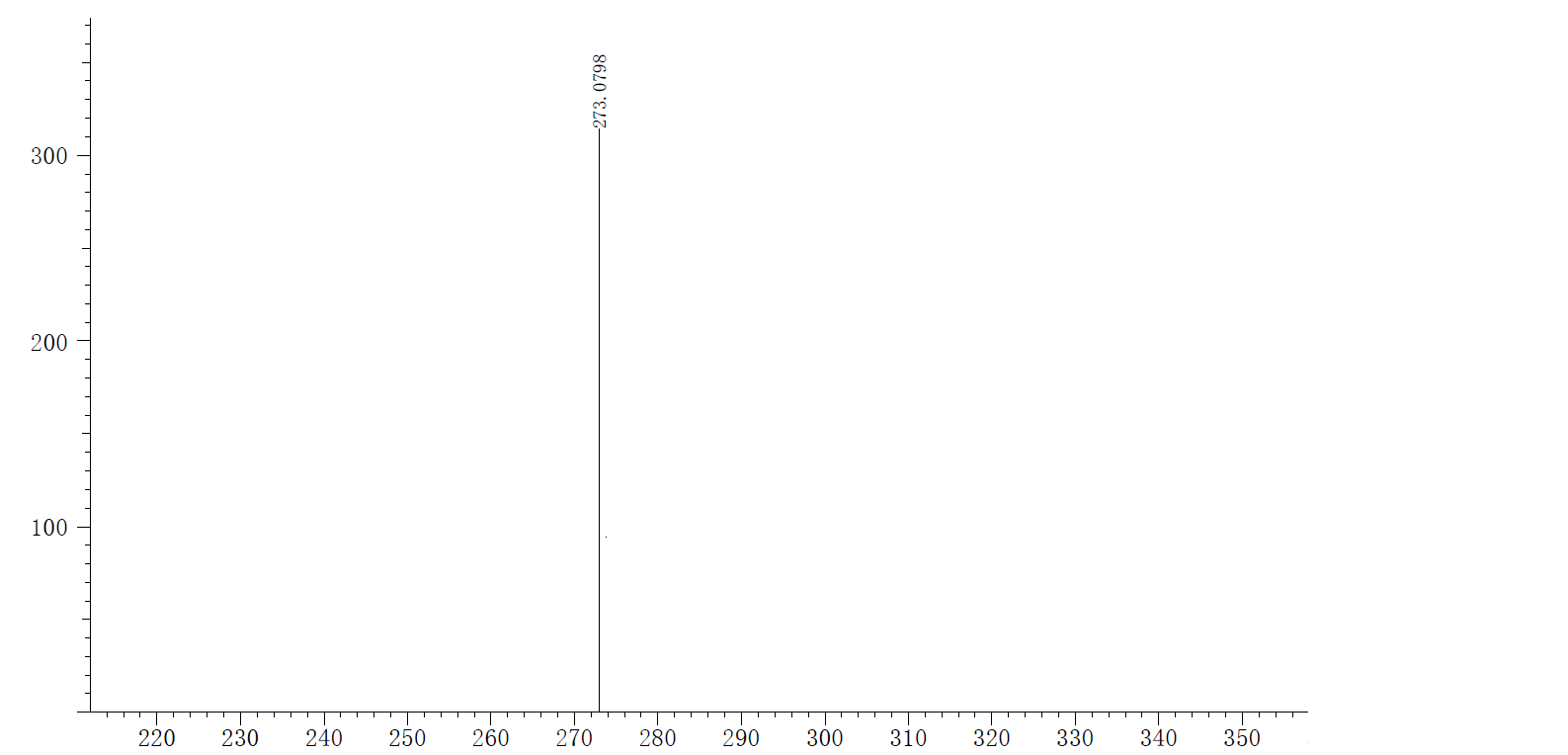


Fig 9. Mass spectrum of compound **C3**


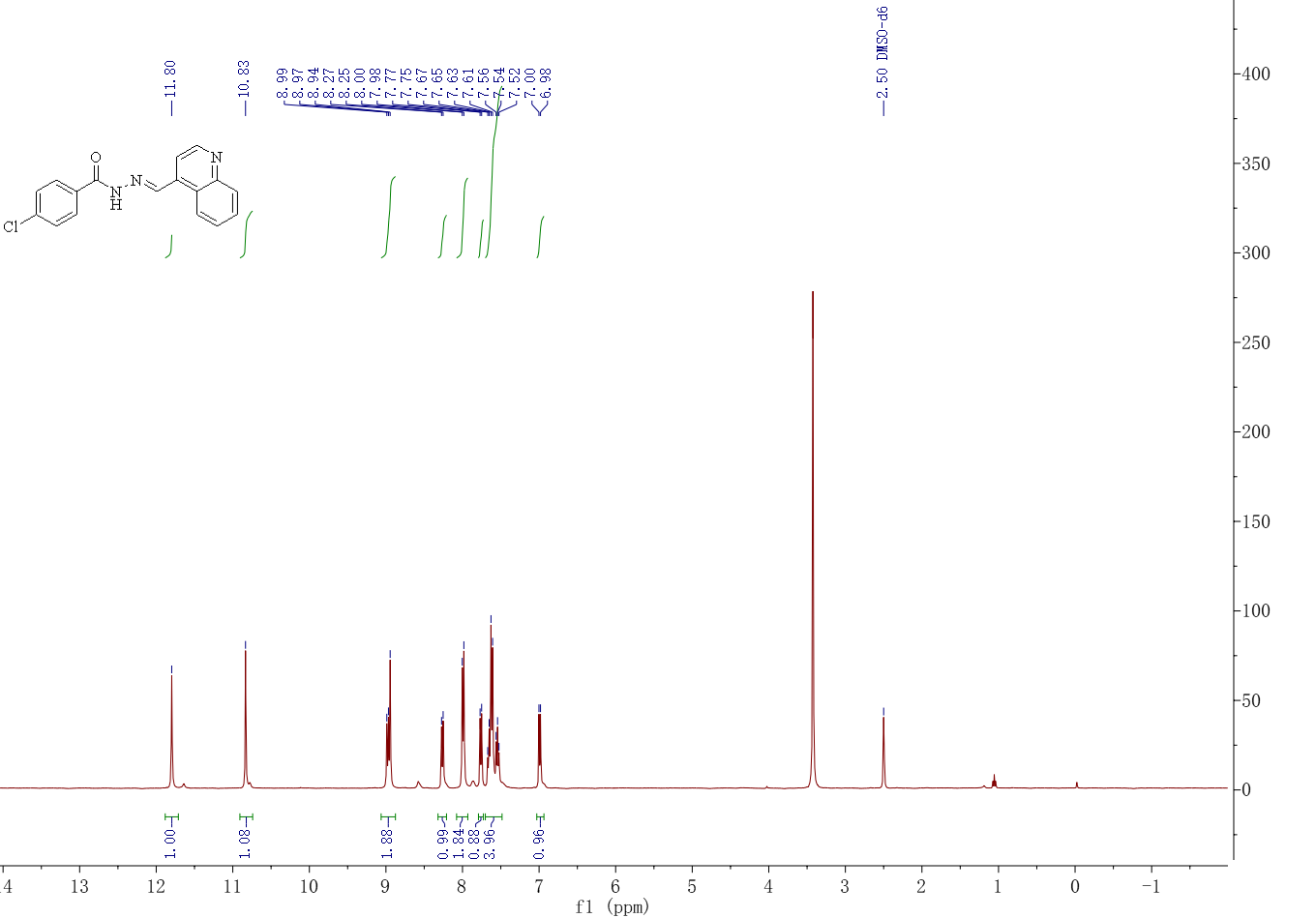


Fig 10. *^1^H NMR of* **C4** (400 MHz, DMSO)


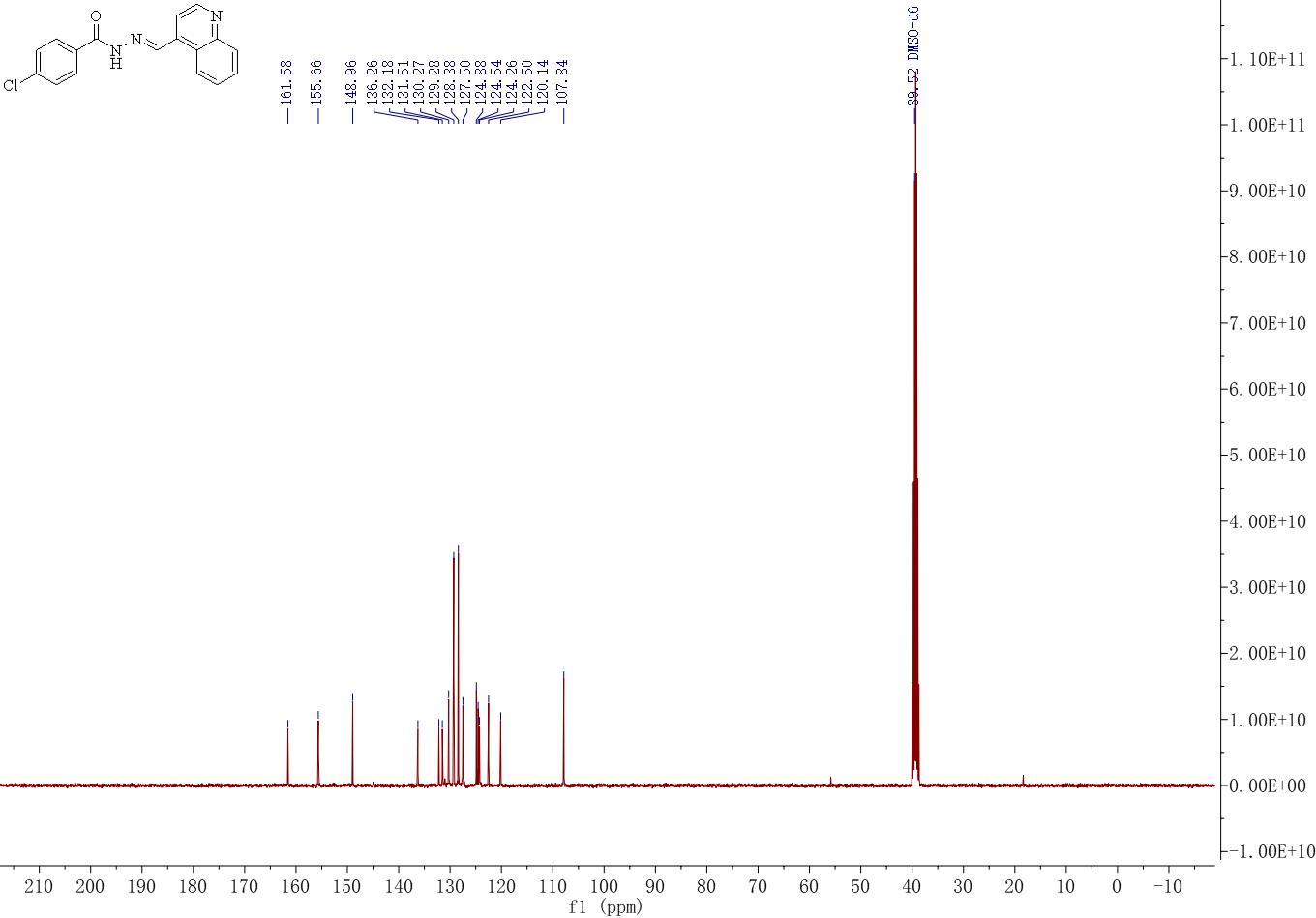


Fig 11. *^13^C NMR of* **C4** (100 MHz, DMSO)


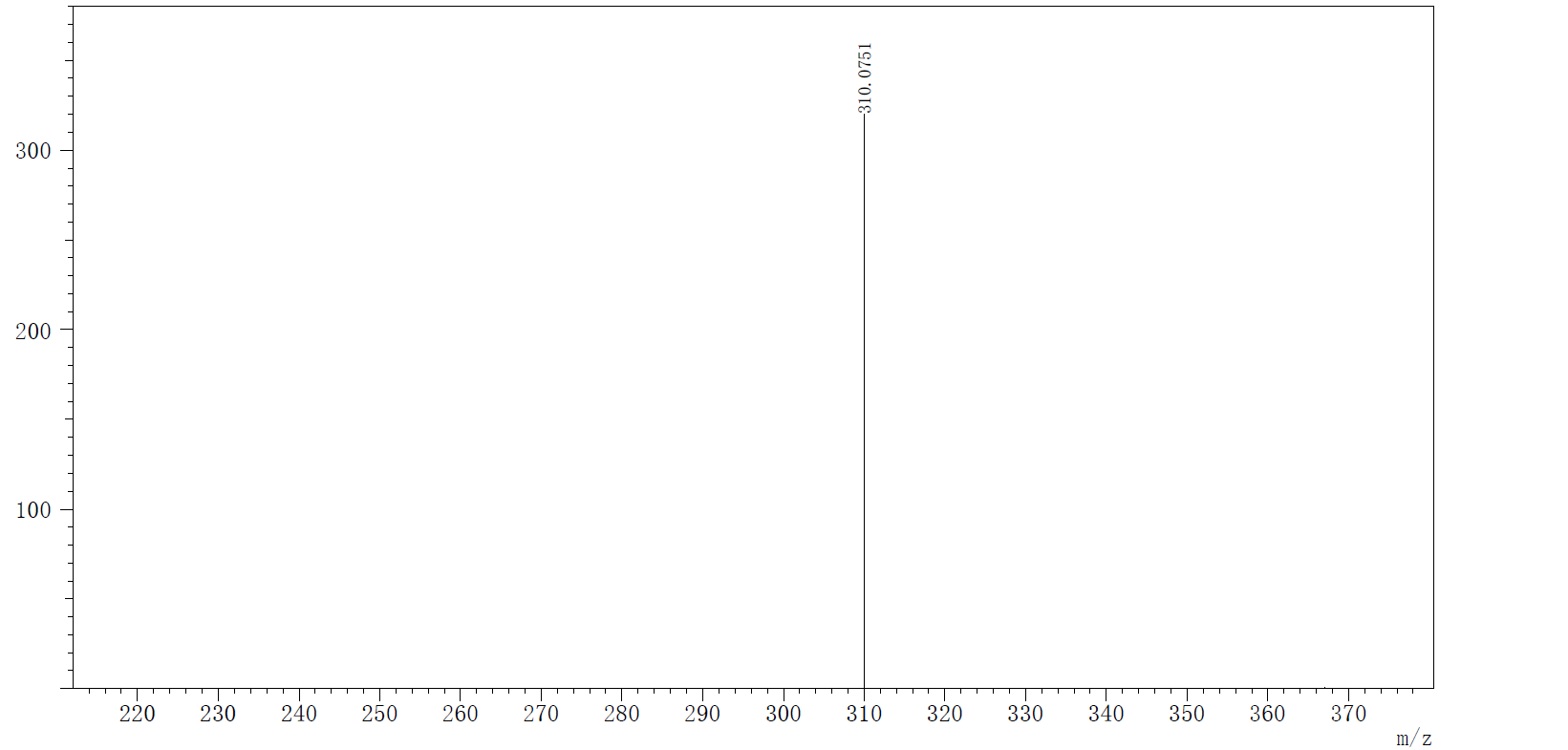


Fig 12. Mass spectrum of compound **C4**


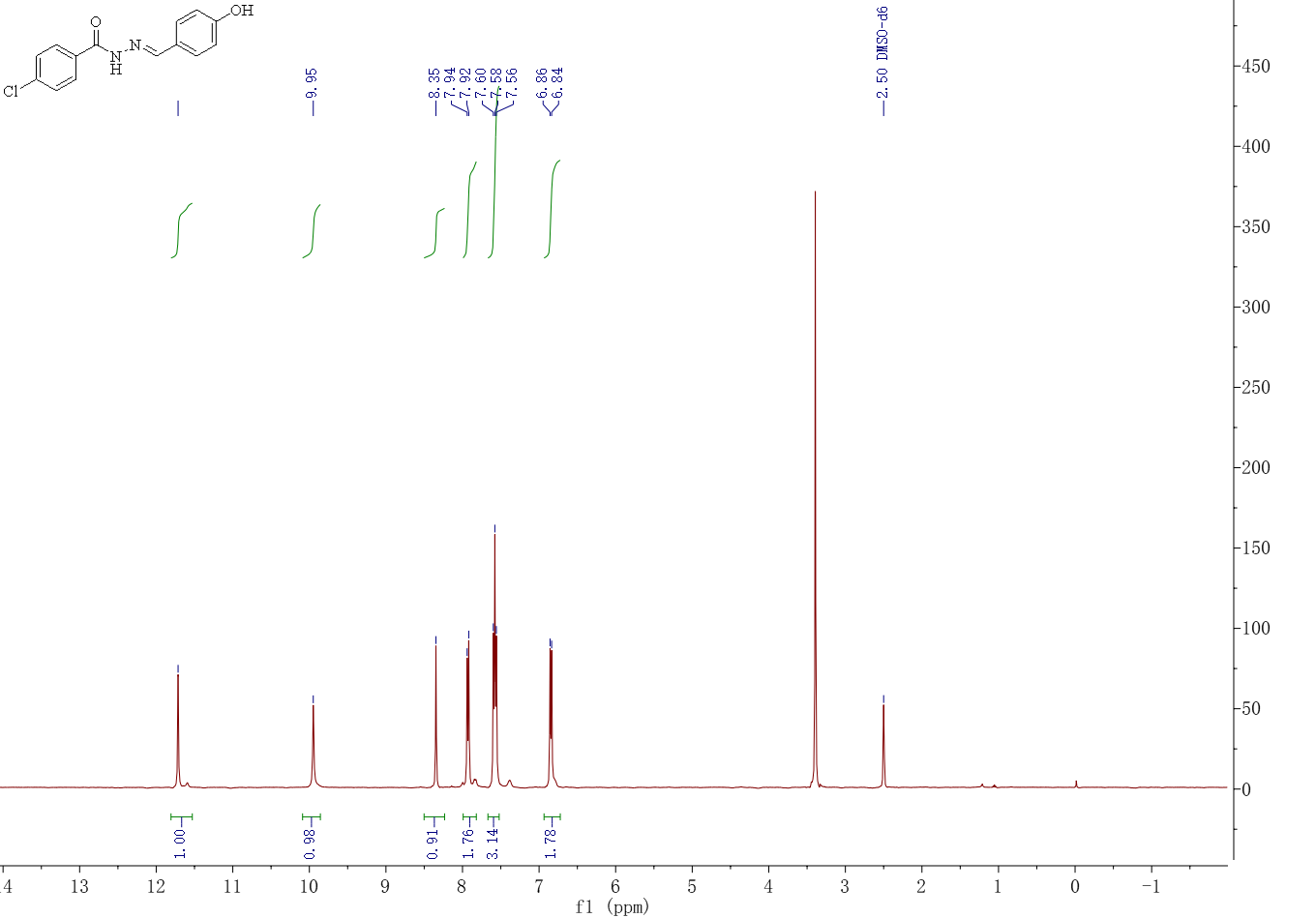


Fig 13. *^1^H NMR of* **C5** (400 MHz, DMSO)


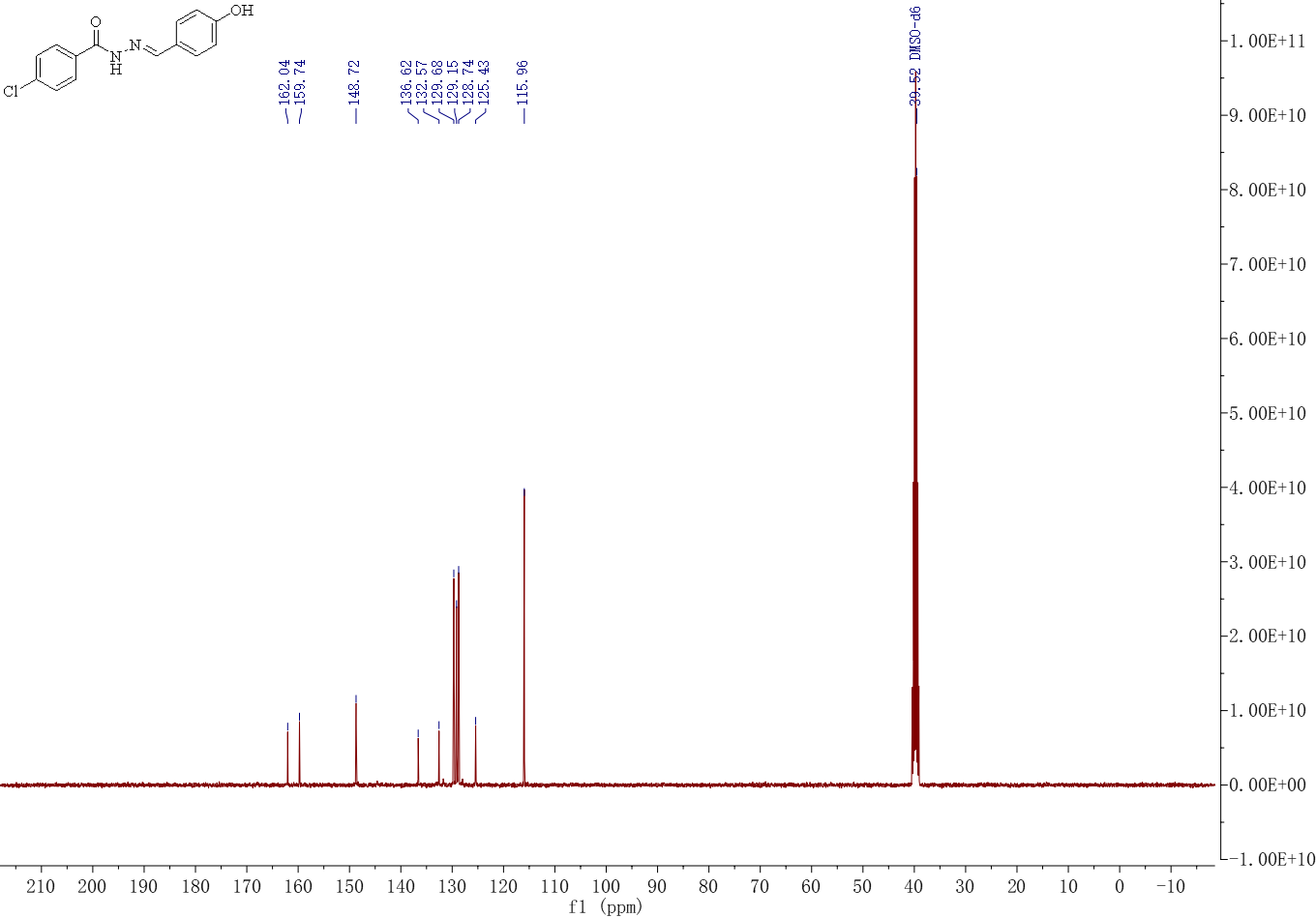


Fig 14. *^13^C NMR of* **C5** (100 MHz, DMSO)


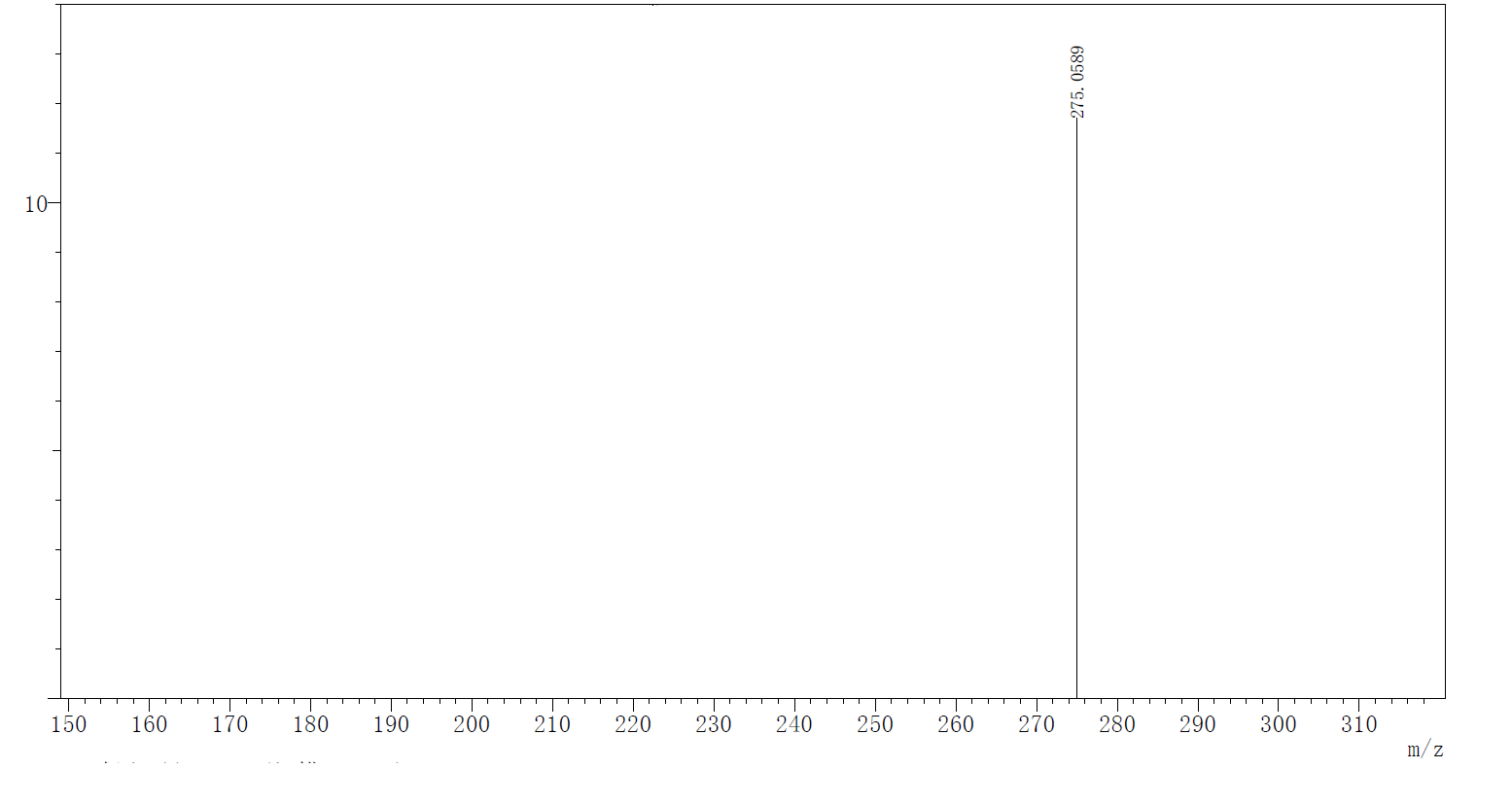


Fig 15. Mass spectrum of compound **C5**


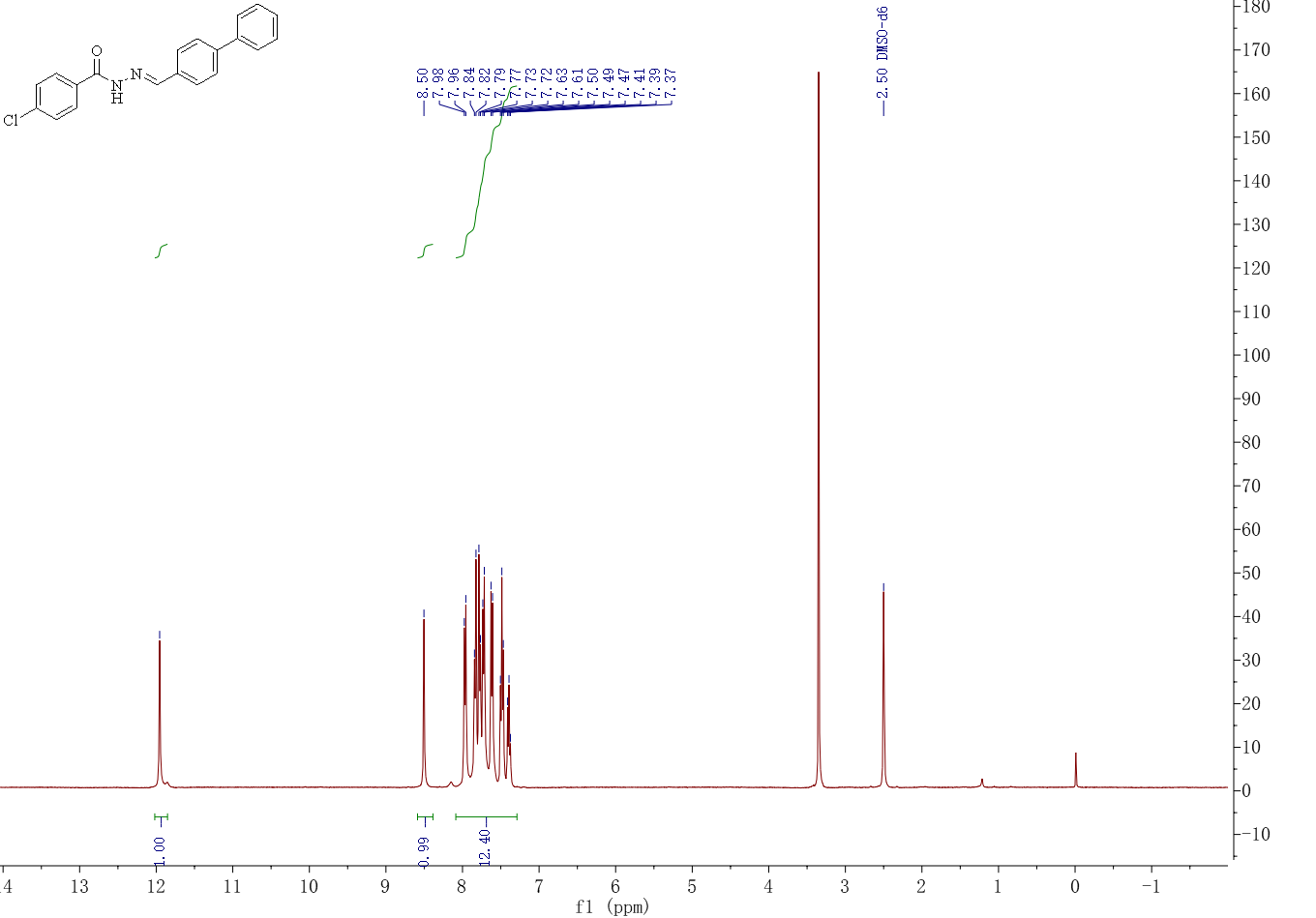


Fig 16. *^1^H NMR of* **C6** (400 MHz, DMSO)


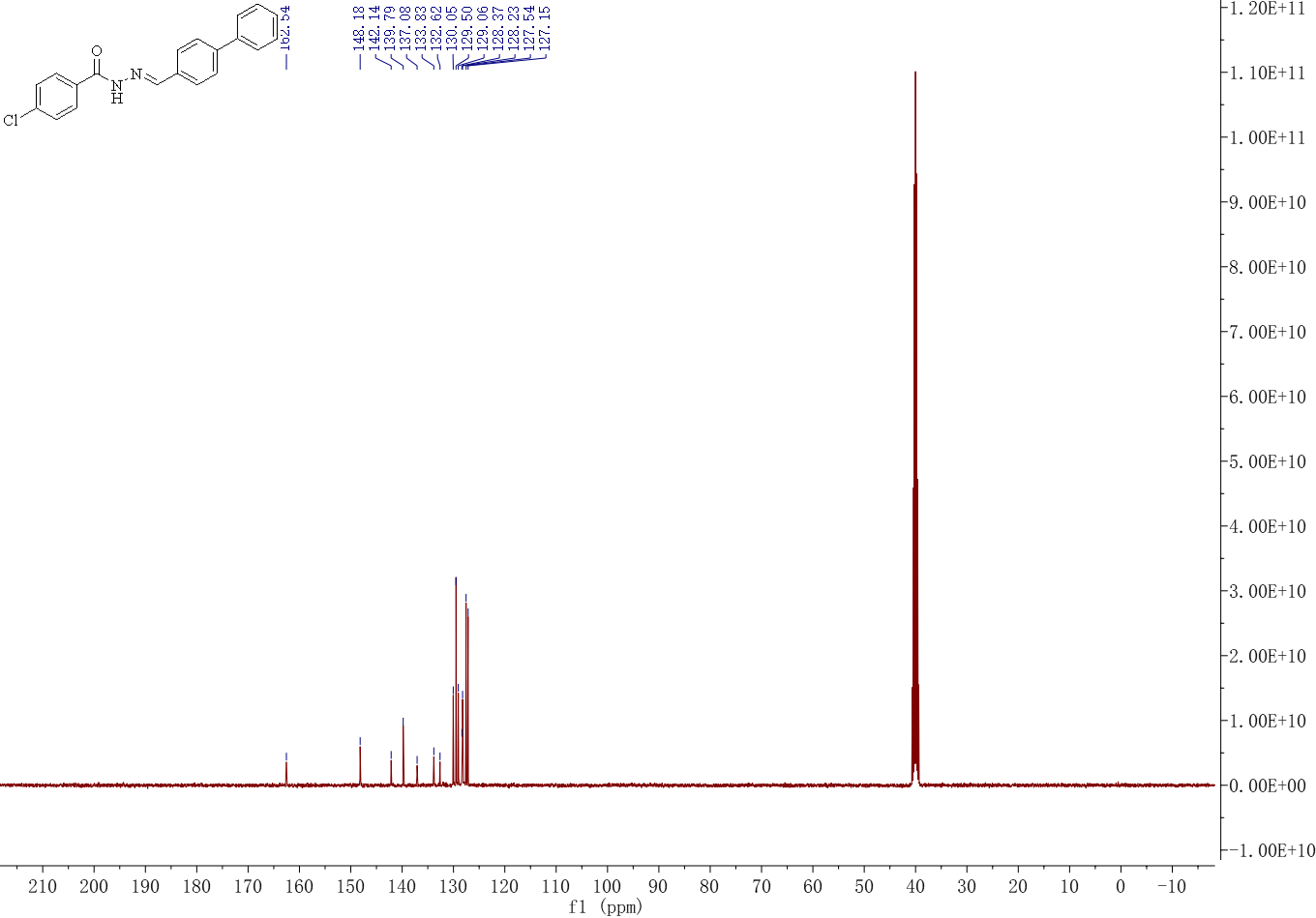


Fig 17. *^13^C NMR of* **C6** (100 MHz, DMSO)


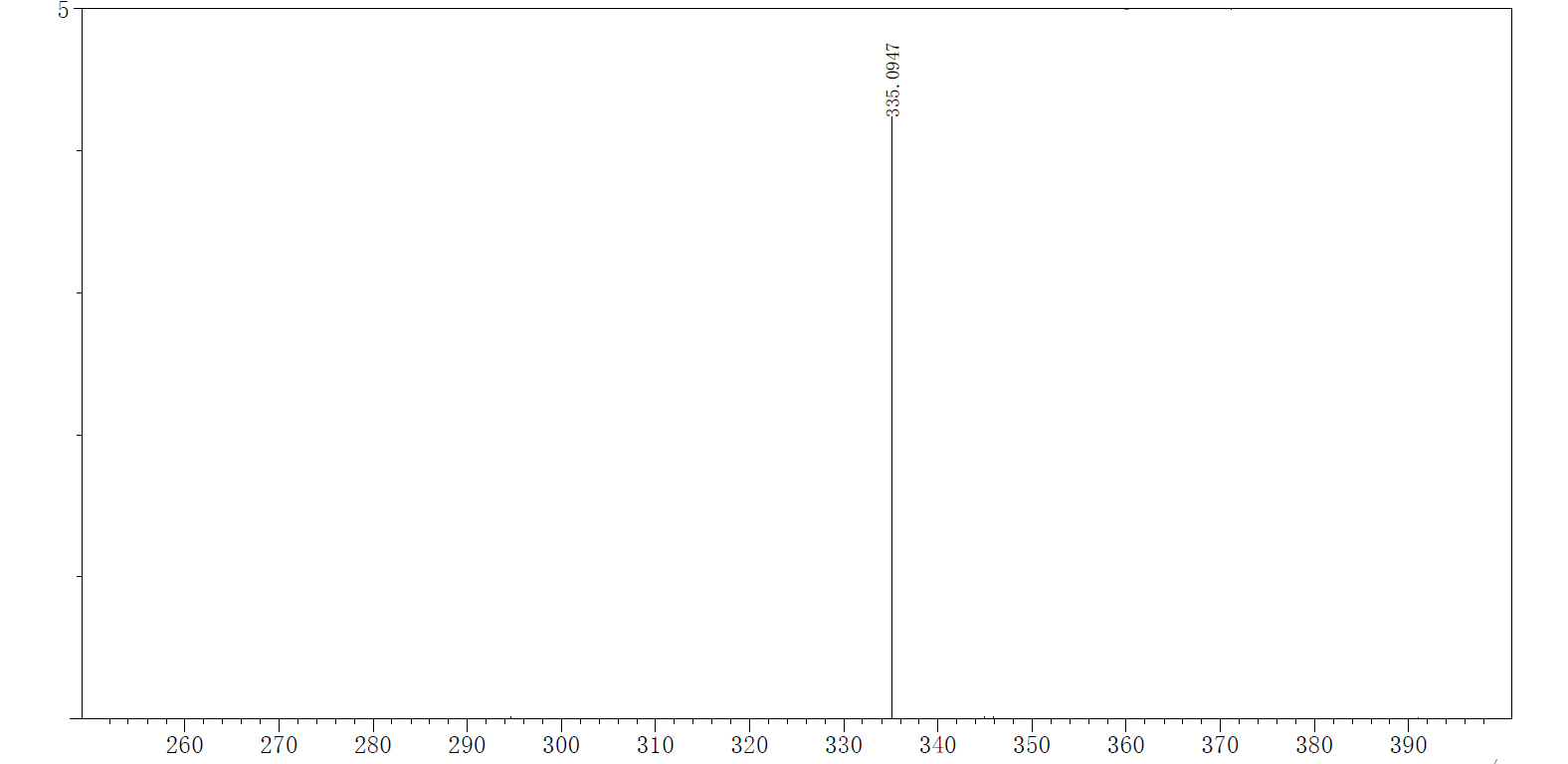


Fig 18. Mass spectrum of compound **C6**


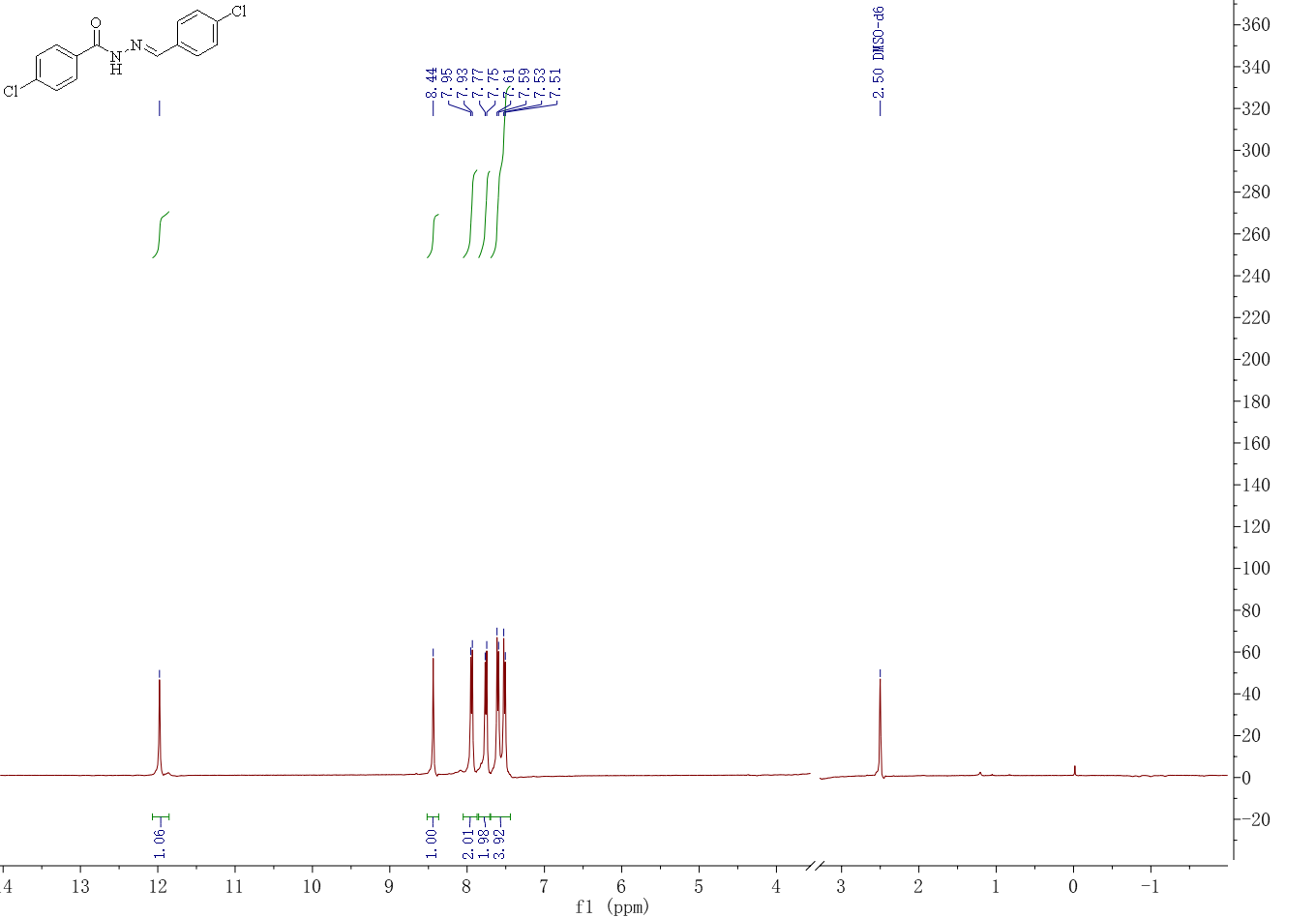


Fig 19. *^1^H NMR of* **C7** (400 MHz, DMSO)


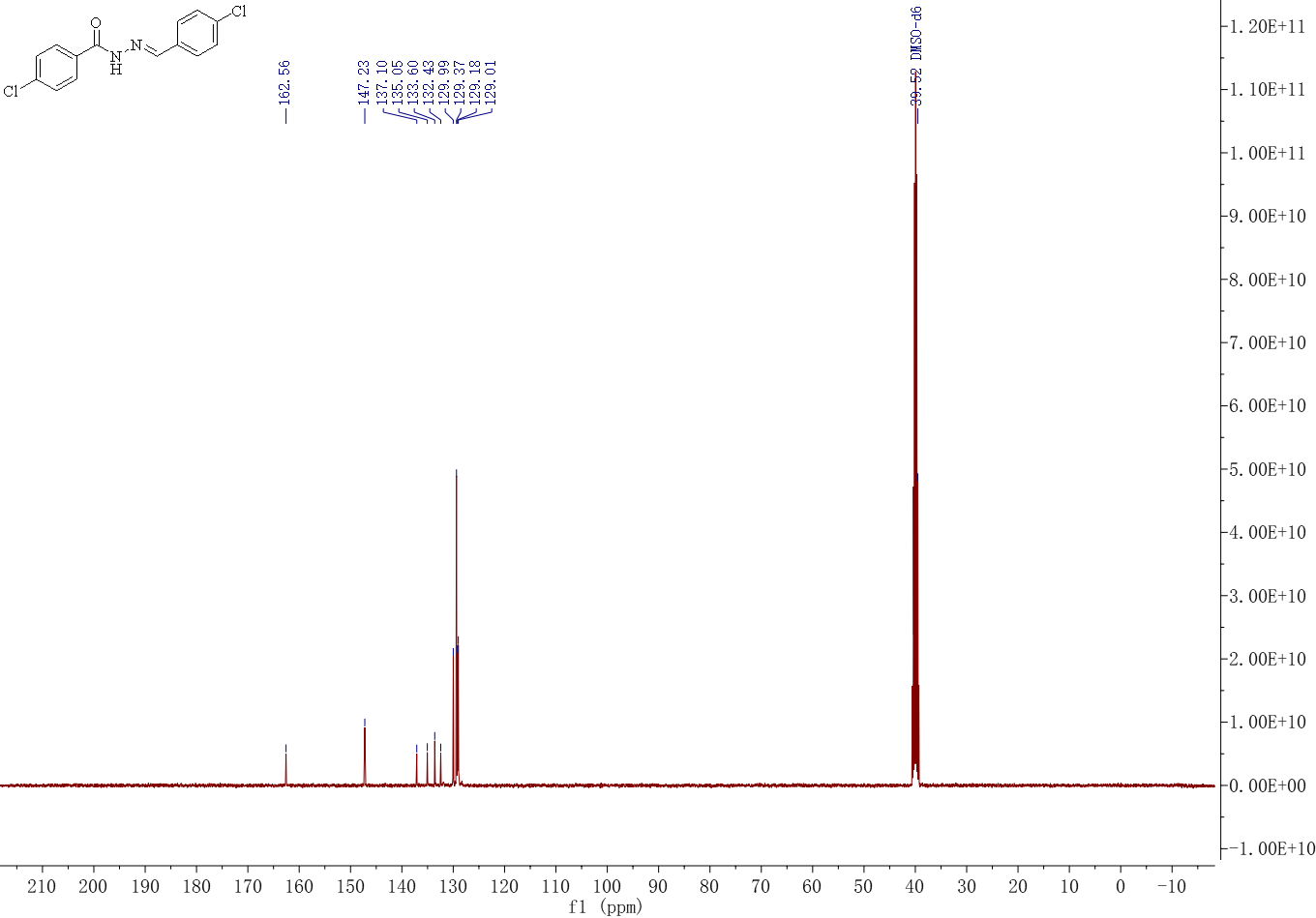


Fig 20. *^13^C NMR of* **C7** (100 MHz, DMSO)


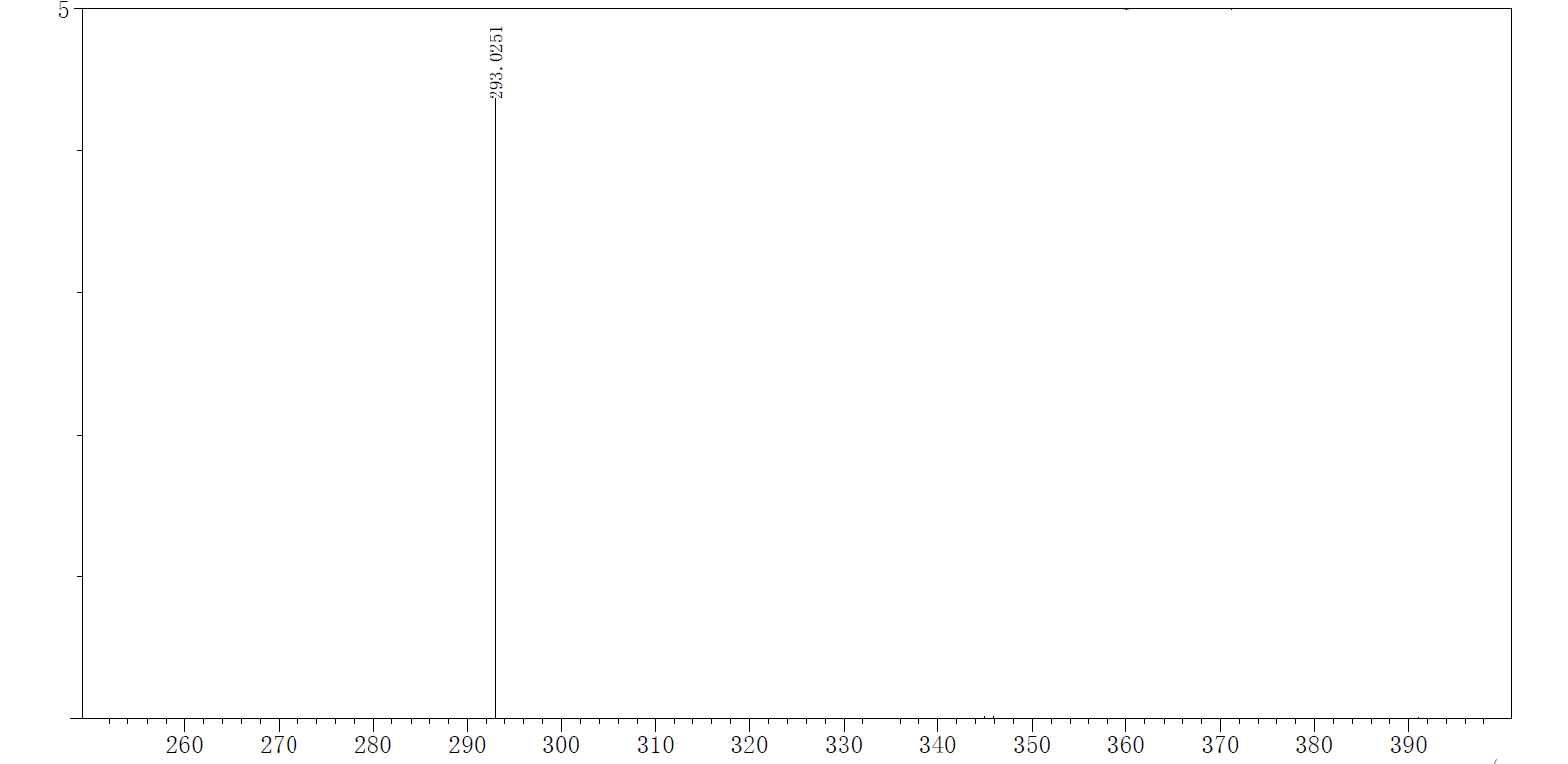


Fig 21. Mass spectrum of compound **C7**


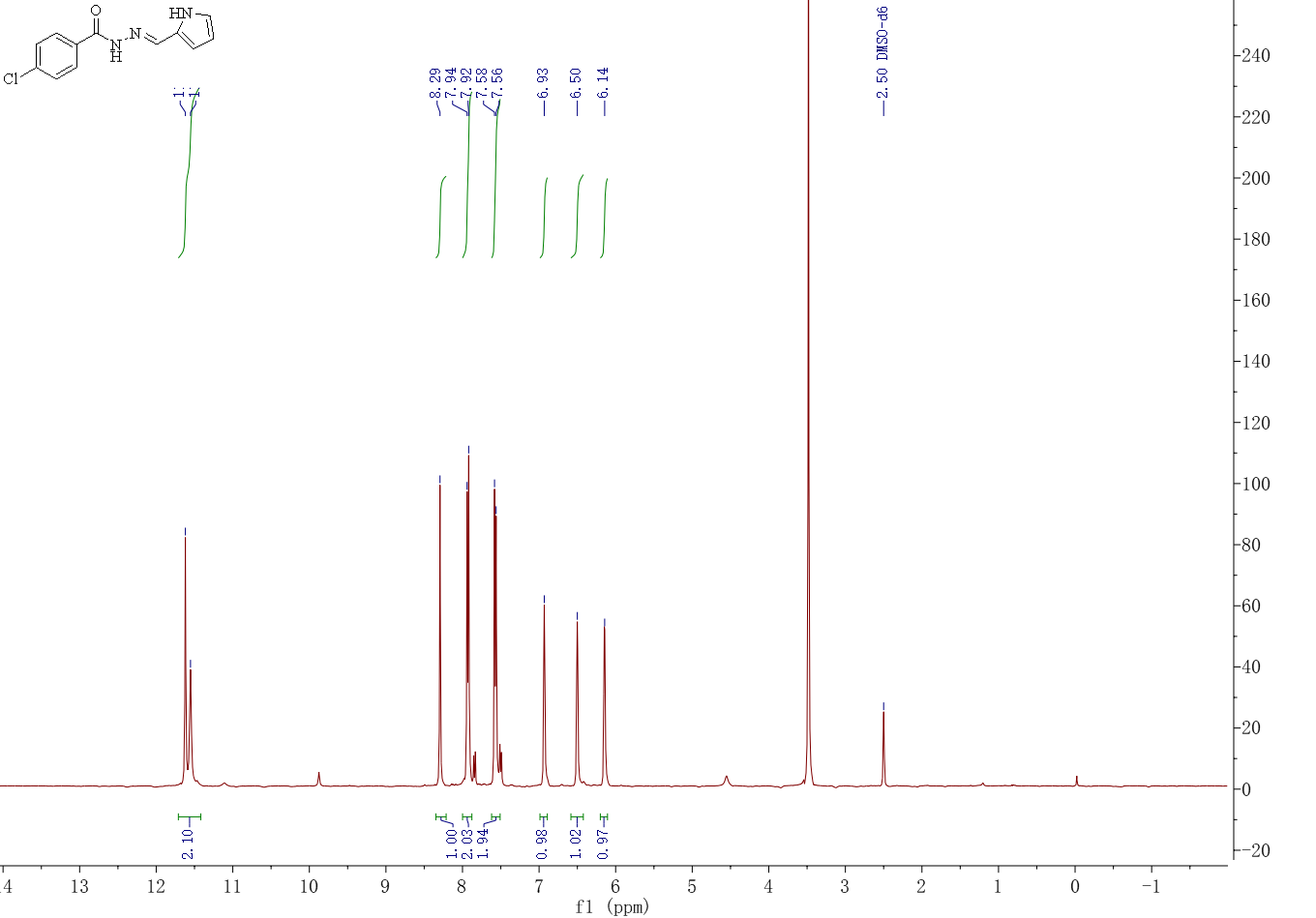


Fig 22. *^1^H NMR of* **C8** (400 MHz, DMSO)


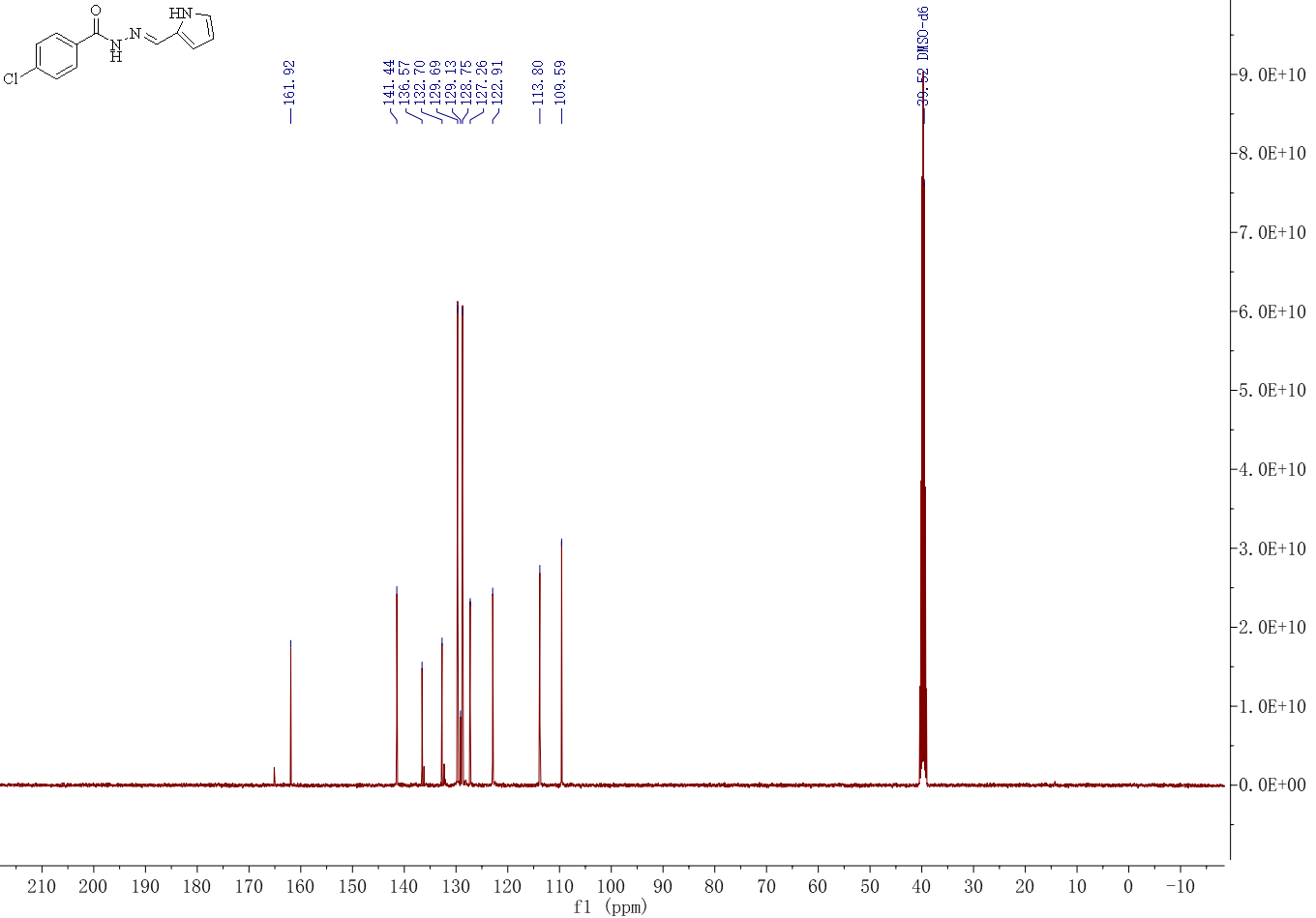


Fig 23. *^13^C NMR of* **C8** (100 MHz, DMSO)


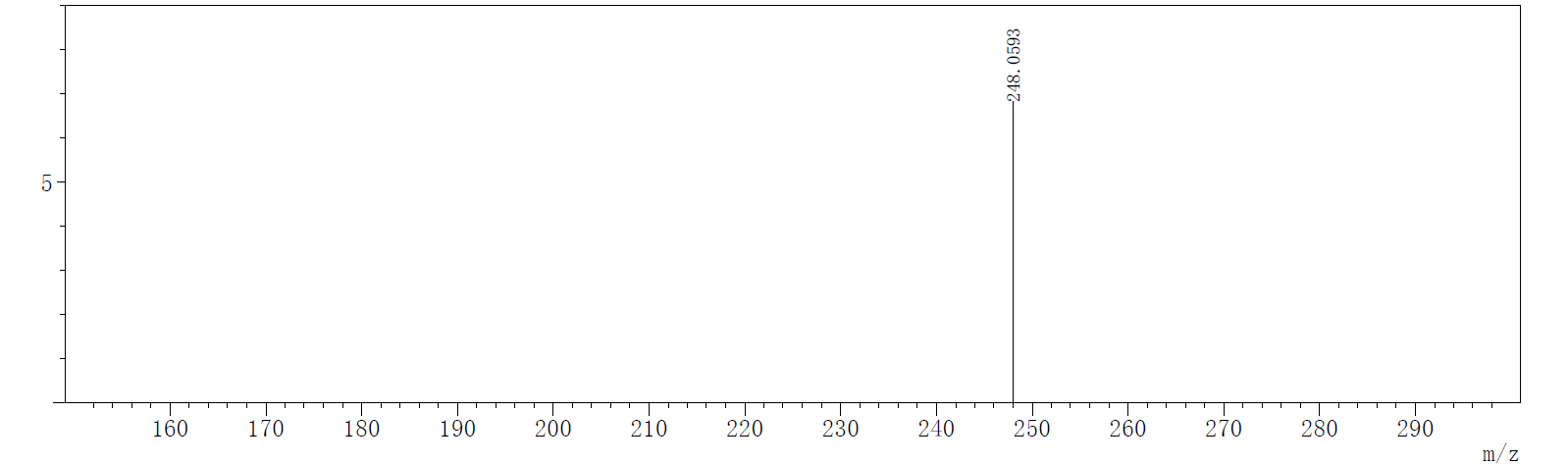


Fig 24. Mass spectrum of compound **C8**


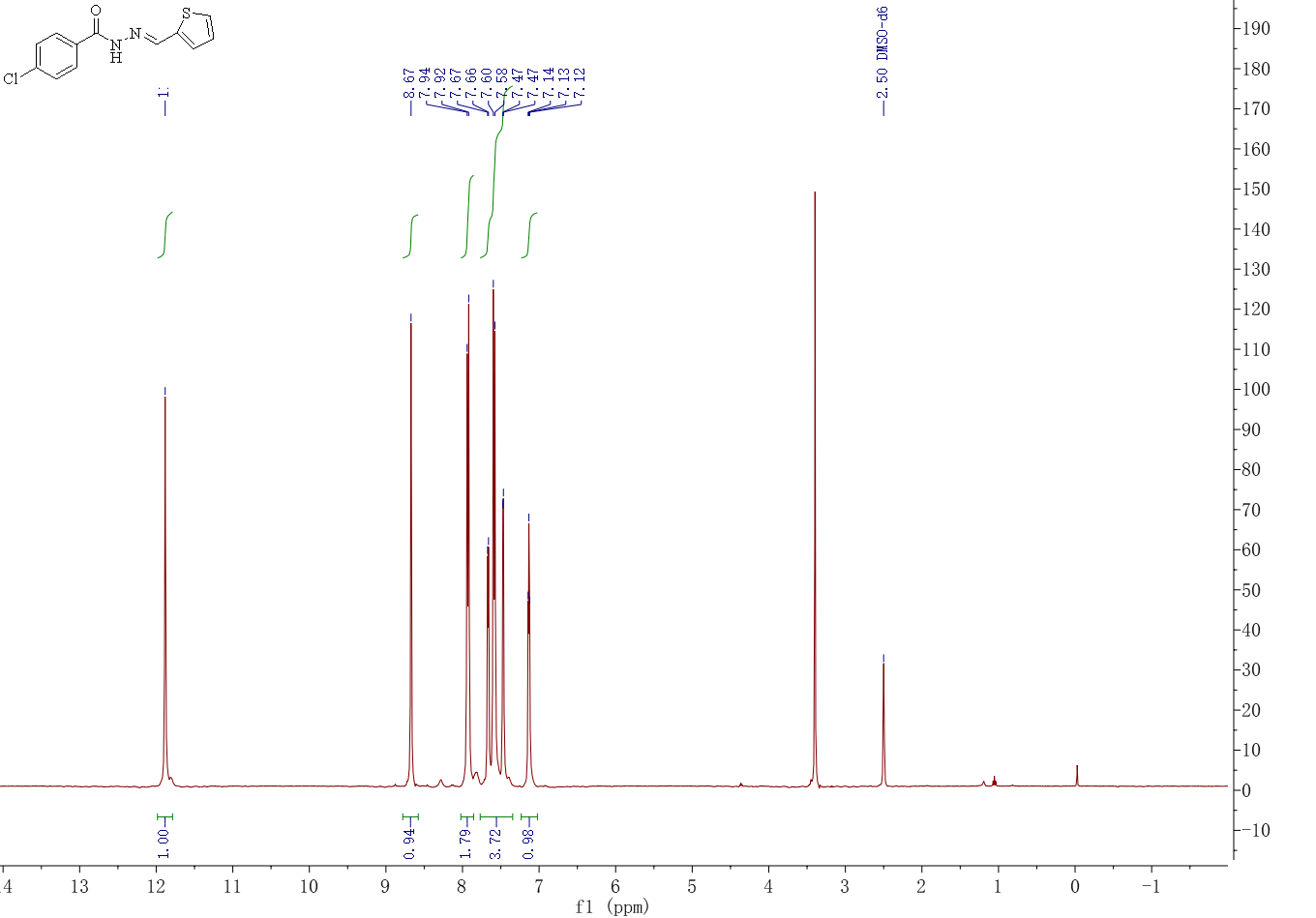


Fig 25. *^1^H NMR of* **C9** (400 MHz, DMSO)


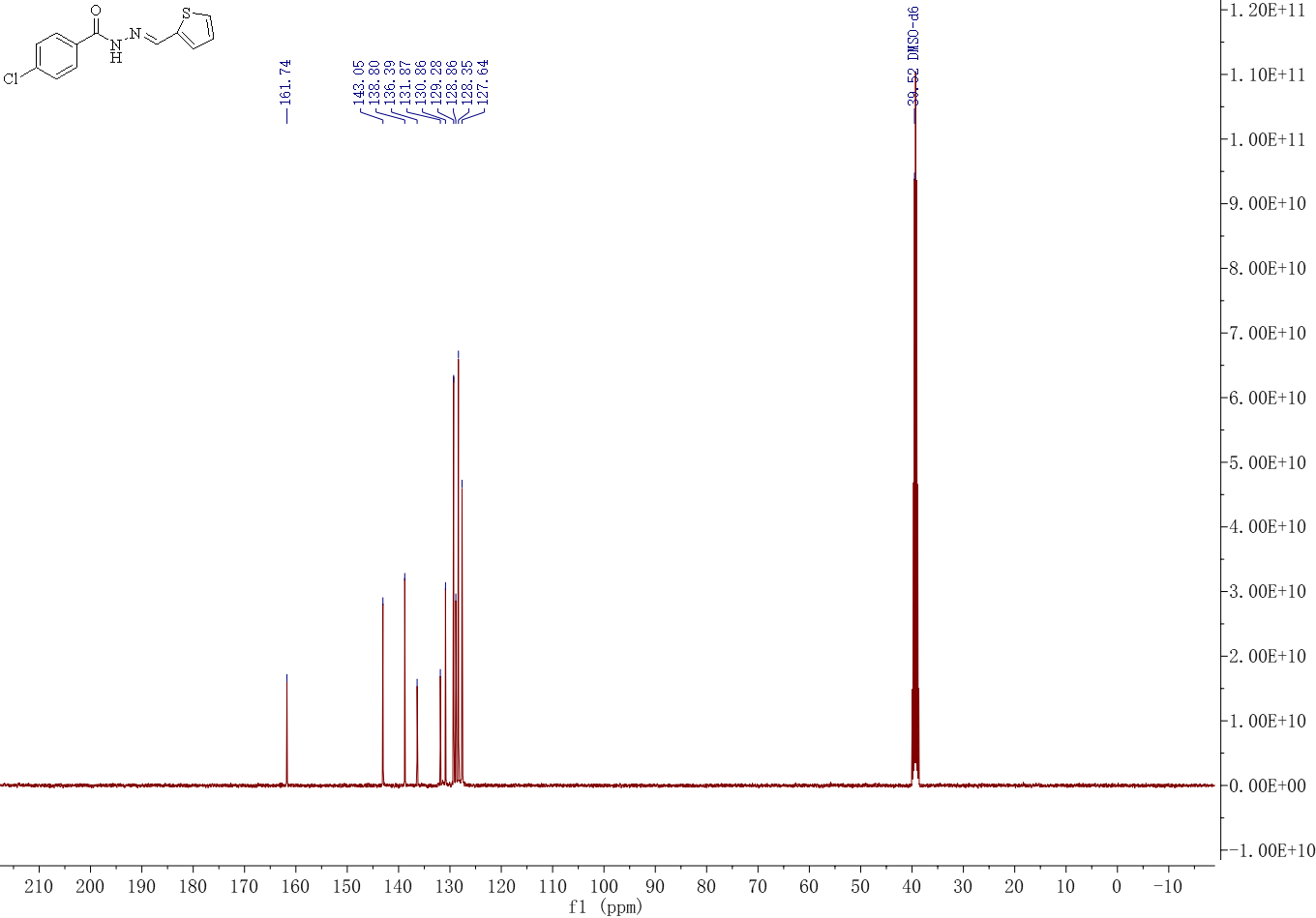


Fig 26. *^13^C NMR of* **C9** (100 MHz, DMSO)


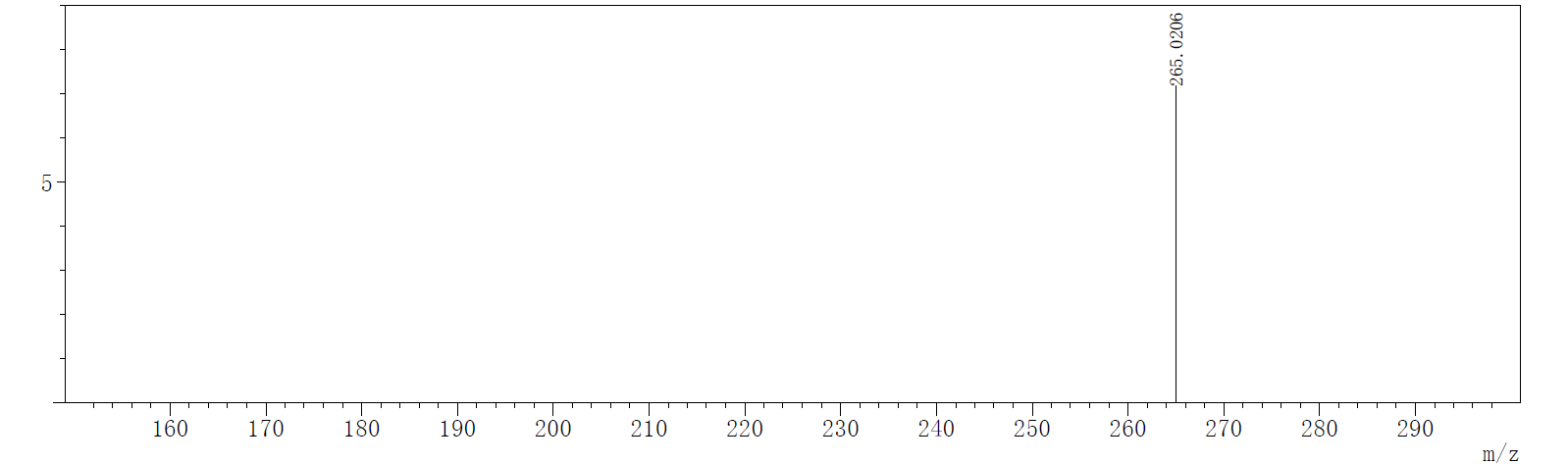


Fig 27. Mass spectrum of compound **C9**


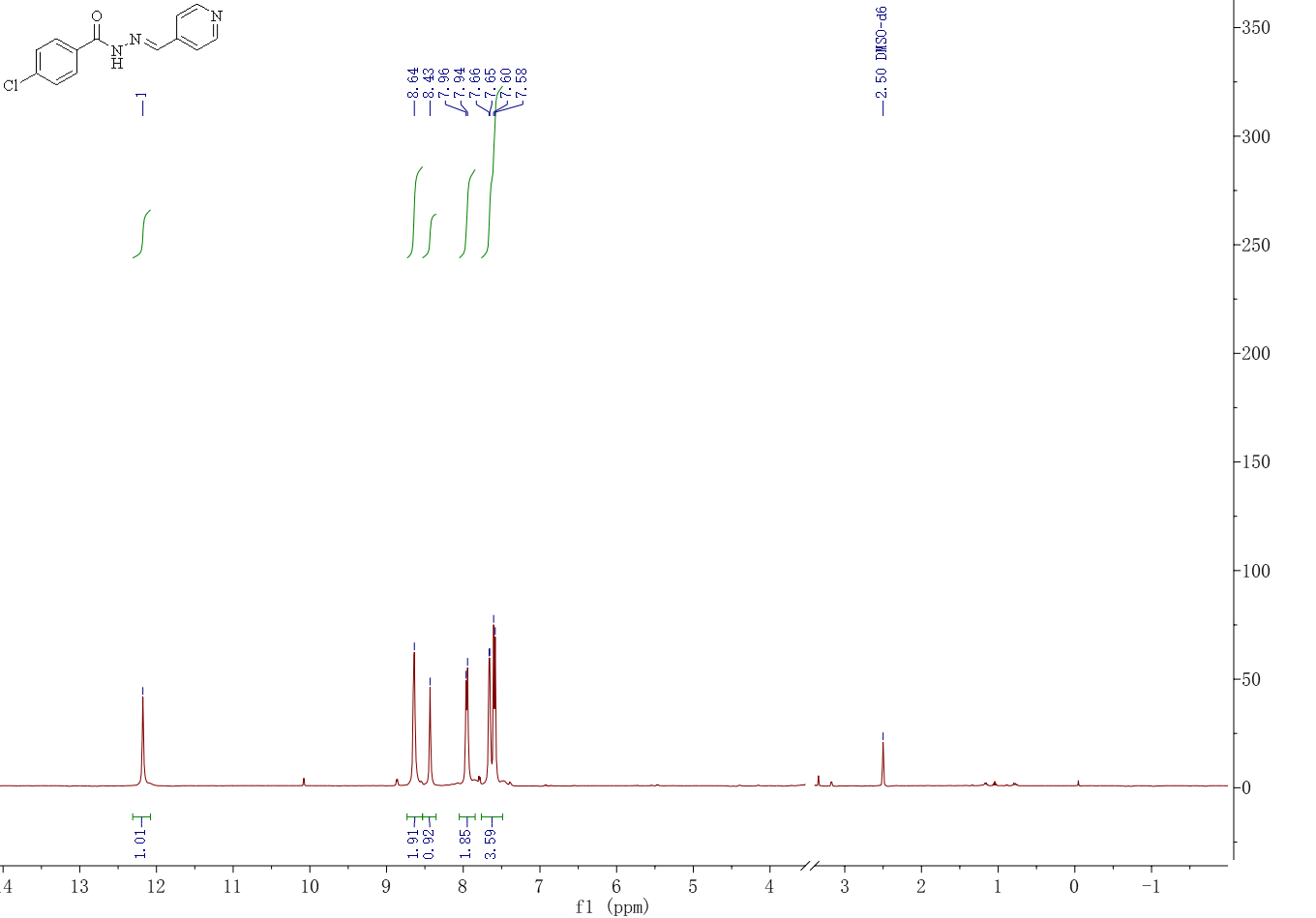


Fig 28. *^1^H NMR of* **C10** (400 MHz, DMSO)


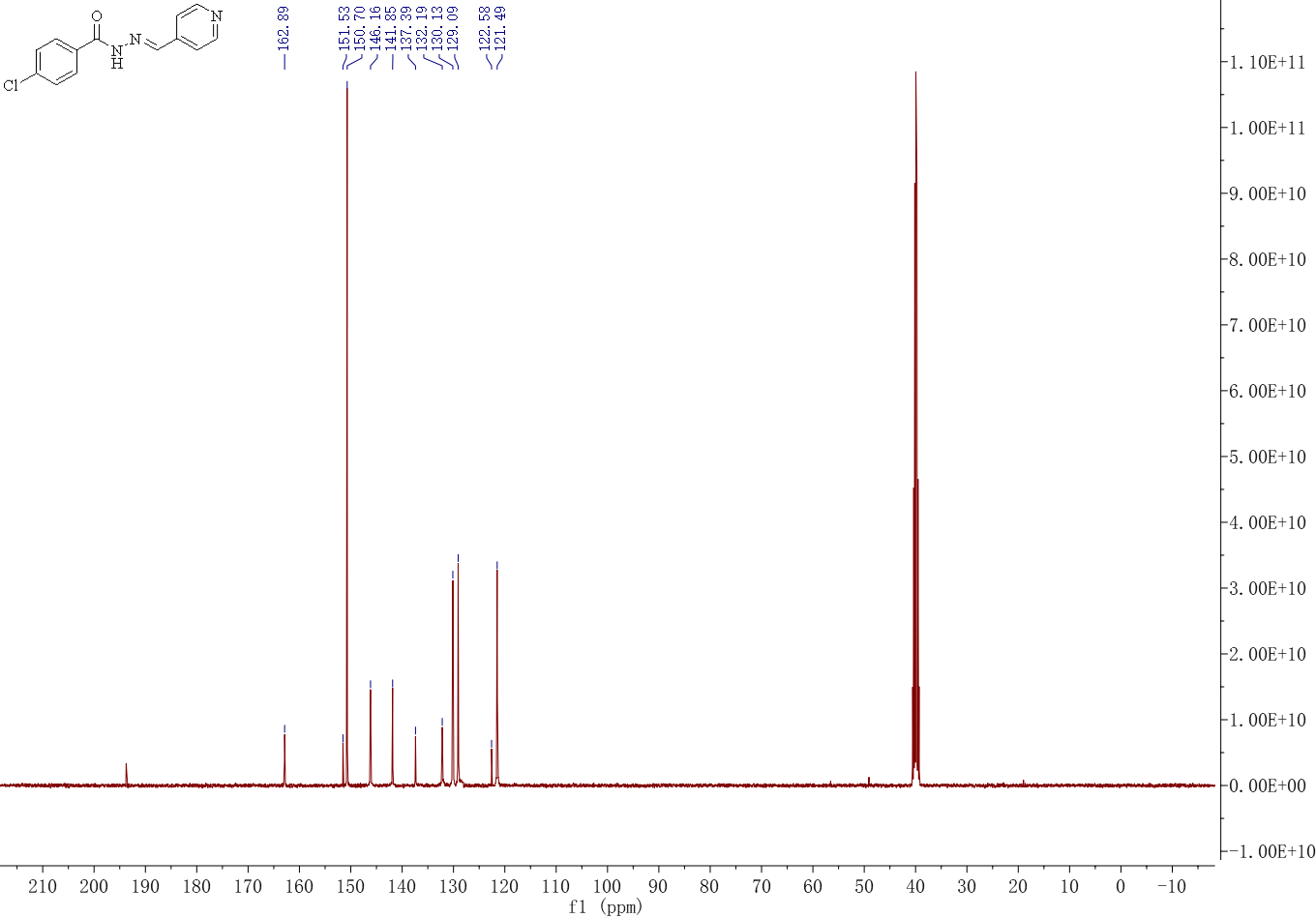


Fig 29. *^13^C NMR of* **C10** (100 MHz, DMSO)


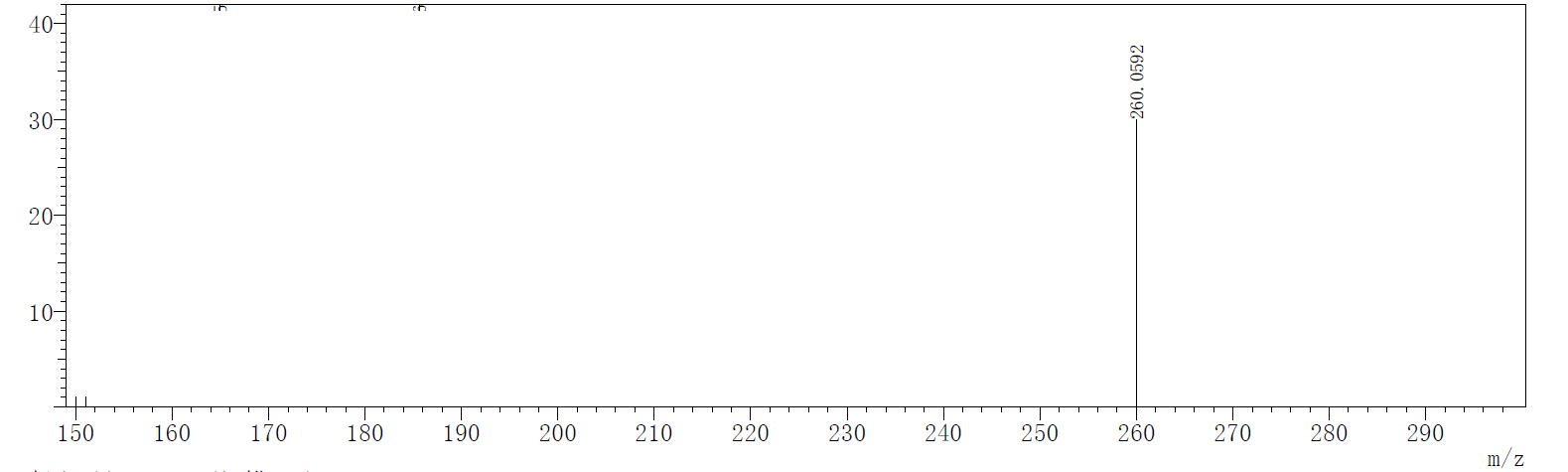


Fig 30. Mass spectrum of compound **C10**


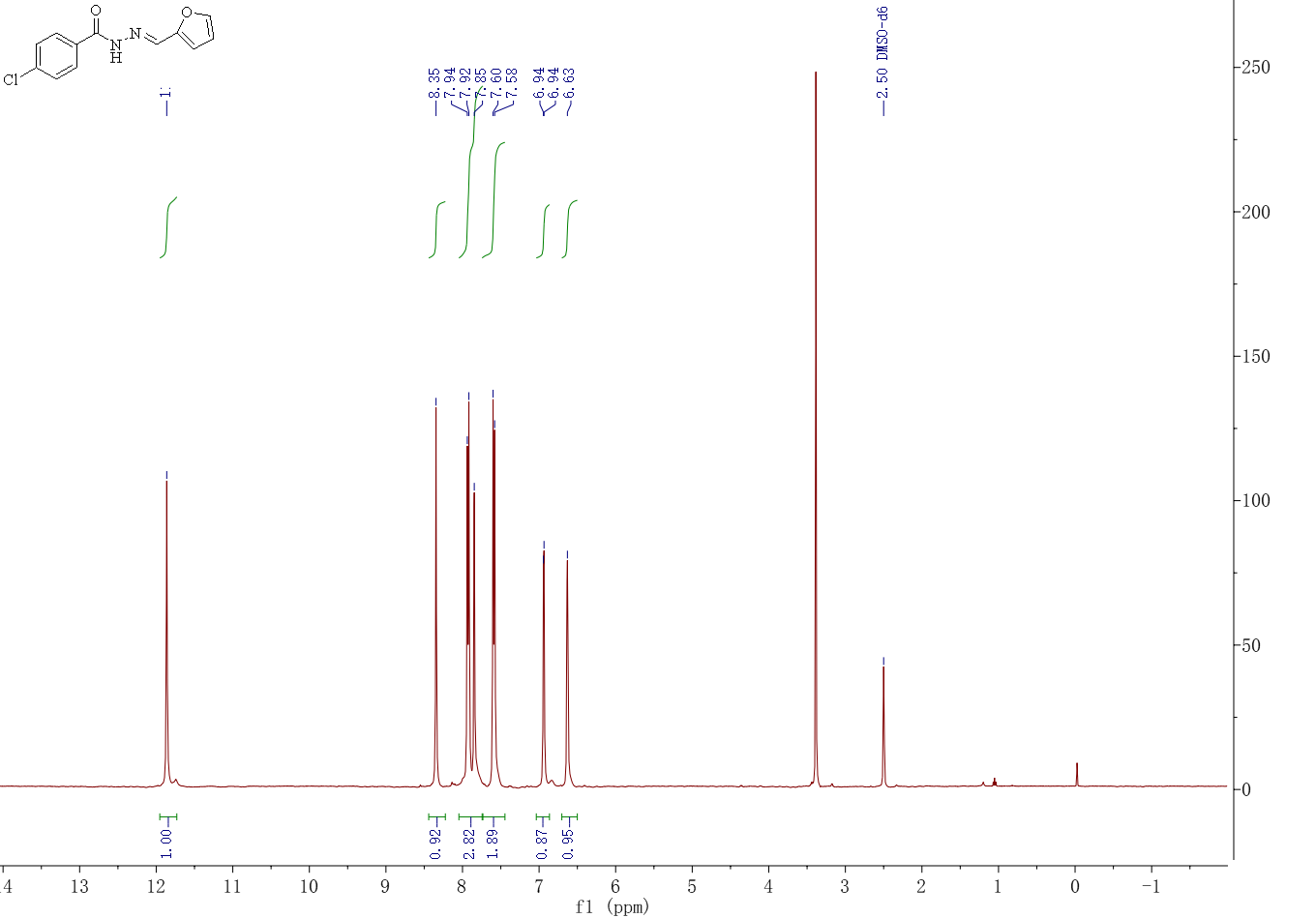


Fig 31. *^1^H NMR of* **C11** (400 MHz, DMSO)


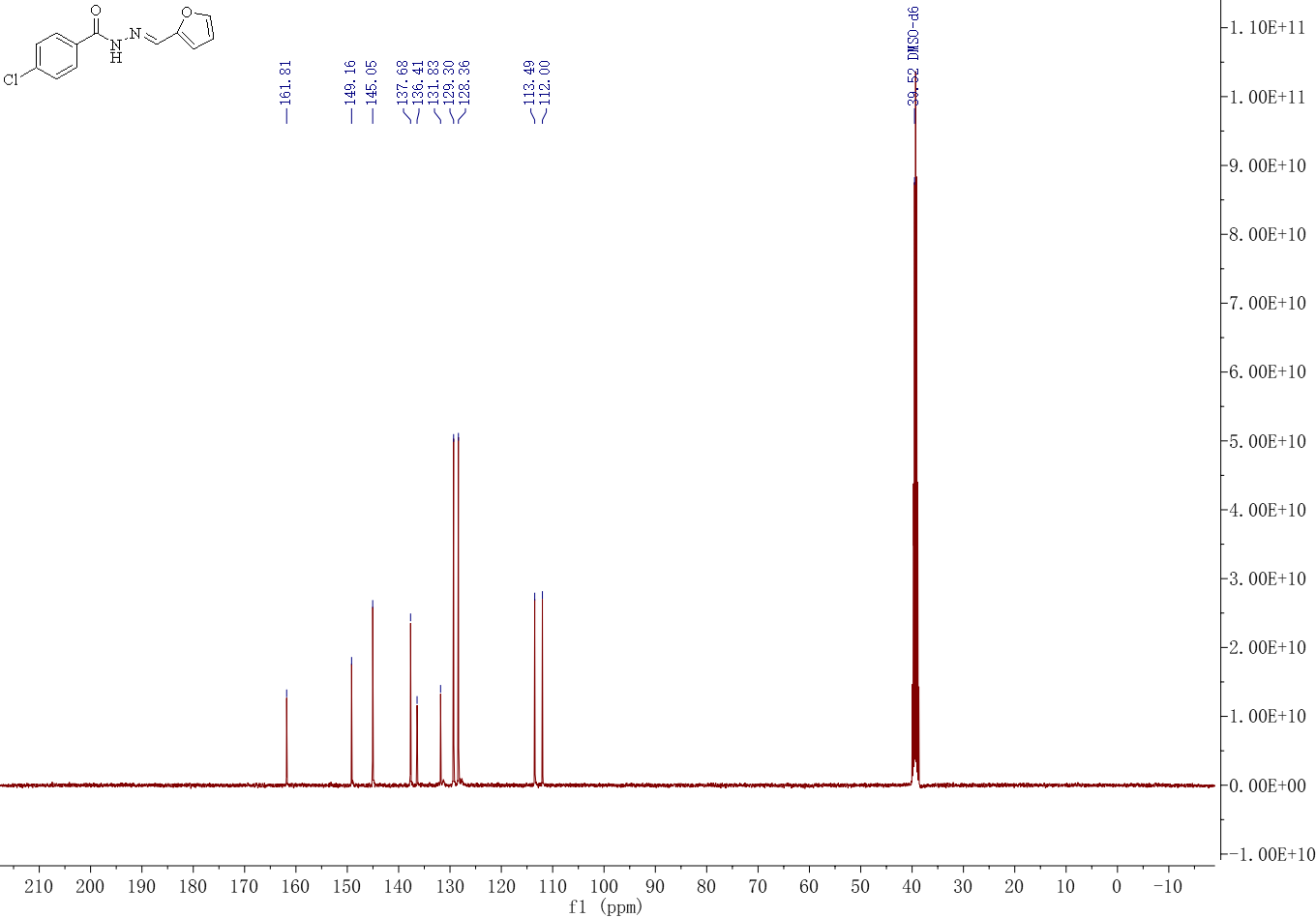


Fig 32. *^13^C NMR of* **C11** (100 MHz, DMSO)


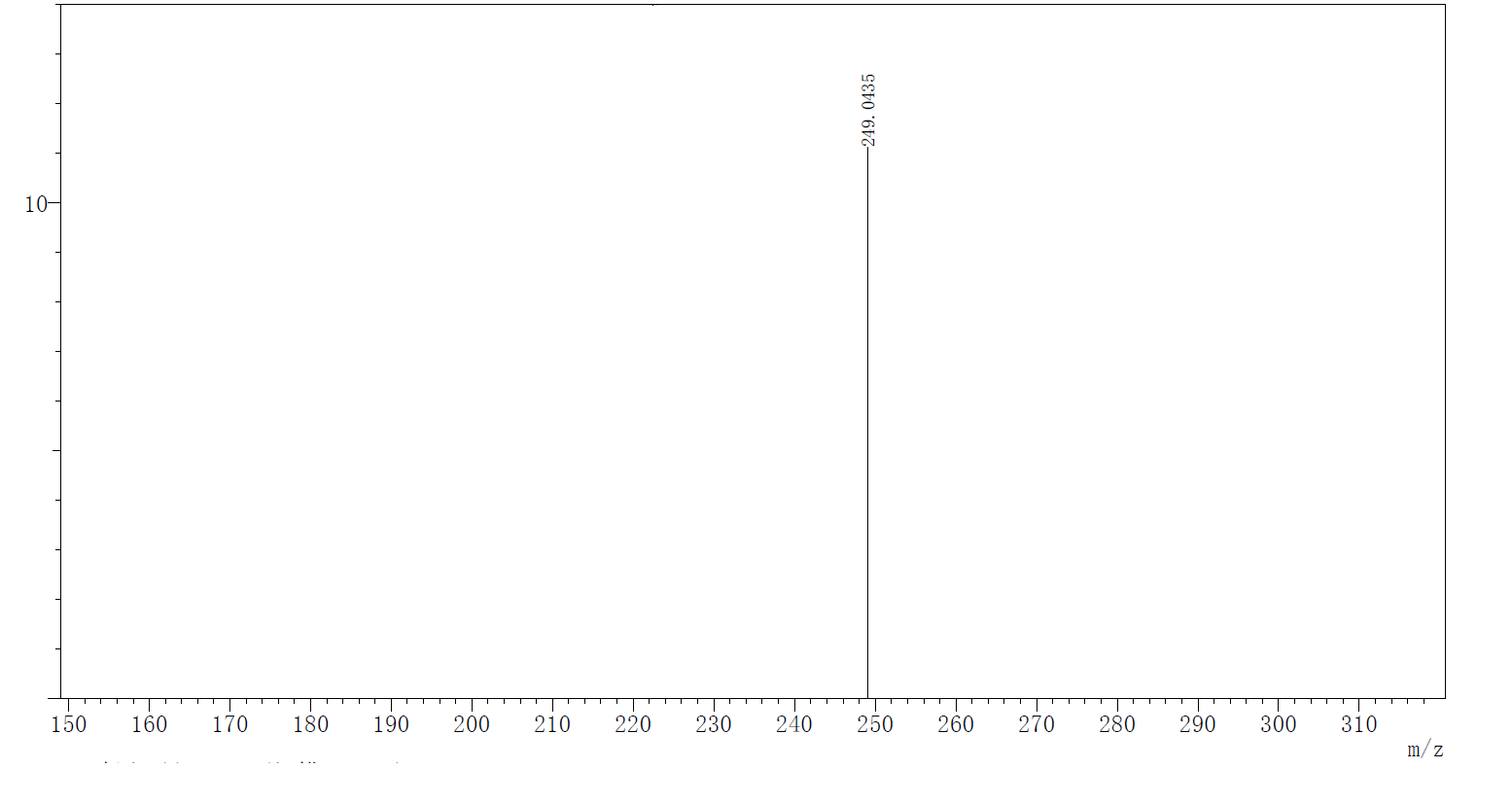


Fig 33. Mass spectrum of compound **C11**


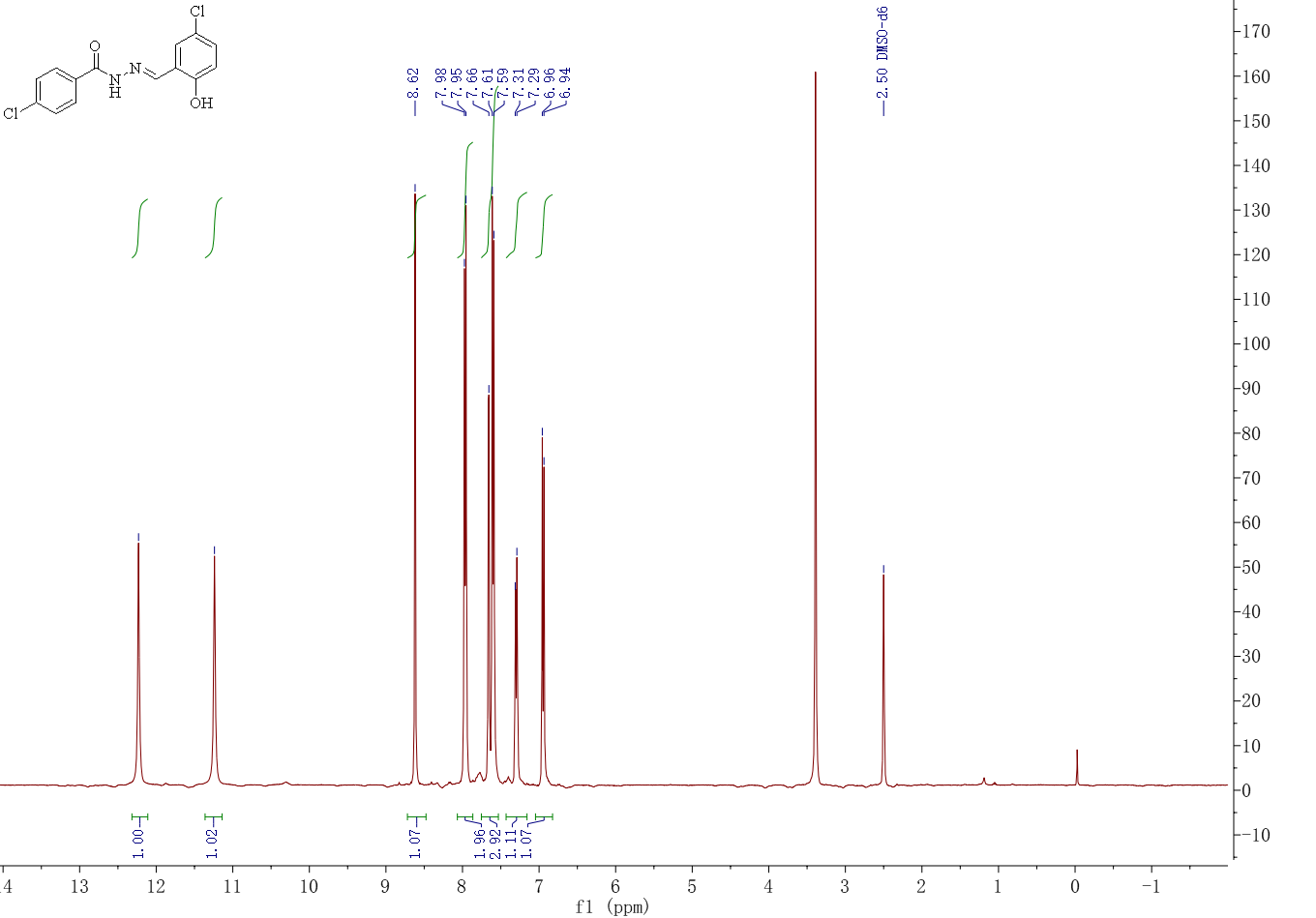


Fig 34. *^1^H NMR of* **C12** (400 MHz, DMSO)


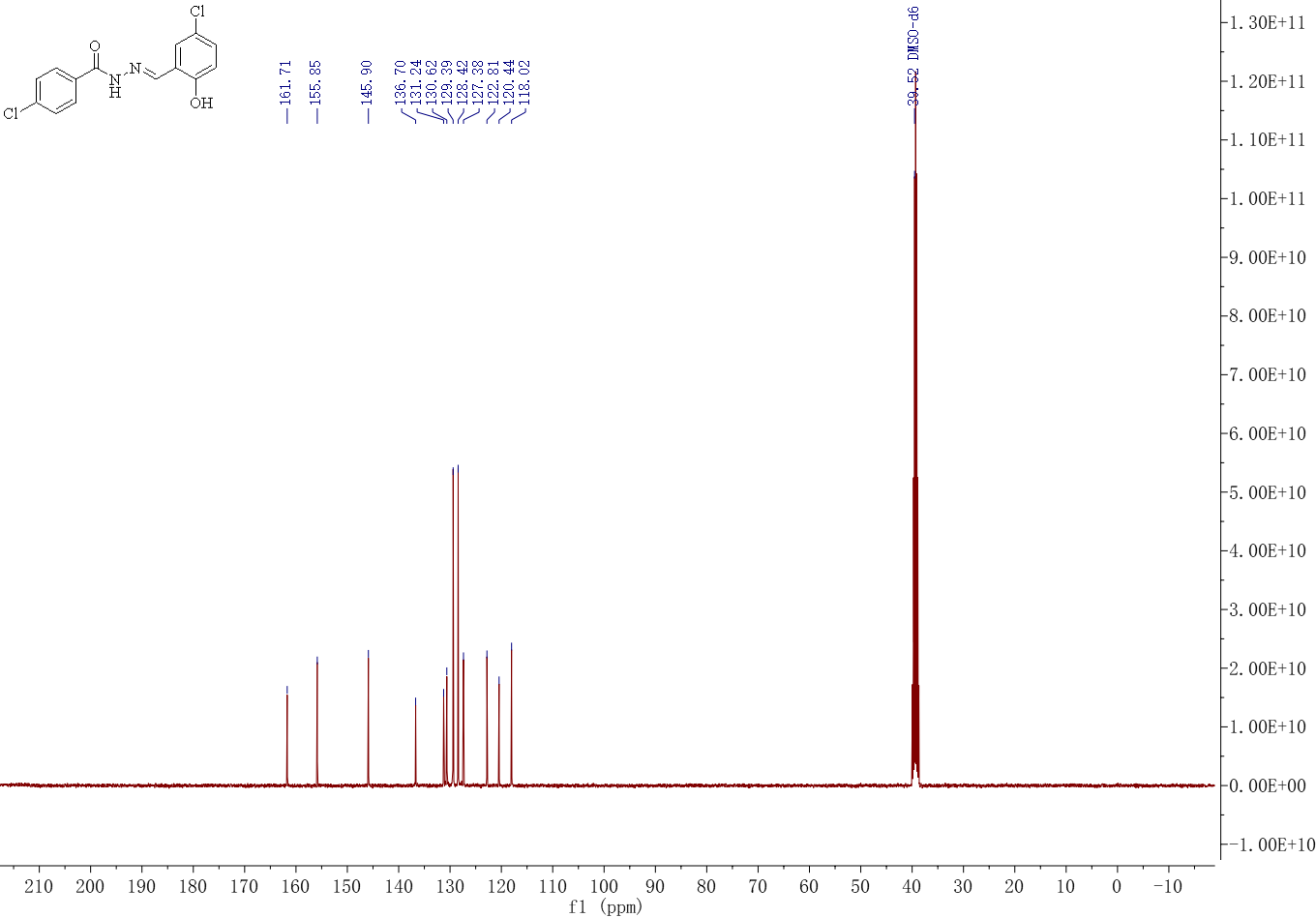


Fig 35. *^13^C NMR of* **C12** (100 MHz, DMSO)


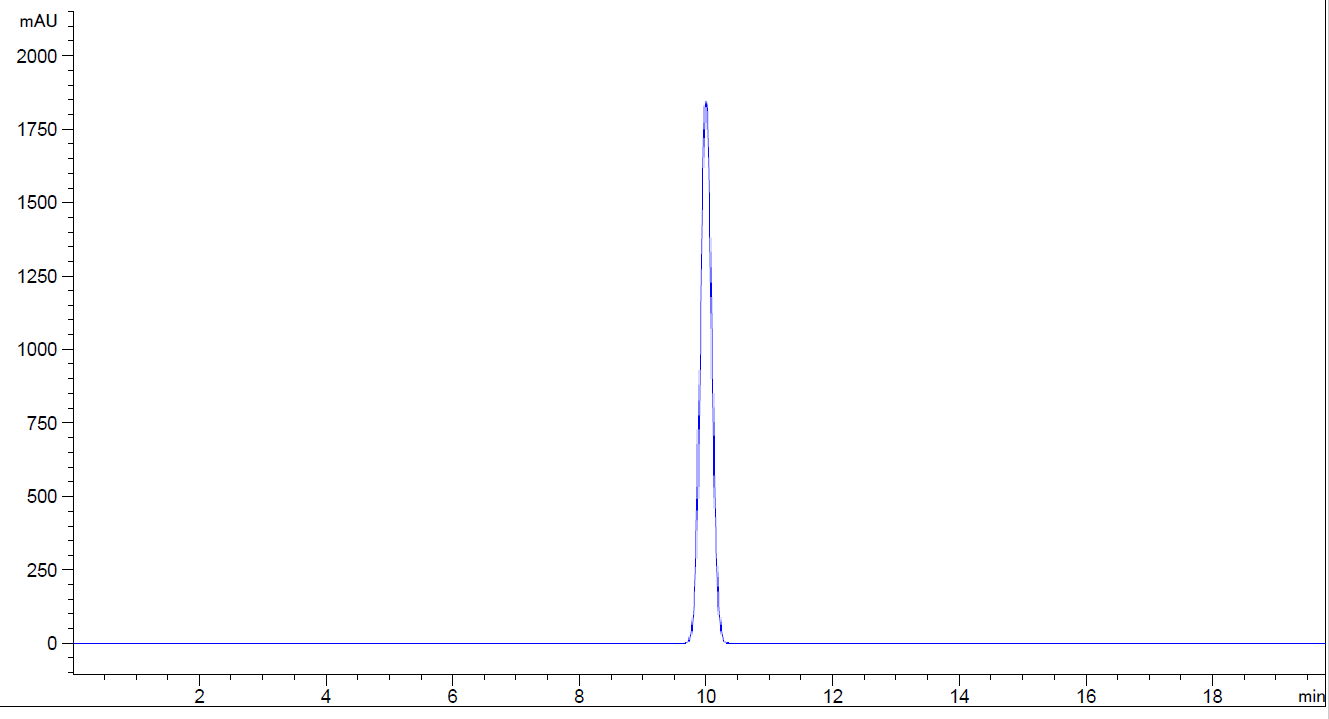


Fig 36. HPLC chromatograms compound **C12**


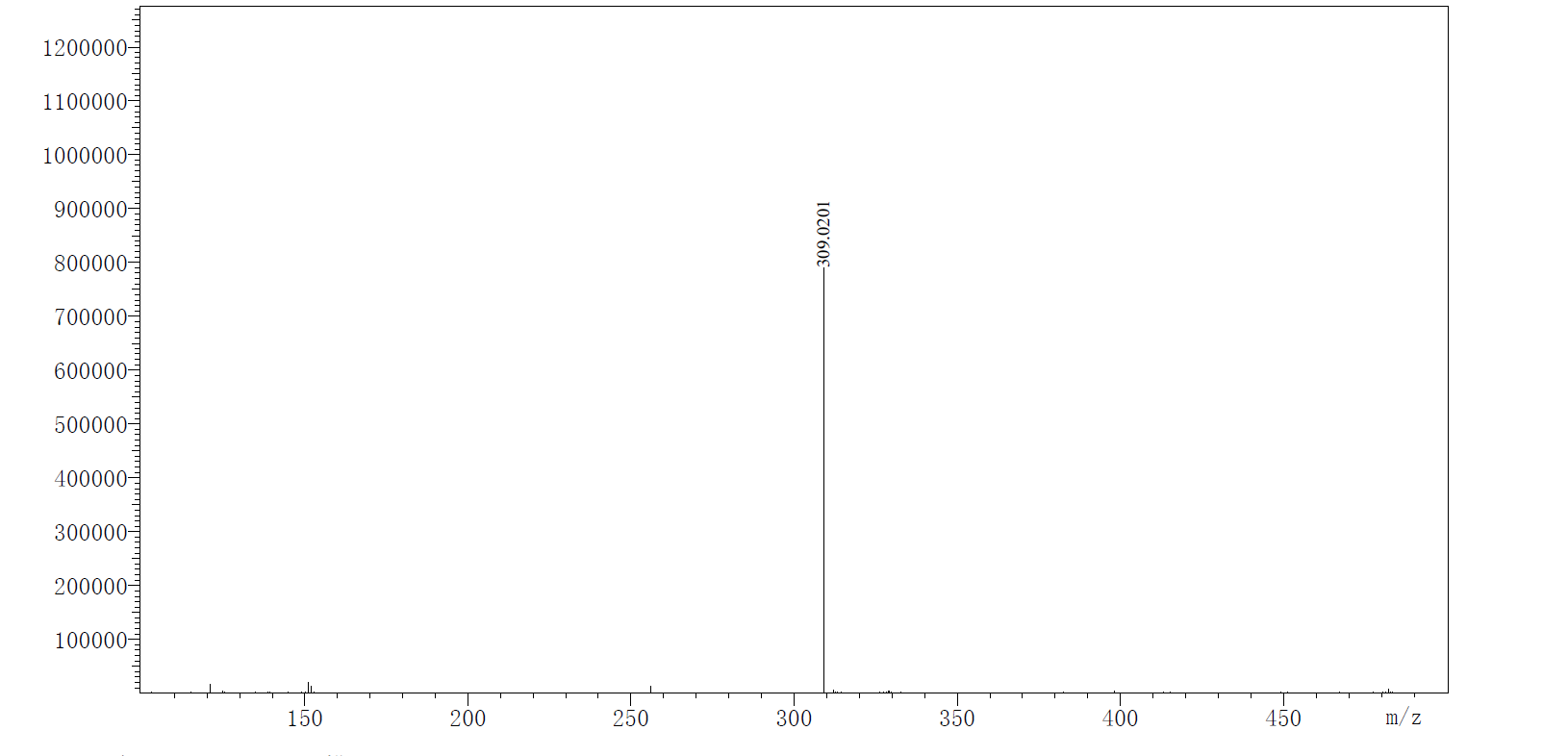


Fig 37. Mass spectrum of compound **C12**


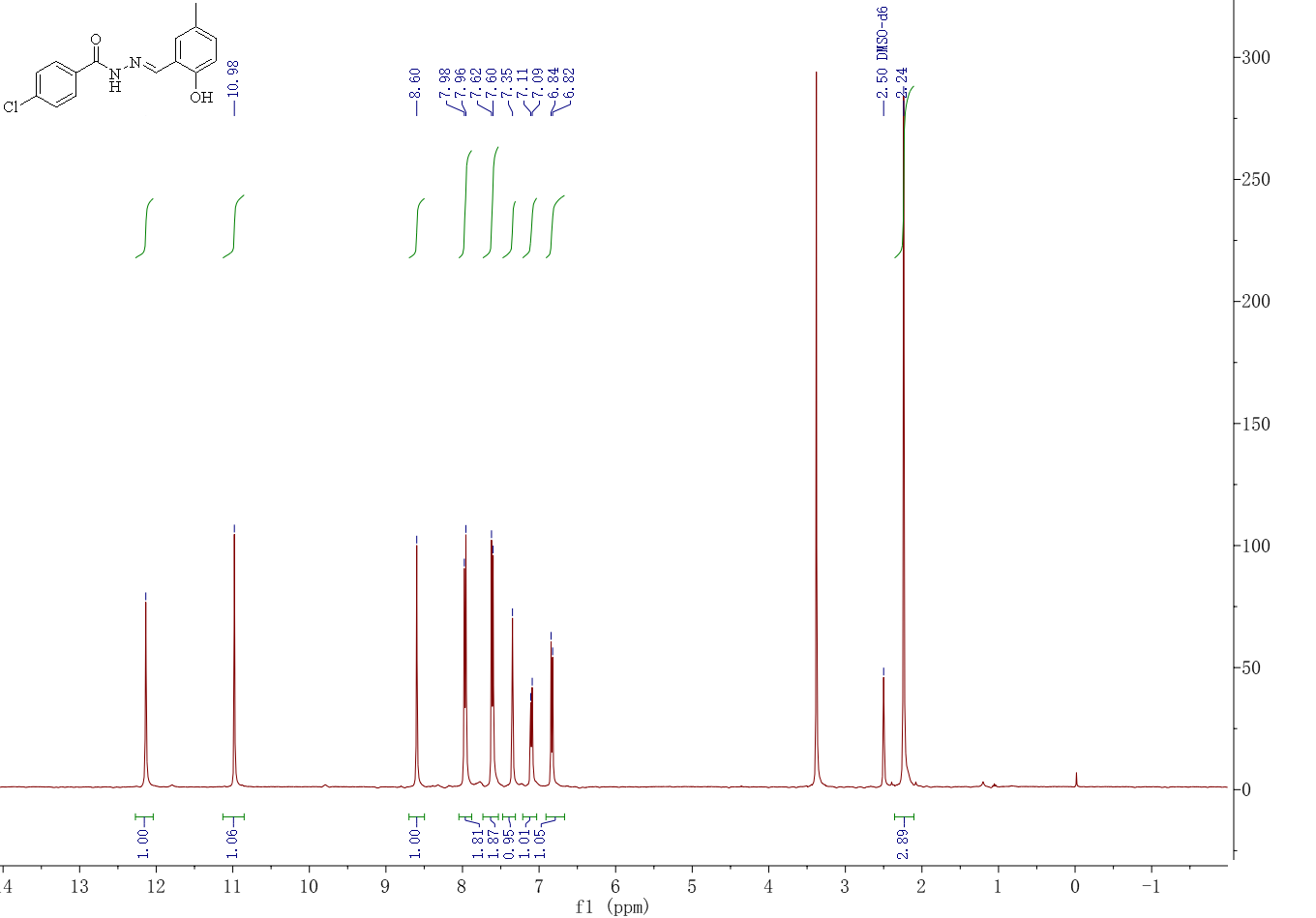


Fig 38. *^1^H NMR of* **C13** (400 MHz, DMSO)


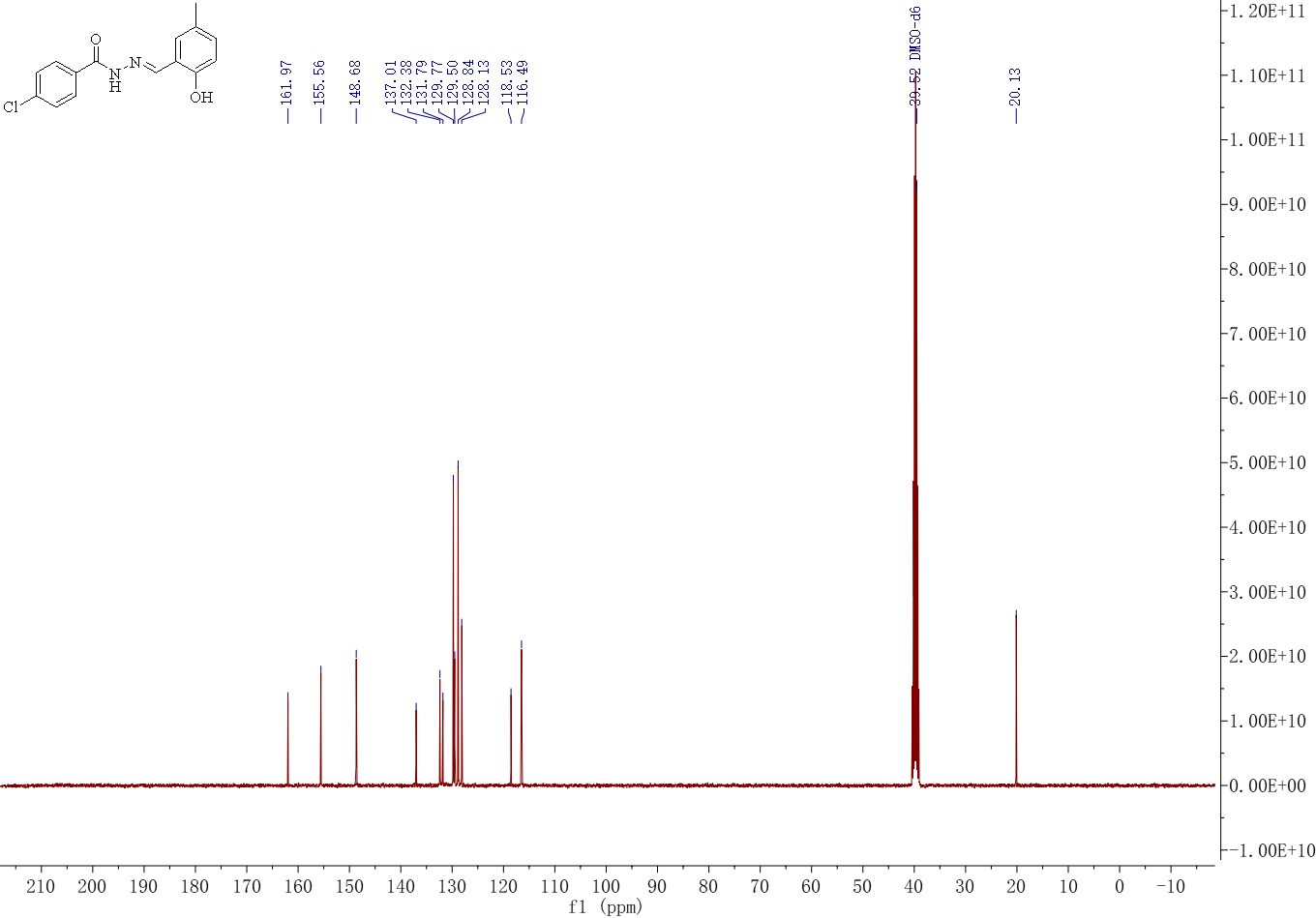


Fig 39. *^13^C NMR of* **C13** (100 MHz, DMSO)


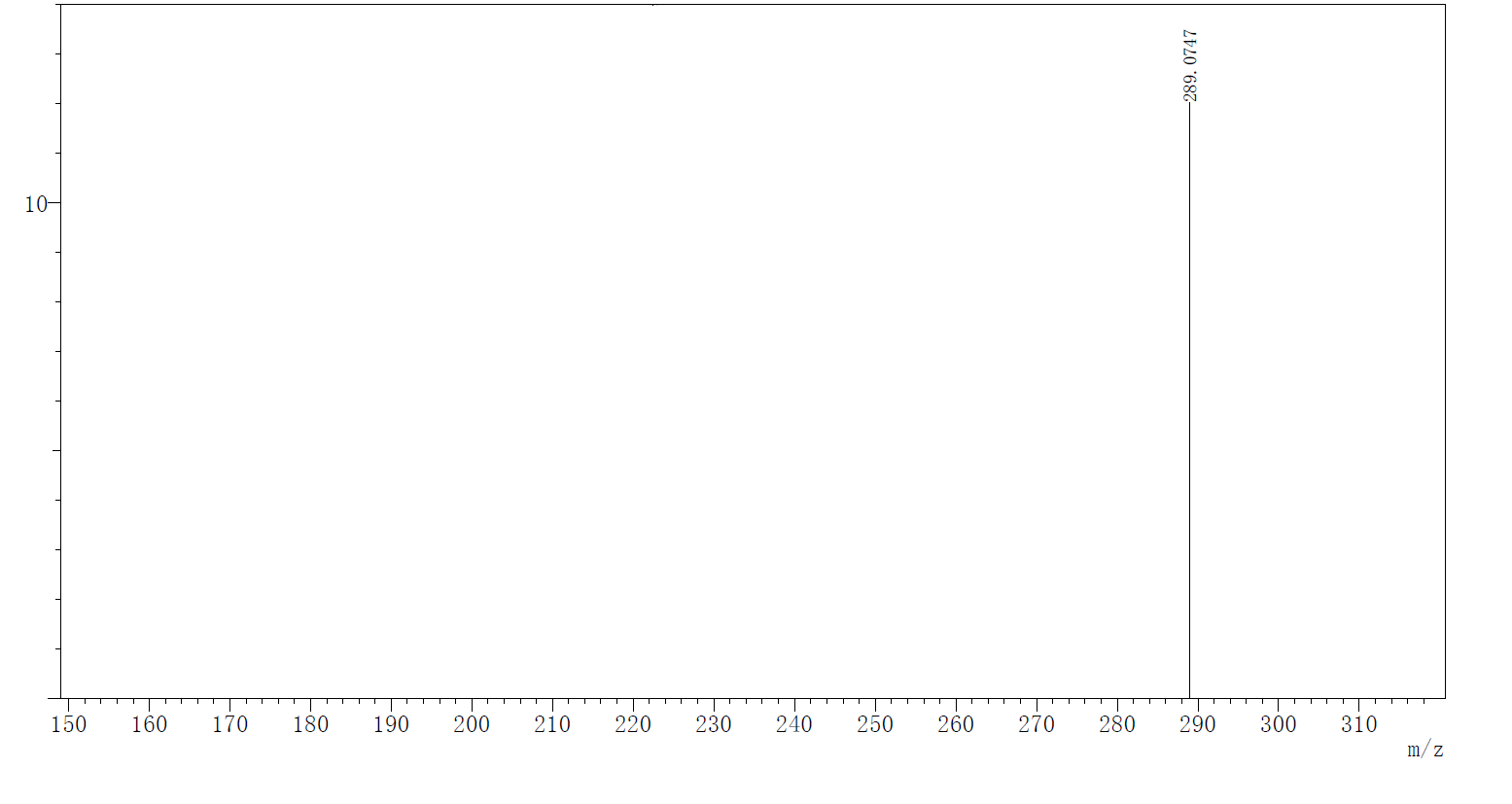


Fig 40. Mass spectrum of compound **C13**


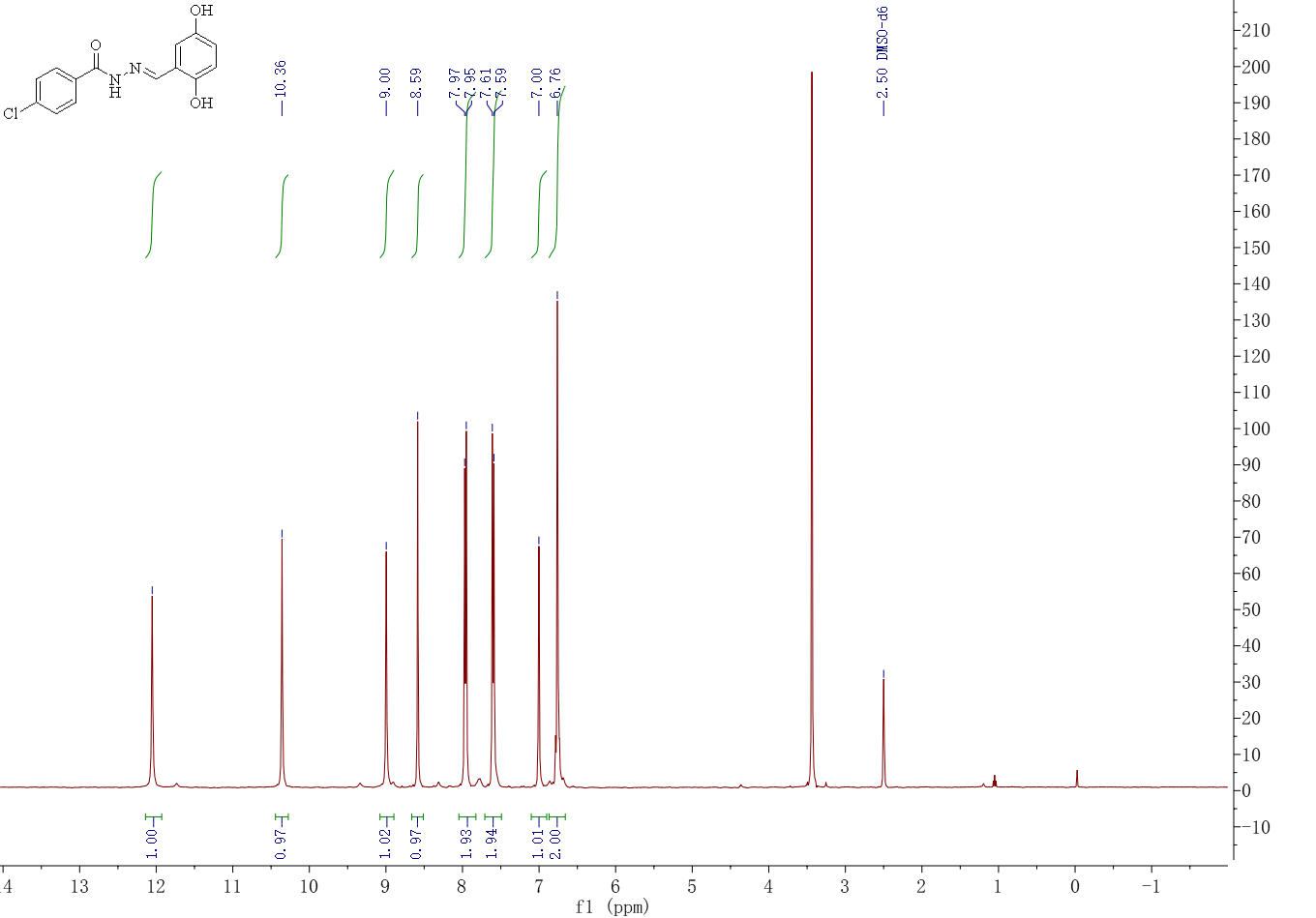


Fig 41. *^1^H NMR of* **C14** (400 MHz, DMSO)


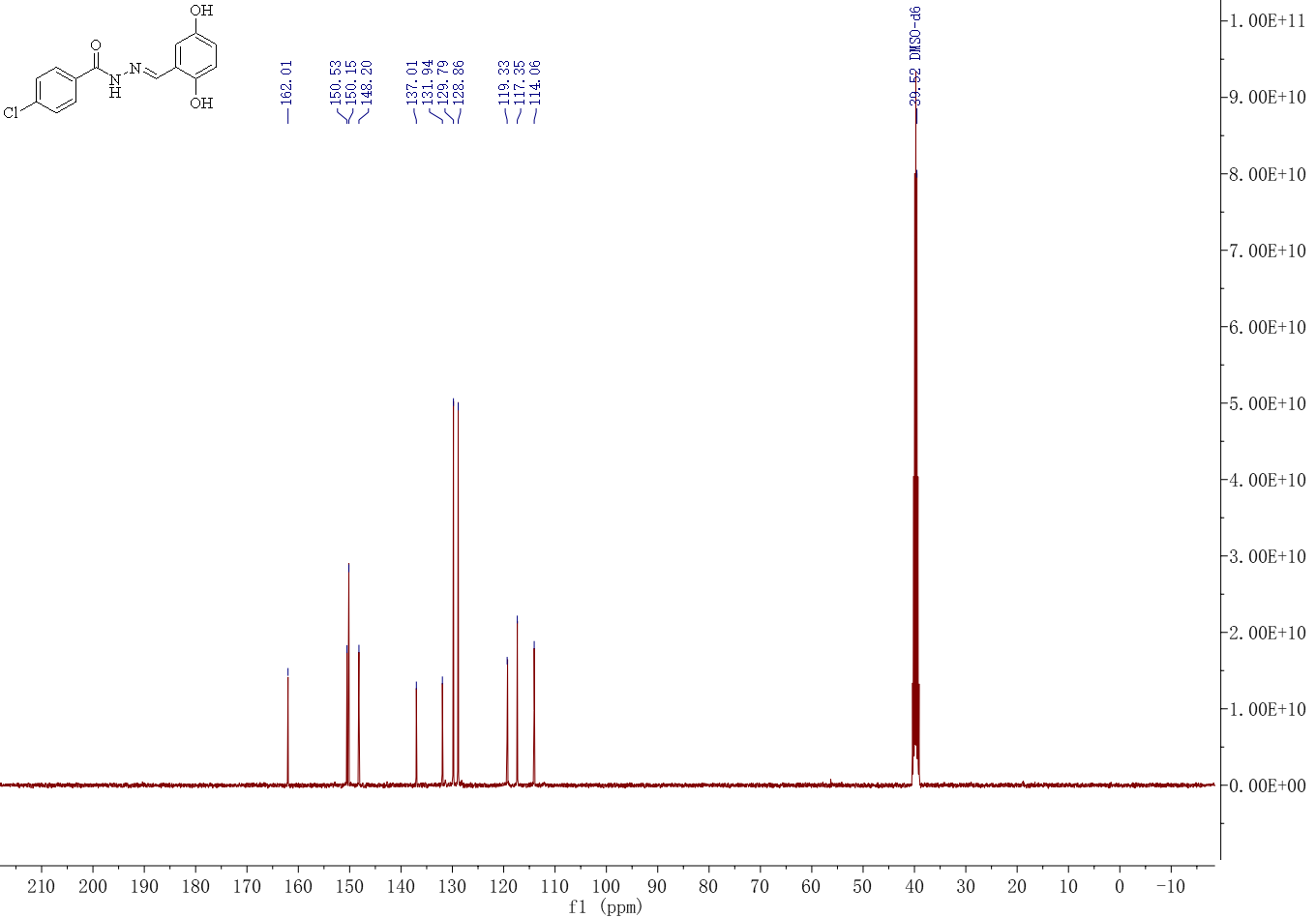


Fig 42. *^13^C NMR of* **C14** (100 MHz, DMSO)


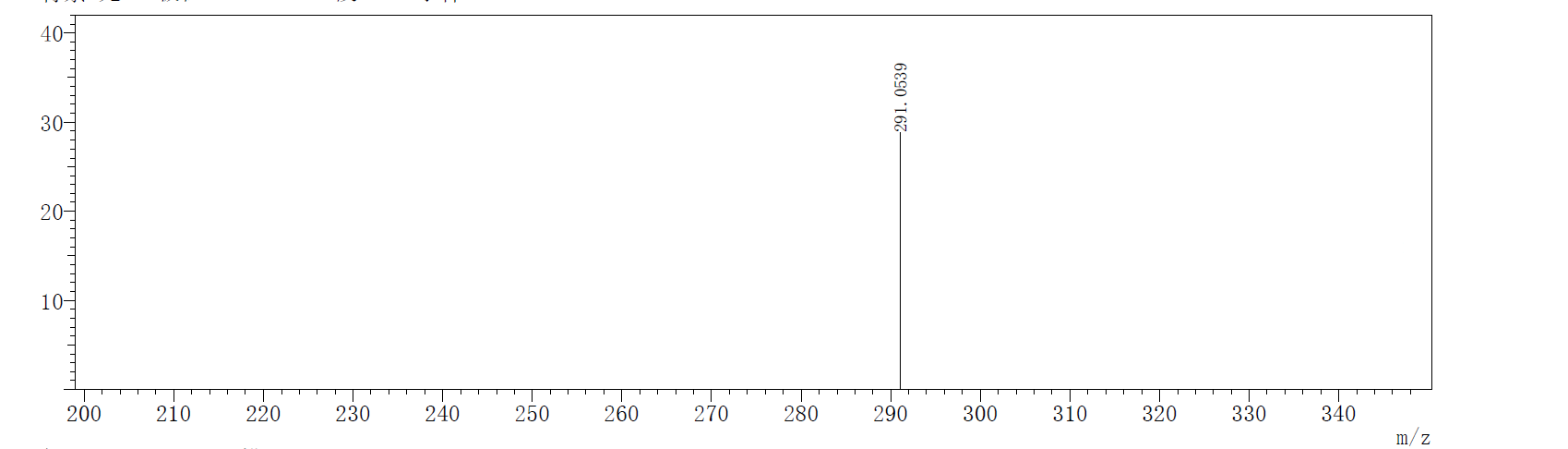


Fig 43. Mass spectrum of compound **C14**


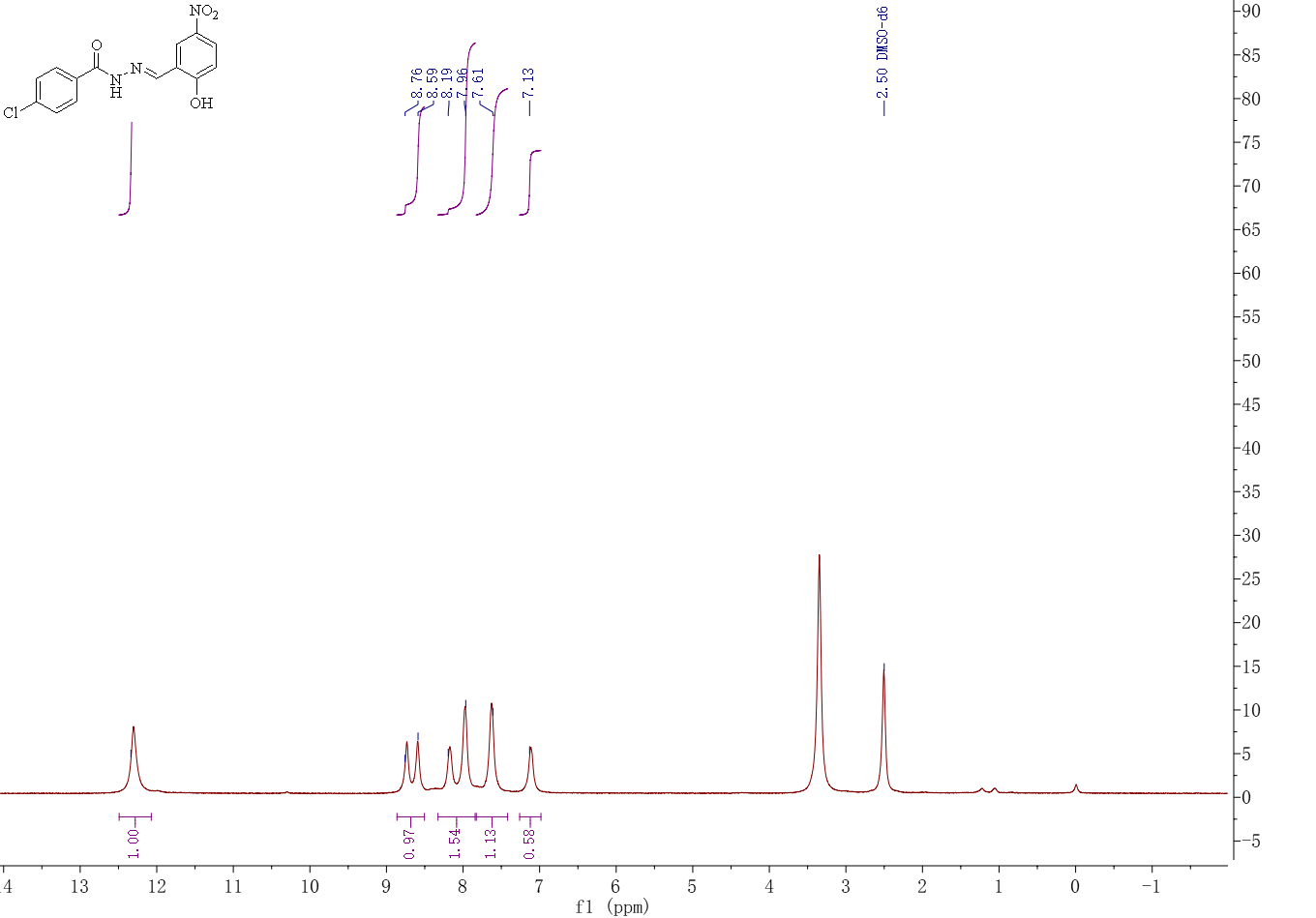


Fig 44. *^1^H NMR of* **C15** (400 MHz, DMSO)


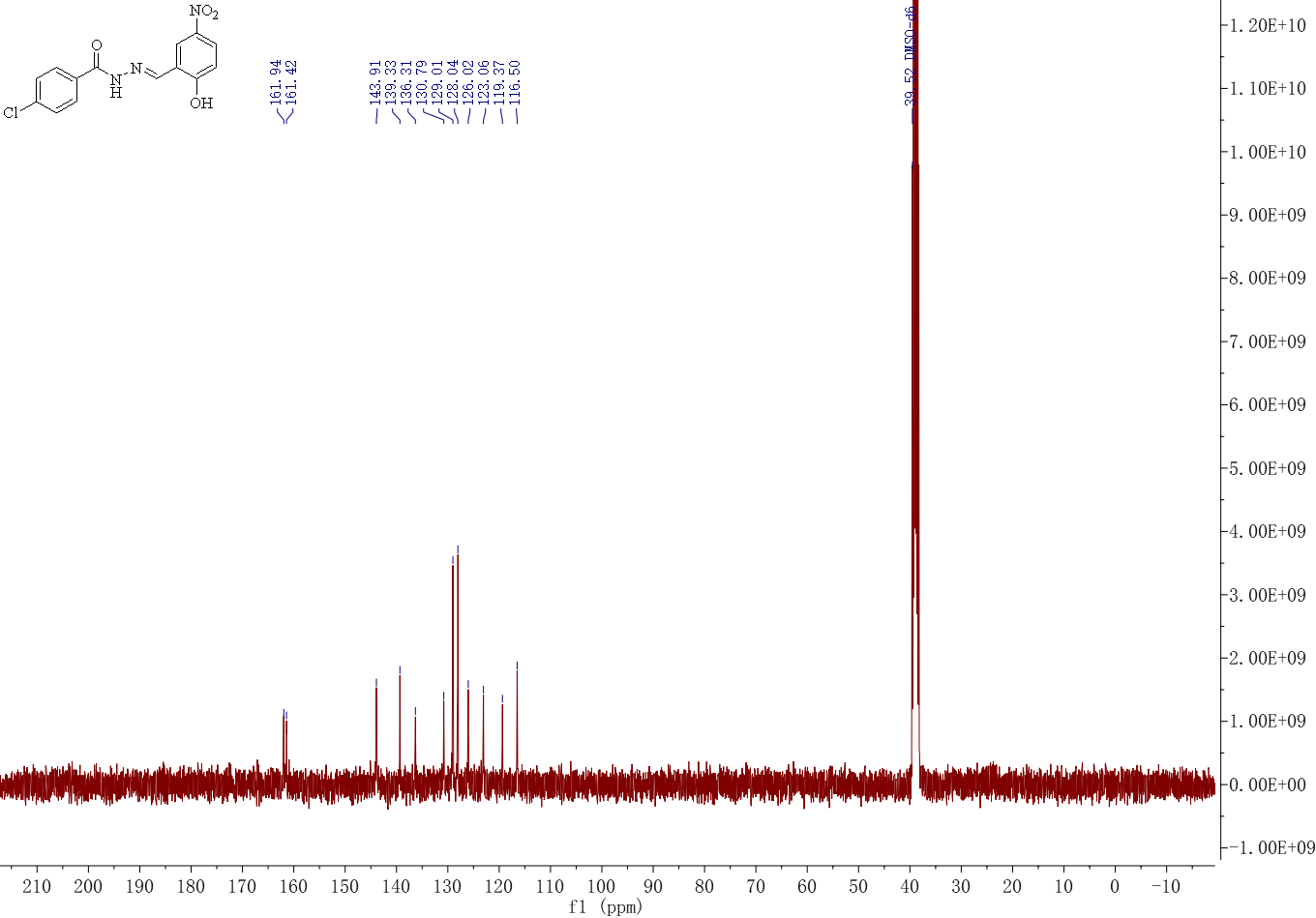


Fig 45. *^13^C NMR of* **C15** (100 MHz, DMSO)


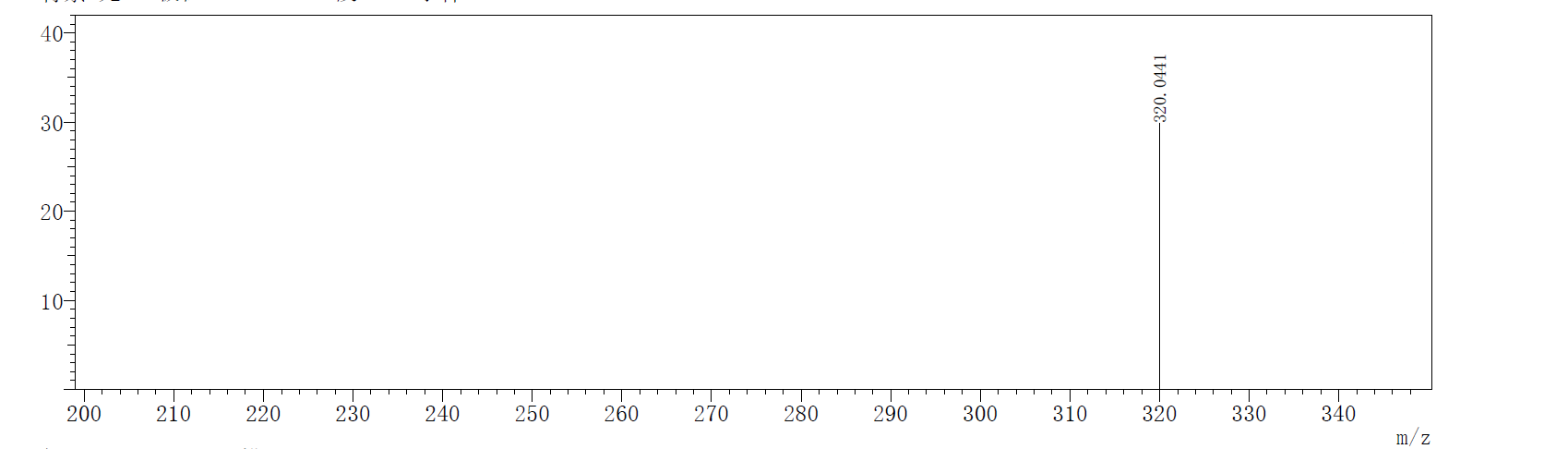


Fig 46. Mass spectrum of compound **C15**


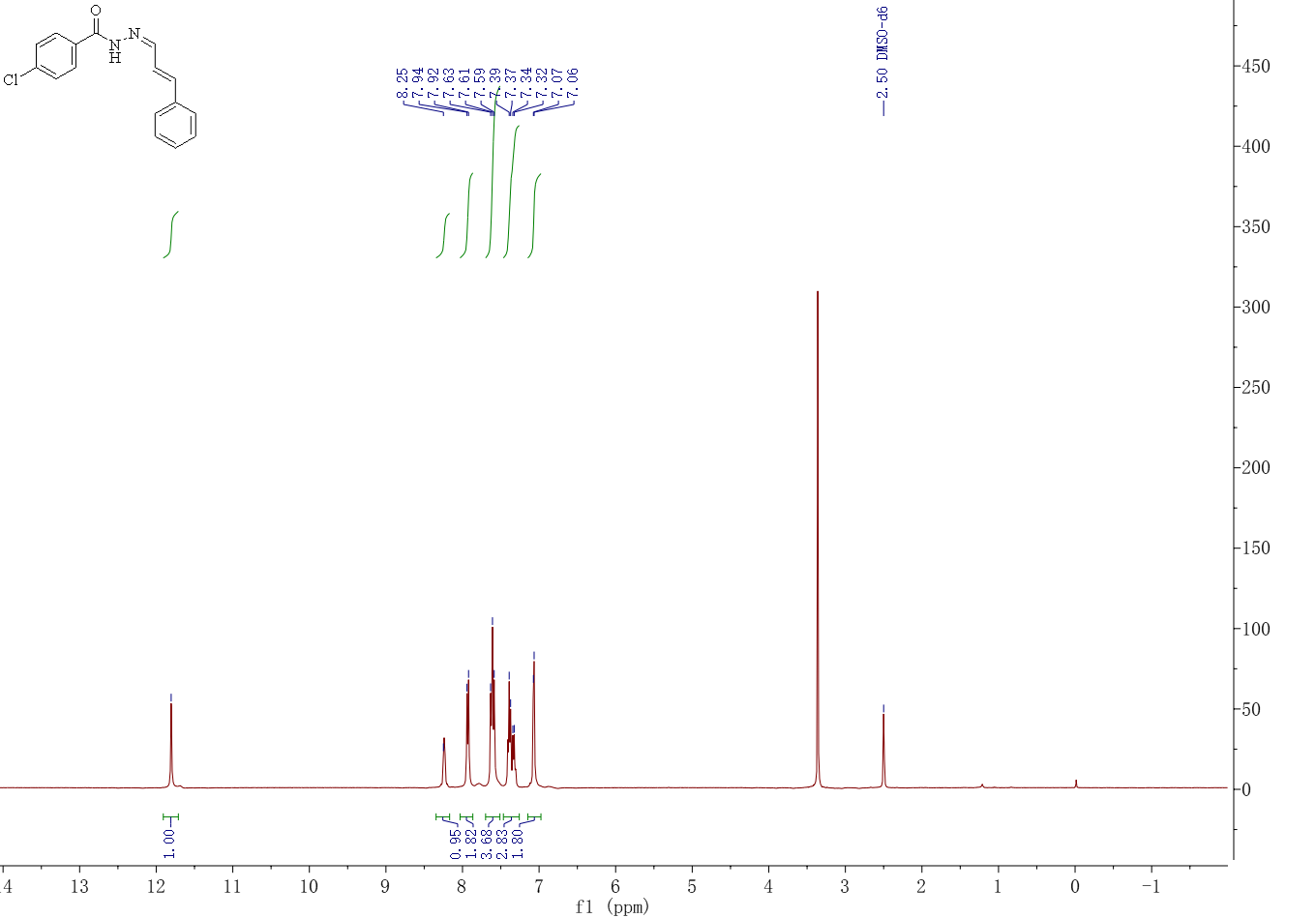


Fig 47. *^1^H NMR of* **C16** (400 MHz, DMSO)


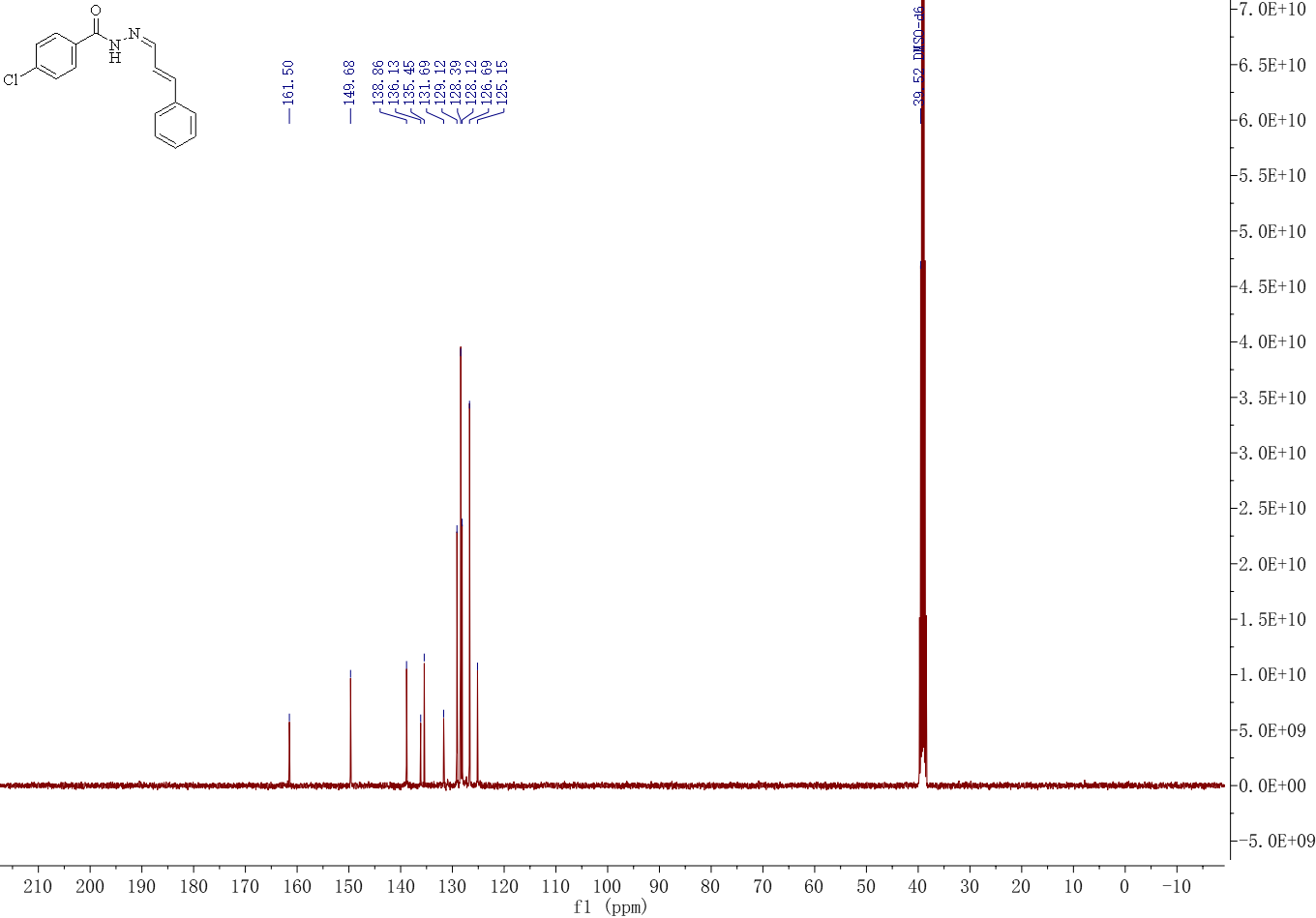


Fig 48. *^13^C NMR of* **C16** (100 MHz, DMSO)


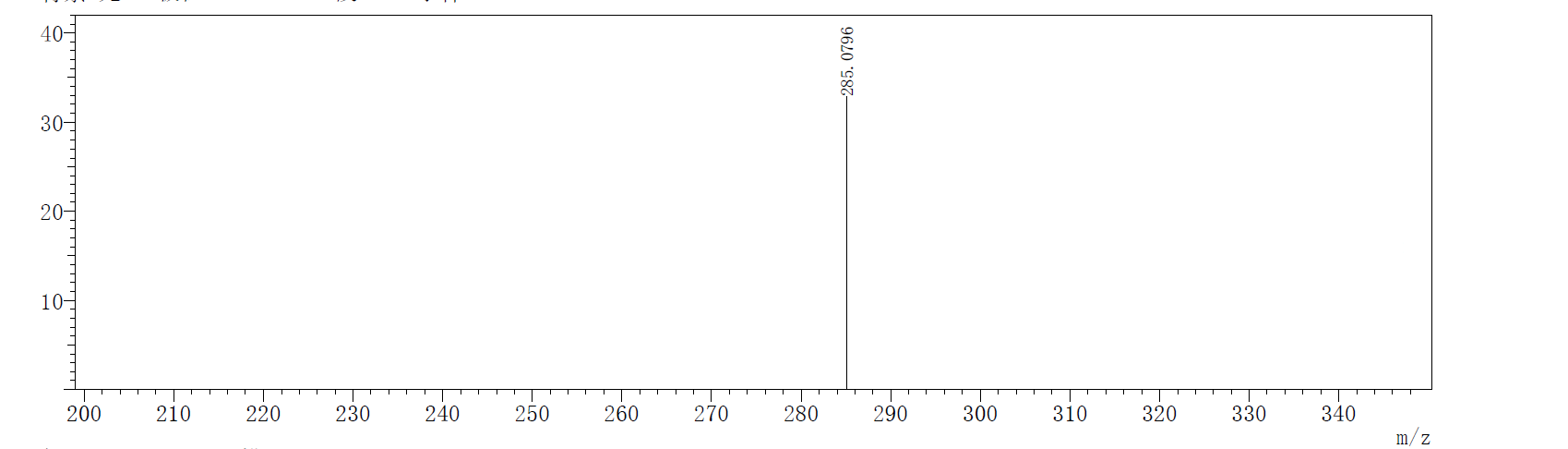


Fig 49. Mass spectrum of compound **C16**


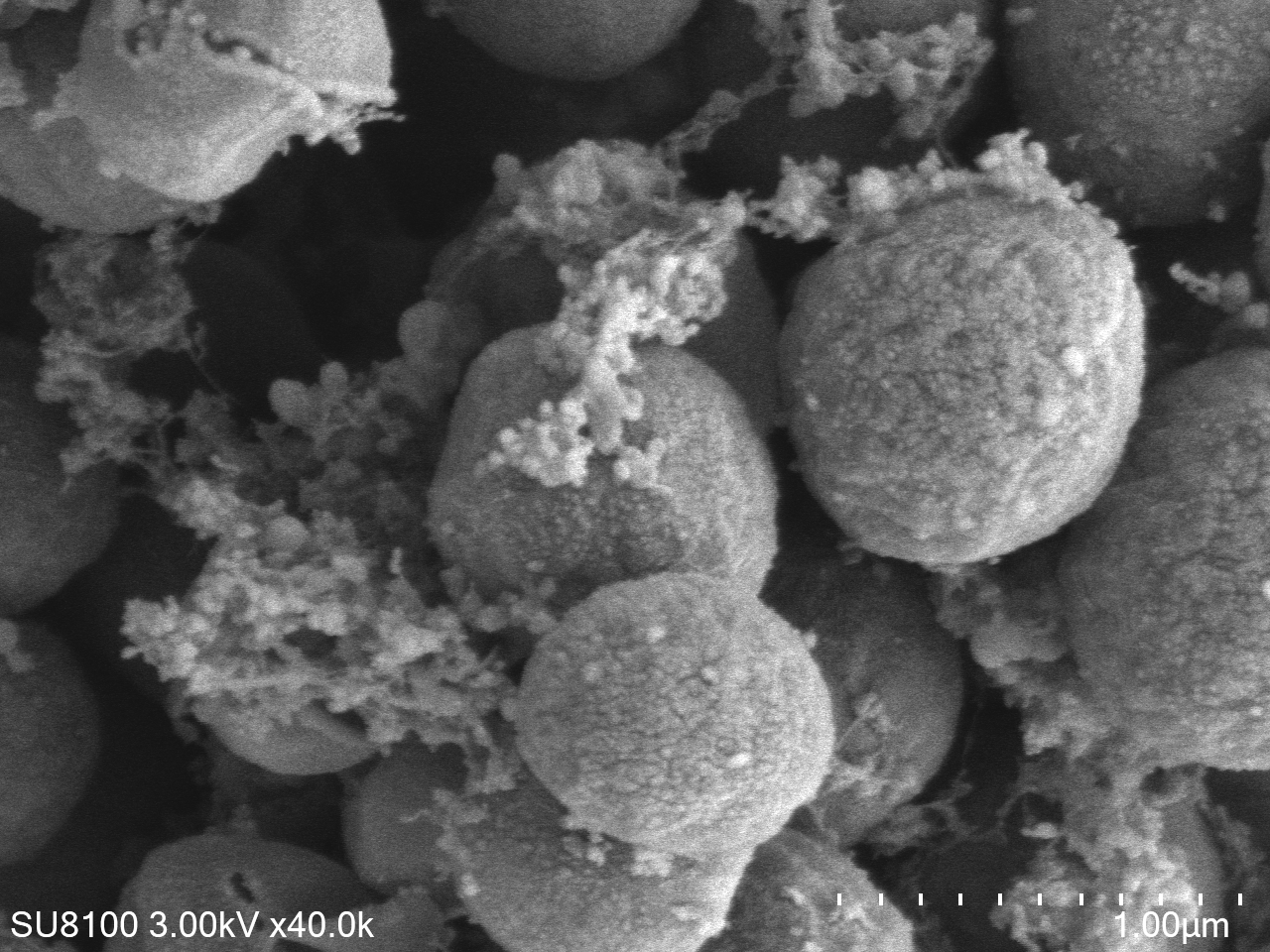


**Figure S1**. Scanning electron microscope (SEM)images (magnify 1000 times) of *S. aureus* MRSA2 treated with **C12**.

**Determination of Minimum Inhibitory Concentration**

The minimum inhibitory concentrations (MICs) of the synthesized compounds were determined using the broth microdilution method, following the recommendations of the Clinical and Laboratory Standards Institute (CLSI). Bacterial strains were cultured in Mueller-Hinton broth (MHB) at 37°C with shaking until the mid-log phase was attained. The resulting suspensions were adjusted to approximately 1 × 10⁵ CFU/mL before being dispensed into sterile 96-well microtiter plates. Serial two-fold dilutions of the test compounds, covering a concentration range from 0.05 to 256 μg/mL, were added to the wells. After 18 hours of incubation at 37°C, the MIC was recorded as the lowest concentration that completely prevented visible bacterial growth. All tests were performed in triplicate on separate occasions to ensure reproducibility.

**Time-Killing Kinetics**

The time-kill kinetics of compound **C12** against *S. aureus* MRSA2 were assessed using the viable plate count method. An overnight bacterial culture was diluted in fresh Mueller-Hinton broth to approximately 1 × 10⁶ CFU/mL and treated with compound **C12** at 4× and 8× the MIC. Samples were collected at specified time points, subjected to serial dilution in sterile saline, and plated onto Mueller-Hinton agar. Following incubation at 37 °C for 18–24 h, viable colonies were counted, with a detection limit of 100 CFU/mL. Time-kill curves were plotted as log₁₀ CFU/mL against time. All assays were conducted in biological triplicate.

**Drug Resistance Study**

The potential for resistance development in *S. aureus* MRSA2 against compound **C12** was assessed via a multi-step serial passaging approach. Initially, the MIC of **C12** was determined by broth microdilution following CLSI guidelines. The strain was subsequently serially passaged for 21 days in Mueller-Hinton broth with incrementally increasing concentrations of **C12**. Each day, bacteria were exposed to sub-inhibitory levels of the compound (0.5× to 2× MIC) and transferred to fresh medium containing the same or a higher drug concentration. Resistance development was defined as a ≥ 8-fold increase in MIC relative to the baseline.

**Hemolysis Assay**

The hemolytic activity of compound **C12** was assessed using a previously described method with minor adjustments. Rabbit erythrocytes, obtained from a commercial supplier as an industry by-product (Solarbio, China), were washed and diluted in PBS to prepare a 4% (v/v) suspension for hemolysis assays. Aliquots (100 µL) of the erythrocyte suspension were combined with 100 µL of **C12** at concentrations ranging from 16 to 256 µg/mL in PBS. Controls included 1% Triton X-100 (100% hemolysis) and PBS alone (spontaneous hemolysis). After incubation at 37 °C for 1 h, samples were centrifuged at 1000 × g for 5 min. Hemoglobin release was determined by measuring the absorbance of the supernatant at 490 nm. Hemolysis percentage was calculated as follows: Hemolysis (%) = [(Abssample − AbsPBS) / (AbsTriton − AbsPBS)] × 100. All experiments were performed in triplicate.

**Cytotoxicity Assay**

The cytotoxicity of the test compounds was determined using the Cell Counting Kit-8 (CCK-8) assay, following the manufacturer's protocol with minor adaptations. In brief, cells were seeded in 96-well plates and allowed to adhere overnight. The commercially obtained VERO cell line (Shang’en Biotechnology) is an immortalized standard model, and its application in this in vitro study complied with relevant guidelines and was exempt from ethical approval. After exposure to a range of compound concentrations for a specified duration, the CCK-8 solution was added, and the plates were incubated for 1–4 hours at 37 °C. Absorbance was measured at 450 nm, and cell viability was calculated as follows: Cell viability (%) = [(OD_sample_ − OD_blank_) / (OD_control_ − OD_blank_)] × 100. All experiments were performed in triplicate.

**Biofilm Inhibition Assay**

The effect of compound **C12** on biofilm formation by *S. aureus* was evaluated using a crystal violet assay. Bacterial cultures were diluted 1:100 in fresh TSB supplemented with 1% glucose and dispensed into wells containing serial concentrations of **C12**. After 24 h of static incubation at 37 °C, the planktonic cells were removed by gentle washing with PBS. The adherent biofilms were then fixed, stained with 0.1% crystal violet, and destained with 95% ethanol. Biofilm biomass was quantified by measuring the absorbance of the solubilized dye at 595 nm. The inhibition percentage was calculated as follows: Inhibition (%) = [(OD_control_ − OD_sample_) / OD_control_] × 100. DMSO was used as the negative control.

**Membrane Depolarization Study**

For the membrane potential assay, *S. aureus* MRSA2 was cultured to mid-log phase in LB broth, collected by centrifugation, washed, and resuspended in PBS to 1 × 10⁸ CFU/mL. Aliquots (150 µL) of the suspension were placed in a black 96-well plate, followed by addition of 40 µL of the membrane potential-sensitive dye DiSC3(5) (10 µM). After 30 min of dark incubation at 37 °C, baseline fluorescence was recorded every 5 min over 40 min. Subsequently, 10 µL of **C12** was added to each well, and fluorescence was monitored for a further 40 min.

Membrane integrity was evaluated using a SYTOX Green uptake assay. Bacterial suspension (150 µL, 10⁸ CFU/mL) was combined with 40 µL of SYTOX Green (3 µM) in a black 96-well plate and incubated for 30 min at 37 °C in the dark. Baseline fluorescence (ex/em: 500/530 nm) was measured every 5 min for 40 min, after which 10 µL of **C12** was introduced to final concentrations of 64 or 256 µg/mL, and fluorescence tracking continued for another 40 min.

**Interaction of C12 with PEG and Cell Membrane Phospholipids**

The interactions between compound **C12** and phospholipids (PE, PG, PGN, CL) were evaluated using a checkerboard broth microdilution method. Serial two-fold dilutions of **C12** (4–256 μg/mL) and each phospholipid (4–256 μg/mL) were prepared directly in 96-well plates. Subsequently, each well was inoculated with an MRSA suspension to a final density of 1 × 10⁵ CFU/mL. The final concentrations of all agents (**C12**, PE, PG, PGN, and CL) in the assay ranged from 1 to 64 μg/mL. The plates were incubated at 37 °C for 18 h, with uninoculated MHB and bacterial suspension in MHB serving as the negative and positive controls, respectively. The MIC of **C12** was determined after the incubation period.

**DNA and Protein Leakage**

MRSA suspensions (2 × 10^6^ CFU/mL) were treated with compound **C12** at final concentrations of 1×, 4×, and 16× MIC. After 4 h of incubation at 37 °C, the samples were centrifuged, and the supernatants were collected for analysis. The released DNA was quantified by microspectrophotometry, while protein content was determined using a BCA Protein Assay Kit.

**ROS Detection Assay**

Intracellular reactive oxygen species (ROS) levels in bacteria treated with compound **C12** were measured using the fluorogenic probe DCFH-DA. Mid-logarithmic phase cultures were harvested, washed with PBS, and adjusted to an OD₆₀₀ of 0.5. The bacterial suspension was then incubated with 10 µM DCFH-DA at 37 °C for 20 min in the dark. After loading and subsequent washing, the labeled cells were resuspended in PBS. Aliquots (190 µL) were transferred to a black 96-well plate and treated with 10 µL of **C12** at specified concentrations. Fluorescence (ex/em = 490/530 nm) was quantified after a 30 min incubation at 37 °C.

**Plasma Protein Binding Rate of C12**

The plasma protein binding of compound **C12** was evaluated by equilibrium dialysis. Pre-hydrated dialysis bags containing 0.5 mL of blank rat plasma were immersed in centrifuge tubes filled with 40 mL of PBS dialysate spiked with 30 μM **C12**. The assembly was incubated at 37 °C with shaking at 100 rpm for 24 h to reach equilibrium. Subsequently, samples from both the plasma and dialysate compartments were collected and analyzed by HPLC. The plasma protein binding rate was calculated as: Binding (%) = [(A − B) / A] × 100, where A is the total drug concentration inside the bag (plasma) and B is the free drug concentration in the dialysate. All assays included nonspecific binding controls and were performed in triplicate.

**Determination of logD_7.4_ for C12**

The logD_7.4_ of compound **C12** was determined using the shake-flask method. A solution of **C12** (1 mg) in 2 mL of pH 7.4 buffer-saturated n-octanol was combined with an equal volume of n-octanol-saturated buffer. The mixture was vortexed for 3 min and then equilibrated at 37 °C with shaking (200 rpm) for 24 h. After phase separation by centrifugation, the n-octanol and aqueous phases were sampled, diluted appropriately in methanol, and analyzed by HPLC. LogD_7.4_ was calculated as log[(peak area in octanol × x) / (peak area in buffer × y)]. All measurements were performed in triplicate.

**Liver microsomal Stability Assay for C12**

The metabolic stability of compound **C12** was assessed in rat liver microsomes. The incubation system consisted of 0.5 mg/mL microsomal protein, 100 μM **C12**, and 20 mM NADPH in 0.1 M PBS (pH 7.4). The reaction was carried out at 37 °C, and aliquots were collected at predetermined time points over 120 min and quenched with ice-cold acetonitrile. After vortexing and centrifugation, the supernatants were evaporated under nitrogen, reconstituted in 200 μL of methanol, and analyzed by HPLC. The percentage of the parent compound remaining at each time point was quantified to evaluate metabolic stability. All samples were processed in triplicate.

**Molecular docking**

Ligand preparation. The ligand was drawn in InDraw and saved as an SDF file. Ligand preparation for docking was performed using Meeko (v0.5.0) to generate an AutoDock-compatible representation and export the ligand in PDBQT format. The ligand file was subsequently inspected in AutoDockTools (ADT; MGLTools v1.5.7p1) to ensure consistent atom typing/rotatable-bond definitions and charge handling within the AutoDock/Vina workflow. Receptor preparation. The target receptor structure (PDB ID: 1VQQ) was prepared using AutoDockTools (MGLTools v1.5.7p1) by removing non-receptor molecules as appropriate, adding polar hydrogens, assigning Gasteiger charges, and exporting the receptor in PDBQT format. Docking protocol. Docking calculations were performed using AutoDock Vina (v1.1.2). The docking search space was defined as a rectangular grid box centered on the FAD region (grid center coordinates measured in PyMOL): center_x = 15.139 Å, center_y = 27.556 Å, center_z = 38.500 Å, with box dimensions size_x = 100 Å, size_y = 90 Å, size_z = 102 Å. Docking was conducted with exhaustiveness = 32, num_modes = 20, and energy_range = 3 kcal/mol. The resulting poses were ranked by Vina’s scoring function, and the top-ranked pose (lowest predicted affinity) was selected as the representative binding mode. The best docking score obtained was −5.811 kcal/mol. Pose inspection and visualization. The selected protein–ligand complex was visually inspected and rendered in PyMOL to confirm reasonable placement within the intended pocket and to support interaction analysis and figure generation.

**Molecular Dynamics Simulations**

The initial conformation of the protein–ligand complex was sourced from the docking results. An explicit solvent simulation system was constructed and GROMACS input files were generated using the CHARMM-GUI Solution Builder. The CHARMM36m force field was applied to the protein. Ligand force field parameters were automatically assigned by the CGenFF server and subsequently converted into a GROMACS-compatible format. The system was placed in a periodic boundary conditions (PBC) orthorhombic water box with the TIP3P water model. Electrical neutrality (and the required ionic strength as per CHARMM-GUI settings) was achieved by adding K⁺ and Cl⁻ ions. The final system comprised approximately 3.45 × 10⁵ atoms (protein: ~1.02 × 10⁴ atoms; ligand: 32 atoms; the remainder being water molecules and ions).

All simulations were performed using GROMACS 2025.3. Long-range electrostatic interactions were treated with the Particle Mesh Ewald (PME) method. Non-bonded interactions were handled using the Verlet cutoff scheme, with cutoff and neighbor-list parameters set according to the `.mdp` file generated by CHARMM-GUI. Bonds involving hydrogen were constrained using the LINCS algorithm, and water geometry was constrained using SETTLE. The system first underwent energy minimization via the steepest descent method until the maximum force met the convergence criterion (`Fmax` < 1000 kJ·mol⁻¹·nm⁻¹ for this system). Subsequently, a multi-step equilibration protocol, as recommended by CHARMM-GUI, was followed (involving the gradual release of positional restraints and NVT/NPT equilibration; specific temperature/pressure coupling schemes and time constants were set in the `.mdp` file). Finally, a 200 ns production molecular dynamics simulation was conducted. The production simulation employed an integration time step of 2 fs (`dt` = 0.002 ps; totaling ~1 × 10⁸ steps), with the temperature maintained at 303.15 K and pressure at 1 bar (as specified in the `.mdp` file). Simulations were run on an Ubuntu 22.04 platform and accelerated by an RTX 4090D GPU (Particle-Particle and PME calculations were executed on the GPU, while constraints and coordinate updates were handled by the CPU).

Trajectory Processing and Analyses

To eliminate molecular discontinuities and overall system drift caused by PBC, trajectories were first processed using the `trjconv` tool for molecular reconstruction and centering (`-pbc mol -center`), thereby generating PBC-free trajectories for subsequent analysis.

Protein conformational stability was assessed using: i) protein backbone RMSD (calculated after least-squares fitting), ii) residue-wise RMSF, iii) radius of gyration (Rg), and iv) solvent-accessible surface area (SASA).

Ligand binding stability was characterized by: i) the minimum distance between the ligand and protein, ii) the number of contacts within a 0.35 nm threshold, and iii) the number of hydrogen bonds between the ligand and protein.

All time-dependent plots are presented with nanoseconds (ns) as the common x-axis unit.

1. *Correspondent. E-mail: [h418561754@163.com](mailto:h418561754@163.com) [↑](#footnote-ref-2)
